# Phage-inducible chromosomal islands are ubiquitous within the bacterial universe

**DOI:** 10.1038/s41396-018-0156-3

**Published:** 2018-06-06

**Authors:** Alfred Fillol-Salom, Roser Martínez-Rubio, Rezheen F. Abdulrahman, John Chen, Robert Davies, José R. Penadés

**Affiliations:** 1 Institute of Infection, Immunity and Inflammation, College of Medical, Veterinary and Life Sciences, University of Glasgow, Glasgow, G12 8TA UK; 2 Departamento de Ciencias Biomédicas, Universidad CEU Cardenal Herrera, 46113 Moncada, Valencia Spain; 3 Department of Microbiology and Immunology, Yong Loo Lin School of Medicine, National University of Singapore, 5 Science Drive 2, Singapore, Singapore

## Abstract

Phage-inducible chromosomal islands (PICIs) are a recently discovered family of pathogenicity islands that contribute substantively to horizontal gene transfer, host adaptation and virulence in Gram-positive cocci. Here we report that similar elements also occur widely in Gram-negative bacteria. As with the PICIs from Gram-positive cocci, their uniqueness is defined by a constellation of features: unique and specific attachment sites, exclusive PICI genes, a phage-dependent mechanism of induction, conserved replication origin organization, convergent mechanisms of phage interference, and specific packaging of PICI DNA into phage-like infectious particles, resulting in very high transfer frequencies. We suggest that the PICIs represent two or more distinct lineages, have spread widely throughout the bacterial world, and have diverged much more slowly than their host organisms or their prophage cousins. Overall, these findings represent the discovery of a universal class of mobile genetic elements.

## Introduction

The *Staphylococcus aureus* pathogenicity islands (SaPIs) are a novel class of phage satellites that are intimately related to certain temperate (helper) phages, of whose life cycles they parasitize. Following infection by a helper phage or SOS induction of a helper prophage, the SaPI genome excises from the bacterial chromosome, using SaPI-encoded integrases (*int)* and excision functions (*xis*) [1, 2], replicates extensively using its own replicon [3], and is efficiently packaged into infectious particles composed of phage virion proteins [4–6]. Some SaPIs encode capsid morphogenesis functions that remodel the phage capsid to only fit their smaller genomes [7, 8]; others simply use full-size phage capsids (for a recent review, see [9]). The hallmark of this parasitism is a key SaPI regulatory gene that diverts the phage reproduction cycle to its own end. This gene encodes a master repressor (Stl) that governs expression of the SaPI genome [10]. Unlike the classical phage repressor, the SaPI Stl repressor is not cleaved following activation of the SOS response; rather the repression is lifted by the formation of a complex between the repressor and a specific helper phage protein [11–13]. This serves to couple the SaPI life cycle with that of the helper phage, ensuring that the SaPI is not activated unless the reproductive cycle of a helper phage is in progress. Once induced, different SaPIs use different strategies to initiate specific packaging from the cognate SaPI genome [7, 14], ensuring their high intra- and inter-generic transfer [15–17]. Moreover, SaPIs have a huge impact on the biology of their helper phages by interfering with phage reproduction using different and complementary strategies [18–20]; they are also key elements driving phage evolution [21].

**Fig. 1:**
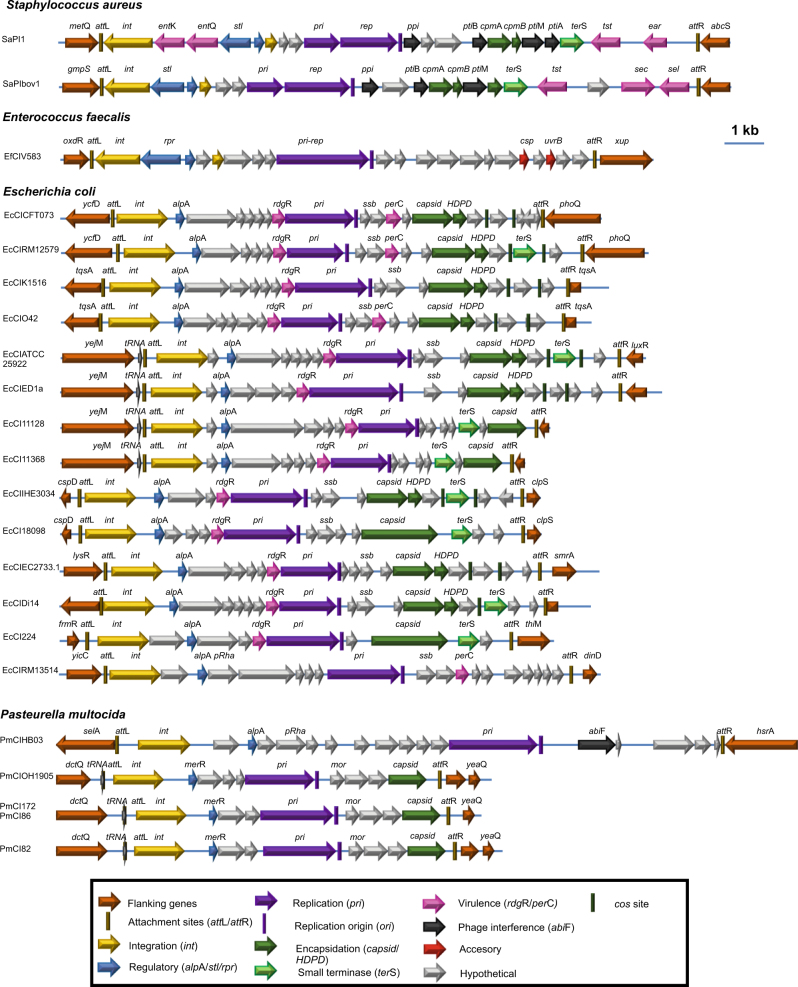
Genome maps for PICIs. Genomes are aligned according to the prophage convention, with the integrase gene (*int*) at the left end. Genes are colored according to their sequence and function: *int* is yellow; transcription regulator (*stl* or *rpr* (GP), and *alp*A or *mer*R (GN)) is dark blue; replication genes are purple; encapsidation genes are green, with the terminase small subunit gene (*ter*S) in light green; superantigen and other virulence genes are pink; genes encoding putative phage resistance proteins are black; other accessory genes are red; genes encoding hypothetical proteins are white

Not surprisingly, SaPI-like elements are not unique to staphylococci, and we have recently demonstrated that they are widespread in Gram-positive (GP) cocci [22]. This new family of mobile genetic elements (MGEs), that we have called generically phage-inducible chromosomal islands (PICIs), has very well conserved features [9, 23]. All PICIs have a conserved gene organization (Fig. 1), and encode a pair of divergent regulatory genes, including the PICI master repressor Rpr (called Stl in the SaPIs). Left of *rpr*, and transcribed in the same direction, PICIs encode a small set of genes including an integrase (*int*) gene. Right of *rpr*, and transcribed in the opposite direction, the PICIs encode an excision function (*xis*), and a replication module consisting of a primase homolog (*pri*) and a replication initiator (*rep*), which are sometimes fused, followed by a replication origin (*ori*). Next to these genes, and also transcribed in the same direction, PICIs encode genes involved in phage interference, and sometimes, a terminase small subunit homolog (*ter*S) which is responsible for the high efficiency of the SaPI packaging [7]. In the SaPIs, accessory genes (usually involved in virulence; [9]) can be found either at the 3′ end of the elements or between the *int* and *stl* genes (Fig. 1).

In addition to this well-conserved gene organization, the GP PICIs have other common features that distinguish them from their cousin prophages: (i) unique attachment (*att*) sites that are never occupied by prophages; (ii) the absence of phage structural and lytic genes; (iii) size typically around 15 kb; (iv) sophisticated strategies to interfere with helper phage reproduction and (v) genes unique to PICIs. Orthology analyses show that PICI genes belong to large sets of orthologs, within which the first 8–10 are almost always PICI genes, most of which never appear in other genetic elements [22, 24].

The preliminary analysis of the PICIs present in the GP cocci suggested that these elements could have evolved de novo in the different genera [22], suggesting their existence is strongly selected in nature. Therefore, and as happens with the classical MGEs, we hypothesized that these elements are likely not just confined to the GP cocci but widespread in nature. We now report the identification and characterization of a large number of PICI elements in various Gram-negative (GN) bacteria. Because they have certain key features in common and because they are so widespread, we suggest that their lifestyle may have strong selective value and that they represent a novel agency of horizontal dissemination of virulence and other important mobile genes among bacteria.

## Results

### Identification of PICI candidates

Since PICIs are a successful biological strategy in GP cocci, we hypothesized that similar elements will be widespread in GN bacteria. Using the aforementioned criteria defining the PICI elements in the cocci (see material and methods for more details), we have assembled a representative collection of putative PICIs in the GN bacteria by genome searching; their genomes and characteristics are depicted in Fig. 1 and S1, and in Table S1. Following the nomenclature proposed for the elements present in the GP bacteria, the individual PICIs are designated with reference to their species – thus, EcCIn or PmCIn would be used for PICIs of *Escherichia coli* or *Pasteurella multocida*, respectively, where “*n*” would be used for the specific PICI-containing strain.

### Features of PICI candidates

**Fig. 2:**
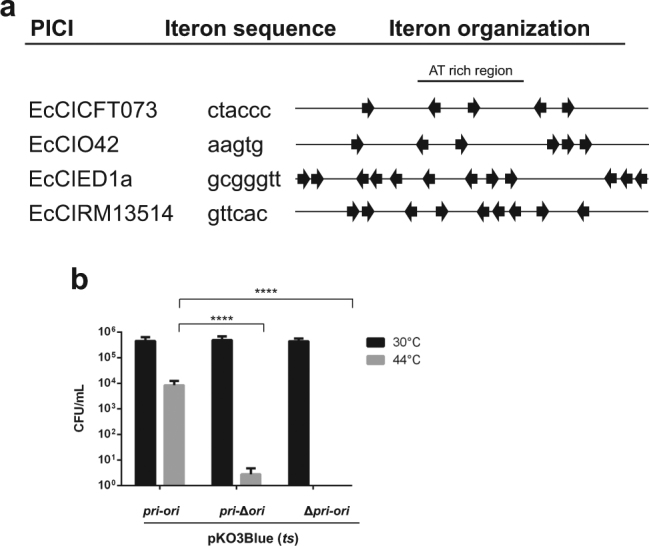
Characterization of GN PICI replication origins. **a** Comparative map of the replication origins of several *E. coli* PICIs. The iterons are represented by arrows, and their sequences are shown at left. Note that there are always two sets of iterons flanking an AT rich region, which could be the melting site. **b** Testing EcCICFT073 *pri-rep-ori* function. pK03Blue derivative plasmids *pri*-*ori*, *pri*-∆*ori* or ∆*pri*-*ori* were tested for maintenance in DH5α on selective agar at the permissive (30 °C) or restrictive (44 °C) temperature. A One-way ANOVA with Tukey’s multiple comparisons test was performed to compare mean differences within rows. Adjusted *p* values were as follows: *pri-ori* 44 °C vs *pri-∆ori* 44 °C < 0.0001^****^, *pri-ori* 44 °C vs *∆pri-ori* 44 °C < 0.0001^****^

The putative PICIs described here have a number of features in common. (i) Occurrence: they are very common among GN bacteria, especially in members of the *Enterobacteriaeae* and *Pastuerellaceae* (*Gammaproteobacteria*) (Table S1). (ii) Exclusivity: the KEGG orthology analyses confirmed that the GN PICI-encoded genes are PICI specific, not being present in other families of MGEs (Tables S2-S4). These analyses were performed using three unrelated PICI elements, two from *E. coli*, EcCICFT073 and EcCIO42, and one from *P. multocida*, PmCIATCC43137, as representatives of the GN PICIs. (iii) Integration: in each analyzed species, the PICI *att*_C_ sites are never used by temperate prophages (Fig. S2, Table S1). (iv) *Transcriptional organization*: contrary to the PICIs from the GP bacteria, the PICIs in the GN species are transcribed unidirectionally and rightward, unlike the vast majority of temperate phages but similar to a few (for example, phages Mu or Sp18, in *E. coli* O157 Sakai [25]). This organizational differentiation suggests that the elements from GN and GP bacteria comprise at least two different lineages. (v) Replication origins: The region immediately 3′ to the replication initiation gene in the GP PICIs represents the replication origin [3]. Examination of this region for several of the PICIs from the GN organisms reveals an organization and functionality identical to that of the GP PICIs (Fig. 2a). (vi) Capsid morphogenesis: some of the elements encode homologs of the PICI capsid morphogenesis genes (Fig. 1). (vii) Interference: The two PICIs analyzed in detail show interference mechanisms similar to those previously identified in the SaPIs and in other PICI elements from Gram-positive bacteria, representing a fascinating example of convergent evolution (see below). (viii) Accessory genes: Many of the putative PICIs carry identifiable genes that do not appear to be involved in the PICI lifecycle. These accessory genes are carried exclusively by the PICIs. As noted, some are known to be involved in virulence.

### Induction and transfer

GP PICI DNA is packaged in particles composed of phage virion proteins [5, 6]. While some GP PICIs express a small terminase subunit (TerS) homolog [7], others are highly packaged because they carry the phage *pac* or *cos* sequence [14, 22]. Some GP PICIs direct the formation of capsids 1/3 the size of the helper phage capsid, using different strategies [7, 8, 22, 26, 27]. Some, but not all, of the newly identified PICIs also contain a *ter*S homolog (shown in light green); we have to date identified both the helper phages and the interfering mechanisms for two of these: EcCICFT073 and PmCI172.

### EcCICFT073

**Fig. 3:**
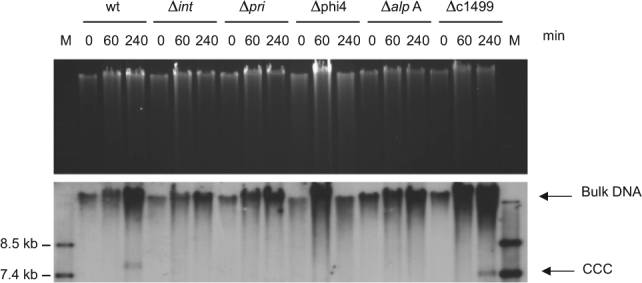
Phage induction of EcCICFT073. CFT073 strains were SOS induced with MC (2 μg/ml). Samples were removed at the indicated time points and used to prepare minilysates, which were resolved on an 0.7% agarose gel (upper panel), and Southern blotted (lower panel) with an EcCICFT073 probe. M: Southern blot molecular marker (DNA molecular weight marker VII; Roche)

Since our genomic scrutiny had revealed the presence of putative PICI-like elements in different *E. coli* strains, we wanted to characterize some of them in more detail. For that, we inserted a *cat* marker into the elements present in strains IHE3034, 18098 and O42, and a *tet*A marker into the element present in the CFT073 strain. The localization of the antibiotic resistant markers in the PICI elements is shown in Fig. S3. While the two first islands encode TerS, the last two do not. The strains carrying the putative GN PICIs were treated with mitomycin C (MC) and the transfer of these elements after induction of the resident prophages was tested. As shown in Table 1, the EcCICFT073 element, but not the other three, was transferred at high frequency to the non-lysogenic DH5α (*rec*A mutant), C600, 594 and WG5 strains. Moreover, MC induction of a CFT073 culture resulted in PICI DNA amplification, although no “PICI monomer” band appeared (Fig. 3). There was, however, a band containing covalently closed circular (CCC) DNA, indicative of PICI excision. On the basis of the following tests, we suggest that these results are indicative of helper phage induction of PICI excision, replication and packaging: we inserted a *tet*A marker into the chromosomal *lac* gene of the EcCICFT073 host strain, the uropathogenic CFT073 strain, and tested the transfer of this marker by the resident prophages. Contrary to the transfer observed for the EcCICFT073 *tet*A element, we did not detect transfer of the chromosomal *tet*A to the recipient strains, suggesting that none of the resident prophages is a generalized transducing phage. To further confirm that the transfer of EcCICFT073 *tet*A was PICI-specific rather than generalized transduction, we mutationally inactivated the EcCICFT073 *tet*A *int* gene and found that this abolished detectable excision and transfer (Fig. 3; Table 1). We also mutated the *pri* gene and found that this greatly reduced but did not completely eliminate transfer (Fig. 3; Table 1).

**Table 1:** Effects of PICI and phage mutations on PICI transfer^a^

	Recipient strain^a^			
Donor strain	C600	WG5	DH5α	594
CFT073 c1501::*tet*A	8 × 10^3^	3.4 × 10^3^	4.07 × 10^3^	4.2 × 10^3^
CFT073 *lac*Z::*tet*A	< 1	< 1	< 1	< 1
CFT073 c1499::*cat*	1 × 10^5^	3.6 × 10^5^	2 × 10^5^	2.1 × 10^5^
IHE3034 RS04635-RS04640::*cat*	< 1	40	< 1	< 1
O42 1749::*cat*	< 1	< 1	< 1	< 1
18098 ORF17-ORF18::*cat*	80	110	< 1	10
PICICFT073 mutants				
CFT073 c1501::*tet*A ∆*int*	< 1	< 1	< 1	< 1
CFT073 c1501::*tet*A ∆*alp*A	< 1	< 1	< 1	< 1
CFT073 c1501::*tet*A ∆*pri-rep*	50	87	45	37
CFT073 c1501::*tet*A ∆c1499	3.8 × 10^3^	1.64 × 10^3^	2.37 × 10^3^	2.5 × 10^3^
CFT073 c1501::*tet*A ∆c1500	8.7 × 10^3^	2.17 × 10^3^	5.37 × 10^3^	6.87 × 10^3^
Other helper phages				
C600 λ EcCICFT073 c1499::*cat*	2.2 × 10^3^	1.3 × 10^3^	1.1 × 10^3^	1 × 10^3^
594 80 EcCICFT073 c1499::*cat*	1.33 × 10^6^	2.68 × 10^6^	8 × 10^5^	1.02 × 10^6^
594 80 Δ*ter*S EcCICFT073 c1499::*cat*	< 1	< 1	< 1	< 1
CFT073 Prophage mutants^b^				
CFT073 c1501::*tet*A ∆ø1	1.01 × 10^4^			
CFT073 c1501::*tet*A ∆ø2	5.19 × 10^3^			
CFT073 c1501::*tet*A ∆ø4	< 1			
CFT073 c1501::*tet*A ∆ø5	3.90 × 10^3^			
CFT073 c1499::*cat* ∆ø4	< 1			

^a^Transductants/ml of lysate, using *E. coli* C600, WG5, 594 or DH5α (*rec*A mutant) as recipient strains. The means of results from three independent experiments are presented. Variation was within ± 5% in all cases

^b^Phages deleted in strain *E. coli* CFT073

We next tested for MC induction and PICI transfer with the non-lysogenic C600 derivative carrying the EcCICFT073::*tet*A element, and failed to detect amplification, lysis, or transfer, confirming that the EcCICFT073 transfer depends on a helper phage. Finally, we identified the EcCICFT073 helper phage. The CFT073 genome contains at least 5 prophage-like elements [28], of which #3 is actually EcCICFT073 and is listed as O-island 51. We deleted each of the other 4 and found that deletion of #4 eliminated detectable EcCICFT073::*tet*A induction and transfer (Fig. 3; Table 1), whereas none of the other prophage deletions had any effect.

### Functionality of the EcCICFT073 genes

Having established that the EcCICFT073 island can be phage-induced, we looked in more detail at the EcCICFT073-encoded genes to confirm that these ORFs encode proteins functional in the various aspects of the PICI life cycle as previously defined for the SaPIs. There are 5 key features of the PICI life cycle: (i) excision, which may be spontaneous, (ii) replication, (iii) specific packaging, (iv) interference, and (v) induction. Of these, induction depends on the helper phage and in the GP PICIs controls all of the aforementioned features.

#### Integration

To test for *int* (excision/circularization/integration) function, we initially performed PCR analysis with inward- and outward-directed primers as shown in Supplementary Fig. 4. Amplicons were obtained indicative of spontaneous excision and circularization, suggesting *int* functionality. To clearly prove this, we cloned the *int-att*_PI_ segment into the thermosensitive pMAK700 plasmid and tested it for integration in DH5α (*rec*A mutant) by overnight growth followed by plating on selective agar at the restrictive temperature. As controls, we generated pMAK700 derivative plasmid mutants in the *int* gene or in the *att*_PI_ site. We obtained colonies only from plasmids carrying an intact *int-att*_PI_ complex, confirming *int* functionality.

#### Replication

The previous results suggested that the GN PICIs encode a functional replicon. We have shown previously that the Pri-Rep-*ori* segment of SaPIbov1, SaPI1 or EfCIV583 can drive autonomous replication of a plasmid in different GP bacteria [3, 10, 22]. Since the putative PICIs seem to have the same replicon organization (Fig. 2a), we cloned the replication region of EcCICFT073, including the *rep* gene and the putative *ori* site, into the thermo-sensitive plasmid pKO3-blue [29], with *rep* under the control of an arabinose-inducible promoter. We also constructed plasmids carrying mutations in the *rep* gene or in the *ori* site. These plasmids were transformed into *E. coli* DH5α, and 5 × 10^5^ cells were plated on selective agar and incubated either at the permissive (30 °C) or the restrictive temperature (44 °C). Strains carrying the putative PICI *rep-ori* replicon generated colonies in *E. coli* DH5α (Fig. 2b), while the strains carrying the Δ*rep* mutant plasmid did not. Note that at 44 °C, the number of colonies obtained was reduced compared to 30 °C, suggesting defects on plasmid segregation. Since the deletion of the *ori* significantly reduced the number of colonies obtained compared to the wt plasmid, these results confirm that the GN PICIs encode a functional replication module.

#### Packaging

As with the phages, there are two strategies by which the SaPIs can be packaged. Some SaPIs that use the headful mechanism encode a homolog of the phage terminase small subunit (TerS_S_) that directs the preferential packaging of the SaPIs [7]. The TerS_S_ has the same function as the phage enzyme (TerS_P_), recognizing a specific (*pac*) site on its cognate genome to initiate packaging. Other SaPIs carry the helper phage *cos* sequence in the SaPI genome, which ensures SaPI packaging depending on the helper *cos* phage machinery [14]. The λ *cos* sequence contains three regions required to interact with the packaging machinery: *cosQ*, *cosN*, and *cosB*. *cosQ* is required for the termination of chromosome packaging. *cosN* is the site at which terminase cuts the DNA. Initiation of DNA packaging requires *cosN* and the adjacent *cosB* site. *cos*B consists of three binding sites, R3, R2, and R1, for λ TerS [30] (Fig. S5). EcCICFT073 does not encode a TerS homolog, suggesting that in order to be efficiently packaged EcCICFT073 probably uses the *cos* packaging strategy. As shown in Fig S5, EcCICFT073 carries two *cosN* sequences that are identical to those present in its inducing phage 4 (Fig. S5). Remarkably, these EcCICFT073 *cos*N sequences resemble that present in the archetypical *E. coli* λ and ϕ80 phages, used during decades as models in phage studies. However, we were unable to identify the phage *cos*B region in the EcCICFT073 element (Fig. S5), questioning if the island carries a bona fide *cos* sequence. To test the EcCICFT073 and phage *cos* sites for function, we introduced independently the λ, ϕ80, phage 4, and each of the two EcCICFT073 *cos* sites (containing the putative *cos*Q, *cos*N and *cos*B) into plasmid pET28a, which was not transferrable by λ, and found that the cloned *cos* sites enabled transfer of the plasmids by phages λ and ϕ80 (Fig. S5). Consistent with the presence of a completely different *cos*B sequence, transfer of the plasmids carrying the EcCICFT073 *cos* sites was reduced compared to that observed with the plasmids carrying the cognate phage *cos* sequences (Fig. S5).

The results described above raised the exciting possibility that phages λ and ϕ80 could work as helpers for EcCICFT073. To test that, and assuming EcCICFT073 is transferred using a *cos* mechanism, we generated one additional version of the PICI in which the antibiotic resistant marker did not change the size of the island. Note that in the EcCICFT073 *tet*A island the size of the element was increased by the insertion of the cassette in the middle of the island (Fig. S3). In the new variant, the *cat* marker replaced gene EcCICFT073 c1499 (Fig. S3), for which we have not been able to find a phenotype. The new island, generated in strain CFT073, was also introduced in the CFT073 mutant in the helper phage 4 and in the λ and ϕ80 lysogens. The different strains were SOS (MC) induced, and the transfer of the EcCICFT073 *cat* island analyzed. The analysis of the lysates obtained from the different CFT073 derivative strains revealed that transfer of EcCICFT073 *cat* by helper phage 4 was higher than that previously observed for the EcCICFT073 *tet*A island (Table 1), supporting the idea that EcCICFT073 uses a *cos* mechanism for packaging. Remarkably, phages λ and 80 transferred the EcCICFT073 *cat* at frequencies, for phage 80, significantly higher than those observed with the helper phage 4 (Table 1).

**Fig. 4:**
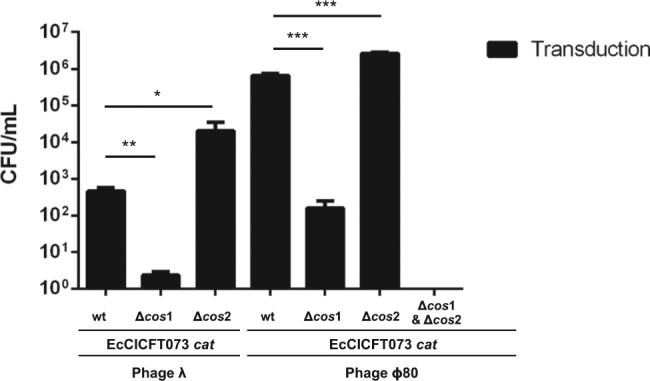
Functionality of the EcCICFT073 *cos* sites. EcCICFT073 *cat* wt, or its derivatives carrying mutations in the *cos*1 or/and *cos*2 sites, were introduced in the lysogenic strains for phages λ or ϕ80. The different strains were MC induced (2 μg/ml) and the transfer of the island quantified. A *t-test* was performed to compare the wt against the different *cos* mutants. Adjusted *p* values were as follows: Phage Lambda EcCICFT073 ∆*cos*1 = 0.002^**^, Phage Lambda EcCICFT073∆*cos*2 = 0.0266^*^, Phage 80 EcCICFT073 ∆*cos*1 = 0.003^***^, Phage ϕ80 EcCICFT073 ∆*cos*2 = 0.0002^***^

We next generated a set of mutant strains in which either one of each or both *cos* sites present in EcCICFT073 were deleted. Deletion of the EcCICFT073 *cos*1 site significantly reduced transfer of the element by phages λ and ϕ80, while deletion of the EcCICFT073 *cos*2 site slightly increased phage ϕ80-mediated transfer of the island but significantly increased transfer mediated by phage λ; deletion of both *cos* sites eliminated phage-mediated transfer of the island, confirming the identity of these sequences as *cos* sites and that both *cos* sites are functional (Fig. 4). This situation mirrors SaPIbov4 [31], which also has two putative *cos* site in its sequence.

Finally, we generated a phage 80 *ter*S mutant, and tested for both phage and EcCICFT073 transfer (Table 1). Deletion of the phage-encoded *ter*S gene abolished packaging and transfer of both elements, confirming that EcCICFT073 hijacks the phage proteins for transfer.

#### Interference

**Fig. 5:**
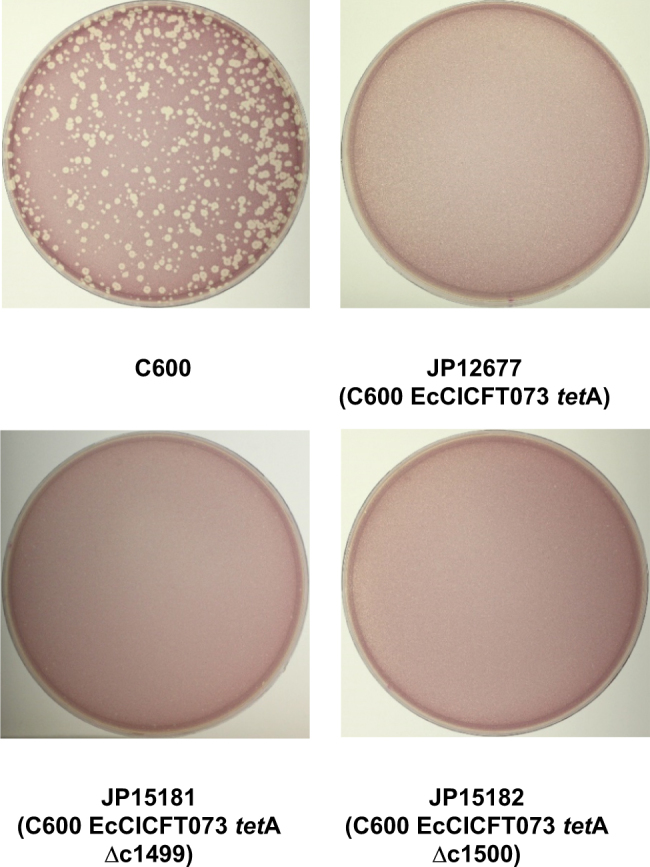
EcCICFT073 interferes with λ reproduction. *E. coli* strains C600, JP12677 (C600 EcCICFT073 *tet*A-positive), JP15181 (C600 EcCICFT073 *tet*A-positive ∆c1499) or JP15182 (C600 EcCICFT073 *tet*A-positive ∆c1500) were infected with phage λ (∼500 pfu per plate), plated on phage bottom agar, and incubated for 24 h at 37 °C. Plates were stained with 0.1% TTC in LB and photographed

EcCICFT073 severely interferes with phage λ reproduction (Fig. 5). A conserved mechanism of phage interference in many PICIs is the production of PICI-sized particles that are too small to package an intact phage genome [27, 32]. To test this possibility, we MC-induced the lysogenic strain carrying phage λ and EcCICFT073, the virions were pelleted and the extracted DNA was analyzed. All of the packaged DNA was phage-sized, ruling out the possibility that the observed interference was generated by capsid size redirection.

Next we searched for putative genes in the EcCICFT073 element that could be involved in this interference. EcCICFT073 encodes a putative capsid protein (ORF c1499; AAN79968) and a head decoration protein (ORF c1500; AAN79969.1). Since in SaPIbov5 the capsid protein homolog (Ccm) blocks phage reproduction by generating SaPI-sized capsids [8], we analyzed whether these proteins could be involved in the observed interference using an alternative mechanism. To do that, we deleted genes c1499 or c1500 in the EcCICFT073 *tet*A element present in the non-lysogenic strain C600, and tested the ability of λ to generate plaques in these strains. None of the mutants allowed normal phage reproduction (Fig. 5), suggesting that other EcCICFT073 genes are responsible for the observed interference. Moreover, deletion of the c1499 or c1500 genes in the CFT073 strain did not affect the transfer of the island by the resident prophages (Table 1).

#### Induction

**Fig. 6:**
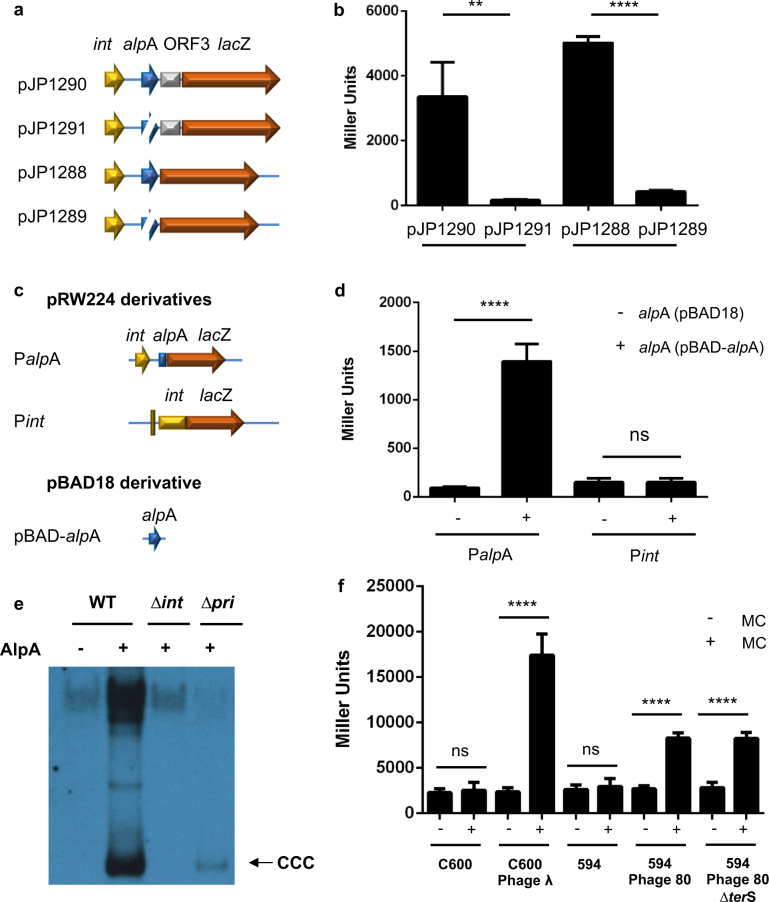
Characterization of the EcCICFT073 *alp*A gene. **a**–**c** Schematic representation of the different *bla*Z transcriptional fusions generated. The relevant genes are shown. **b**–**d** Miller assay performed using the plasmids represented in (**a**–**c**). Results (average ± s.d.) of three independent assays are shown. A 2-way ANOVA with Tukey’s multiple comparisons test was performed to compare mean differences within rows. Adjusted *p* values were as follows: pJP1290 vs pJP1291 = 0. 0011^**^, pJP1288 vs pJP1289 = 0.0001^***^, P*alp*A  < 0.0001^****^, P*int*  > 0.9999 ^ns^. *ns*, not significant. **e** EcCICFT073 excision and replication after expression of the cloned *alp*A gene. A non-lysogenic derivative of strain C600 carrying EcCICFT073 was complemented with plasmid pBAD18 (empty plasmid) or plasmid pJP2037, which carries *alp*A under the control of the *P*_BAD_ promoter. As controls, non-lysogenic derivatives of strain C600 carrying EcCICFT073 ∆*int* or EcCICFT073 ∆*pri* were complemented with plasmid pJP2037. One milliliter of each culture (optical density (OD)_600nm_ = 0.3) was collected 2 h after treatment with 0.02% arabinose and used to prepare standard minilysates, which were resolved on a 0.7% agarose gel, Southern blotted and probed for EcCICFT073 DNA. In these experiments, because no helper phage is present, the excised EcCICFT073 DNA appears as covalently closed circular molecules (CCC). **f** Helper phages activate *alp*A transcription. Different lysogenic and non-lysogenic strains, containing plasmid pJP1290, were MC-induced and assayed for β-galactosidase activity. Results (average ± s.d.) of three independent assays are shown. Strains: C600 (non-lysogenic), JP10400 (C600 lysogenic for phage λ), 594 (non-lysogenic), JP12507 (594 lysogenic for phage 80) and JP15151 (594 lysogenic for phage 80 ∆*ter*S). A 2-way ANOVA with Tukey’s multiple comparisons test was performed to compare mean differences within rows. Adjusted *p* values were as follows; C600 > 0.9999 ^ns^, Phage Lambda < 0.0001^****^, 594 > 0.9999 ^ns^, Phage 80 < 0.0001^****^, Phage 80 ∆*ter*S < 0.0001^****^. *ns*, not significant

The main difference between the GP and GN PICIs lies in the regulatory region (Fig. 1 and S1). While the GP elements encode a master repressor (called Stl in the SaPIs or generically Rpr in the other GP elements) [10, 22], the existence of a global repressor in the GN elements is unclear. One attractive candidate is AlpA, homologs of this protein being encoded by all the *E. coli* PICIs (Fig. 1 and S1). AlpA is predicted to act as a DNA-binding transcriptional regulator, and previous work suggested that it controls the expression of *int* in a P4-like phage [33, 34]. We hypothesized that AlpA could act as the main repressor of the PICIs, blocking expression of the PICI genes. To test this, we initially generated different transcriptional fusions in which the *lac*Z reporter was fused either to EcCICFT073 ORF3 or was located after the stop codon of *alp*A (see scheme in Fig. 6a). Expression of the reporter was measured in the presence or absence of *alp*A. Contrary to our hypothesis, AlpA is not a repressor but seems to be an activator of the EcCICFT073 genes. To confirm this, and also to know if AlpA controls *int* expression, we fused the promoter regions of *alp*A (plasmid P*alp*A) or *int* (plasmid P*int*) to *lac*Z, using plasmid pRW224 (see scheme in Fig. 6c). Simultaneously, the *alp*A gene was cloned in plasmid pBAD18 under the control of the *P*_BAD_ promoter, inducible by arabinose, generating plasmid pBAD-*alp*A. Plasmids P*alp*A or P*int* were introduced into the strains carrying plasmids pBAD-*alp*A or pBAD18, and the expression of the different promoter regions monitored in presence or absence of arabinose. As expected, AlpA activates its own expression. Contrary to previous results [33, 34], AlpA does not seem to control *int* expression in EcCICFT073.

Our results suggested that induction of the GN PICIs requires the expression of the AlpA activator. In support of this, inactivation of *alp*A significantly diminished induction and transfer of the EcCICF073 island present in the CFT073 strain (Fig. 3, Table 1). Moreover, over-expression of AlpA in the non-lysogenic *E. coli* C600 carrying the EcCICFT073 island resulted in induction and uncontrolled replication of the EcCICF073 element (Fig. 6e). In support of the previous results, this uncontrolled replication requires the expression of the EcCICFT073 encoded *pri* and *int* genes (Fig. 6e). Note that in this case, and since the strain is not lysogenic, just the closed circular (CCC) and the concatemeric (bulk) forms are observed because the element is not packaged. As occurred with the SaPIs [10], the uncontrolled replication of the EcCICFT073 affected both the growth curve and the viability of the *E. coli* cells (Fig. S6).

Finally, we introduced the pJP1290 plasmid, which measures expression of *alp*A, in strains C600 and 594 (non-lysogenic) and the C600 and 594 derivatives lysogenic for phages λ and ϕ80, respectively. Since AlpA is absolutely required for the transfer of the island, we hypothesized that the helper phages would induce expression of *alp*A. To test this, the different strains were MC-induced and the expression of the *bla*Z reporter monitored. As expected, *alp*A expression does not depend on the SOS response but on the presence of a helper phage. Thus, *alp*A expression was increased in the induced lysogenic strains, compared to the induced non-lysogenic C600 and 594 strains (Fig. 6f). Note that *alp*A expression was also increased in the lysogenic strain carrying the phage ϕ80 mutant in the *ter*S gene. Since this phage mutant can’t mobilize the island, this strain was used to propose that the EcCICFT073 element hijacks the phage machinery for packaging. However, another possibility could be that *ter*S was the EcCICFT073 inducer. The fact that this mutant induces *alp*A expression rule out this possibility. The identification of the phage-encoded EcCICF073 inducer is now under study.

### PmCI172

**Fig. 7:**
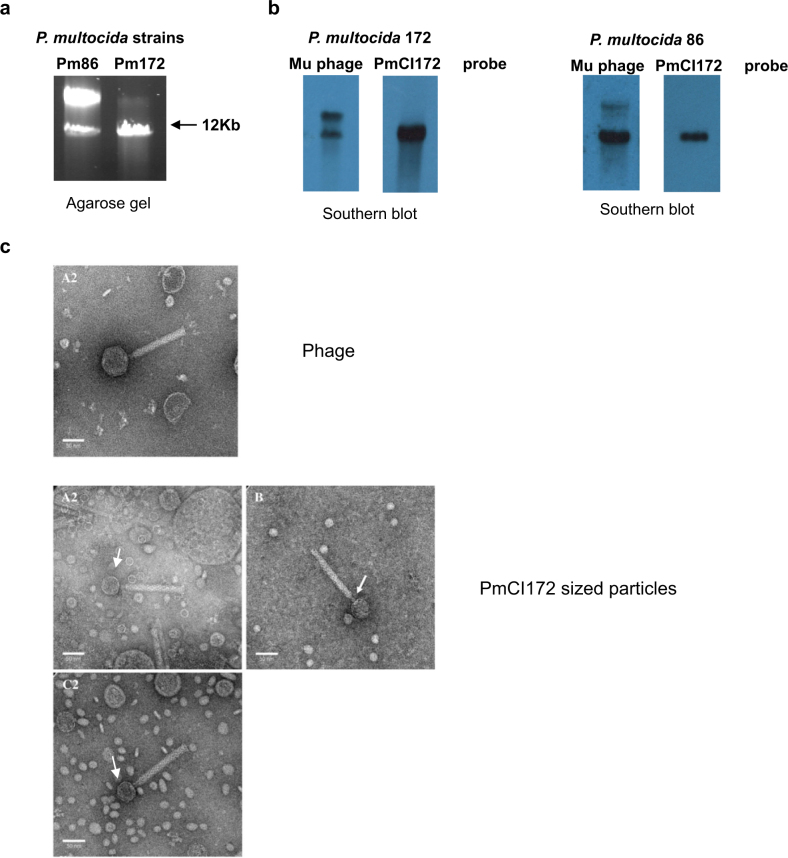
Induction of PmCI172 and PmCI86. **a** DNA extracted from a phage lysates of MC-treated cultures of *P. multocida* strains 172 or 86. **b** Southern blot of the DNA shown in panel (**a**), using a phage- or PmCI-specific probe. **c** Electron microscopy analysis of the MC-induced *P. multocida* 172 lysate. Several different fields are shown, containing normal Mu phage particles and PmCI172 particles (arrows), which have smaller heads. Scale bars are 50 nm

In a complementary study aimed at characterizing prophages present in the animal pathogen *P. multocida*, we MC-induced different lysogenic strains, the particles were pelleted and the extracted DNA was separated. As shown in Fig. 7, two bands, one of phage monomer size and the other of PICI monomer size, appeared in the analysis of strains Pm86 and Pm172. This is the classical pattern observed when a strain containing a PICI-helper phage pair is MC-induced, suggesting that *P. multocida* contains *bona fide* PICI elements, which are packaged in small capsids as are many of the analyzed PICIs [8, 27, 32, 35]. To confirm that this was the case, we initially sequenced both the *P. multocida* strains as well as the DNA obtained from the induced lysates. As expected, we identified a PICI element in both strains, which incidentally were identical (Fig. 1; Table S1). While two phages were present in strain Pm86 (Fig. S7), strain Pm172 only contained one (Fig. S7). The latter phage was also present in strain Pm86, suggesting this is the helper phage for the PmCI172 and PmCI86 PICIs. This phage belongs to the Mu family of phages, demonstrating for the first time a Mu-like phage, belonging to the *Myoviridae* family, as helper phage for a PICI element.

Next, we confirmed that the PmCI172- and Pm86-Mu phage are a bona fide PICI-helper phage interaction by performing a Southern blot analysis of the packaged DNA observed in Fig. 7, using PmCI172- and phage Mu-specific probes. Southern blot analyses revealed that the Mu phage probe hybridized both with the PICI-sized (small) and the phage-sized (large) DNA bands (Fig. 7), suggesting that the phage DNA can be packaged in the PmCI-sized particles. Packaging of a significant proportion of phage DNA in the small particles generates defective phages, confirming that PmCI172 and PmCI86 PICIs interfere with phage reproduction using a very well conserved strategy, production of PICI-sized capsids, which is a key feature of the previously analyzed GP PICI elements [8, 27, 32, 35].

Finally, to confirm the existence of the PICI-sized capsids, we subjected the virions to electron microscopy (EM). The phage-sized particles had the characteristic size and shape of this class of bacteriophages: hexagonal capsids of approximately 59 to 63 nm length and 51 to 56 nm width and long contractile tails, which are approximately 150 nm long and 15 to 16 nm wide. (Fig. 7c). As expected, the majority of the particles had small, hexagonal heads, about 38–39 nm length and 37 nm wide, attached to approximately 150 nm long and 15 nm wide tail (Fig. 7c). This result confirms that PmCI172 causes the formation of small capsids, consistent with its smaller genome size. As occurs with SaPIbov5, which encodes a capsid protein responsible of the production of the SaPI-sized small capsids [8], PmCI172 also encodes a major capsid protein homolog (Fig.1), which could be involved in this process. Unfortunately, the absence of tools to manipulate *P. multocida* has impaired us deciphering in detail the biology of the PmCI172 element.

## Discussion

In this report we demonstrate that PICIs, of which the staphylococcal SaPIs are a subset, are not just confined to GP bacteria but are widespread among other bacteria. Although all the GN PICIs identified in this study are in *Gammaproteobacteria*, and more specifically in members of the *Enterobacteriaeae* and *Pastuerellaceae*, the fact that similar elements can be found both in GP and GN bacteria confirms the universality of this novel family of MGEs. PICIs are phage satellites with a unique genomic organization which is easy to see in KEGG genome maps and sets them apart from other mobile genetic elements. They interact with certain “helper” bacteriophages in a unique and highly characteristic manner: in the GP cocci, the PICI encode an SOS-insensitive master repressor, Stl, that maintains it in a quiescent state and is counteracted by a specific phage protein, initiating the PICI life cycle [13, 22]. In the GN bacteria, it seems the PICIs use a completely different strategy coupling the phage and the PICI cycles. In this case, the GN PICIs encode AlpA, an activator of the PICI cycle whose expression depends on the helper phage. Why GP and GN bacteria use different strategies to be induced is a mystery. In both cases, however, PICI induction is followed by PICI replication, encapsidation of PICI DNA in infectious particles composed of phage proteins and finally high transfer of these elements.

Like their GP counterparts, GN PICIs also severely interfere with helper phage reproduction, this probably being one of the *raisons d’être* of these elements. The PICI genomes are usually about 1/3 the size of their helper phage genomes and among the GP PICIs, many encode proteins that redirect the helper phage to form capsids 1/3 the size of the phage capsid [7, 8, 27]. This strategy is also used by the GN PICIs. Although we have not identified the genes responsible for this process, none of the proteins encoded by the PmCI172 element resemble previously characterized proteins, suggesting that capsid size redirection is a convergent evolutionary process characteristic of all the PICI elements, which in turn defines the size of these elements.

As occurs with GP PICIs, GN PICIs have an important role in virulence and several of the newly identified PICIs present in GN bacteria contain recognizable accessory genes. These are listed in Table S1. The identities of many of these genes are based on annotations; just a few of them have been tested experimentally. Several elements, including islands harbored by variants of the notorious *E. coli* O157:H7 encode RgdR, which regulates expression of the type III secretion (T3S) system, encoded by the locus of enterocyte effacement (LEE) of the *E. coli* pathogenicity island, PAI-I. Deletion of this island in *E. coli* O157 strain TUV93-0 resulted in a reduction in LEE expression and T3S [36]. This deletion also reduced the capacity of the bacteria to attach to epithelial cells and reduced the shedding of *E. coli* O157 by sheep. RgdR also controls the expression of other genes, including those involved in motility, suggesting a global role in *E. coli* gene expression [36]. It is especially relevant for this report that EcCICFT073, and the related EcCI042, two of the *rgd*R-containing elements in *E. coli*, correspond to O-island 51, a 15 kb O157:H7 island annotated, as usual, as a defective prophage [36]. In addition to RgdR, which is encoded by all the analyzed *E. coli* PICIs, some *E. coli* PICIs encode allelic variants of PerC. Remarkably, some but not all the PICI-coded PerC homologs also activate expression of the LEE pathogenicity island [37].

We suggest that all of the PICIs in the GN species are co-ancestral and represent a separate but very similar lineage, since their transcriptional organization is mirrored in rare phages from GN organisms. At the same time, the unique organization of the replication origin is the same for both the GP and GN lineages, which is consistent with co-ancestry. Although this organization was not a criterion for the identification of the PICIs, it is shared by all those identified in this study and by no other known bacterial replicon. Coancestry could mean that the divergent evolution of PICIs and host chromosomes has simply been parallel, or that the PICIs have been horizontally transferred and have retained greater similarities than their host bacteria. Current studies in our labs are focused on deciphering these interesting evolutionary questions. Our overall view, therefore, is that the PICIs represent two or more families of unique mobile elements whose biological features have been strongly selected during long-term evolution, so that they represent coherent clades that have diverged from and evolved in parallel with temperate phages (or proto-phages) and have spread widely among diverse bacterial genera. If this spread has been via helper phage induction and packaging, then it is likely that spread would have occurred between species that share phage adsorption receptors, leading to a chain of island-hopping (or rather host-hopping) events that could, in principle, be tracked by comparing PICI gene similarities as a function of the relatedness of host bacterial phage receptors.

The P2-P4 system in *E. coli* represents a theoretical precedent for phage satellites, in which P4 utilizes P2 virion proteins to form small capsids into which its small genome is packaged [38, 39]. PICIs and P4 share some features in common: (i) phage-mediated activation of their life cycles, which in the case of P4 depends on the P2 Cox protein [40], while for the GN PICIs the activator remains to be identified; (ii) autonomous replication, which in the case of P4 depends on the α protein [41] and its cognate *ori* site (named *ccr* [42, 43]); (iii) carriage of the helper phage *cos* sequence in their genomes, which allows these elements to parasite the packaging machinery of their cognate helper phages; (iv) interference with phage reproduction, a process that in P4 depends on Sid, a protein that redirects capsid formation generating P4-sized particles [44].

However, and in spite of these similarities, P4 and the PICIs present in *E. coli* show important differences supporting the idea that they have evolved independently: (i) P4 and PICIs are fundamentally different both in genomic structure (see Fig. S8) and in gene content. As occurred with the PICIs, the KEGG analysis revealed that the P4 genes are exclusive of this family of elements (Table S5). Importantly, only three PICI-coded proteins (AlpA, Pri-Rep and Int) showed distant homology with the P4-coded proteins, corresponding to proteins that have similar roles in both elements. We have identified 4 allelic variants of AlpA in the *E. coli* PICIs (Fig. S9), all of them being remotely related to the AlpA-homolog (named Vis) encoded by P4 (Fig. S9). In fact, these proteins seem to have a different role in each of the elements: while Vis is required for P4 excision [45], AlpA controls induction of the EcCICFT073 cycle. In the case of the Pri-Rep proteins, a similar scenario is observed: 4 allelic variants are found in the PICI elements from *E. coli* (Fig. S10), which are different in sequence from the α protein of P4 (Fig. S10). The situation with the Int proteins is even more interesting. We have identified in *E. coli* 36 *att*_C_ sites for phages, 8 for PICIs and 4 for P4 elements (Fig. S2). All the *att*_C_ sites are exclusive to one type of element except one, which is shared by PICIs and P4. Importantly, and with the exception of the *int* gene, the P4 and PICI elements that integrate in the same *att*_C_ conserved their own features and are completely different both in sequence and structure (Fig. S11). (ii) PICIs do not have a plasmid state, nor encode genes to control plasmid copy number or plasmid segregation. (iii) PICIs play an important role in virulence, with many *E. coli* elements encoding two activators of the LEE pathogenicity island. (iv) P2 and P4 mutually cross induce one another through back-and-forth transcriptional activation. Indeed, P4 forms plaques on a P2 lysogen, but these are P2 plaques resulting from P4 induction of P2, whereas we have not been able to see phage induction mediated by an incoming GN PICI. (v) In *E. coli*, P4 and PICI elements seem to parasitize two completely different families of helper phages: *Myoviridae* and *Siphoviridae*, respectively. Taken together, all these similarities and divergences highlight the idea that although these elements have evolved independently, their parallel *modus vivendi* has been strongly selected in nature.

A similar scenario occurs with a family of pathogenicity islands recently identified in *Vibrio cholerae*. These elements, denominated PLEs for phage-inducible chromosomal island-like elements, are widespread genomic islands that share some features with the classical PICIs, including mobilization by helper phages and severe interference with phage reproduction [46, 47]. In contrast to the GN PICIs, PLEs are larger in size (18–19 kb) and have both different genetic organization and gene content. Thus, although they encode an integrase, they do not encode identifiable regulatory, replication or packaging modules, even though infection by a helper phage leads to PLE excision, replication and packaging [46]. Whether these functions depend on the helper phage machinery, or by contrast are provided by still-unidentified PLE genes remains to be determined. Further studies are required to understand the origins of, and relationships among, these subcellular elements and their cognate phages.

Although *cos* sites were long ago cloned into *E. coli* plasmids, generating cosmids, which are widely used as cloning vectors owing to their efficient transfer by phage λ, our results represent the first demonstration of naturally-occurring phage λ-mediated transfer. Since SaPIbov5 is also transferred by helper *cos* phages, our results with the PICIs represent a paradigm shift involving *cos* phages in gene transfer. The PICIs are a special case because they undergo phage-like replication, generating concatemeric post-replicative DNA, which would have *cos* sites spaced one genome apart. However, EcCICFT073 lacks the capsid morphogenesis genes carried by most SaPIs and is therefore packaged in full-sized phage capsids, which are designed to accommodate the 45 kb phage genomes. This would mean that the phage terminase skips two successive *cos* sites during packaging; the mechanism by which this occurs remains to be determined, although it is likely that the expansion generated once the capsid is fulfilled controls this phenomenon.

Bacteria are successful as commensal organisms or pathogens in part because they adapt rapidly to selective pressures. MGEs play a central role in this adaptation process and are a means to transfer genetic information among and within bacterial species. Here, and as is the case with other classical MGEs like plasmids, transposon or phages, we demonstrate that the PICIs are widespread in nature. These findings represent the discovery of a universal agency of horizontal dissemination of important accessory genes in the bacterial universe.

## Materials and methods

### Identification of PICI candidates

The following criteria were initially used to identify putative PICIs in the GN genomes: (i) well-conserved gene organization, including an integrase, a replicating module and a master transcriptional regulator; (ii) unique attachment (*att*) sites that are never occupied by prophages; (iii) the absence of phage structural and lytic genes; and (iv) size typically around 12–15 kb. After this initial screening, the analysis of orthologies points to elements that might correspond to PICIs. Examination of the corresponding KEGG genome maps (http://www.genome.jp/kegg; release May 1 2016) was used to confirm the identifications. In these analyses we examined the *E. coli* and the *P. multocida* genomes that have been coded for KEGG because the KEGG genome maps enable PICI-like elements to be readily identified.

### Bacterial strains and growth conditions

Bacterial strains used in these studies are listed in Supplementary Table S7. *E. coli* strains were grown at 37 °C or 30 °C overnight on Luria-Bertani agar and on Luria-Bertani broth with shaking (180 r.p.m.). *P. multocida* strains were grown at 37 °C overnight on Brain-Heart-Infufion agar and on Brain-Heart-Infufion broth with shaking (250 r.p.m.). Ampicillin (100 µg ml^−1^), Kanamycin (30 µg ml^−1^), Chloramphenicol (20 µg ml^−1^) or Tetracycline (20 µg ml^−1^), from Sigma, were added when appropriate. Phage and PICI analyses were performed, as described previously [11–13].

### DNA methods

Gene insertion was performed as described [48]. The tetracycline resistance marker (*tet*A) or chloramphenicol resistance marker (*cat*) was amplified by PCR with primers listed in Supplementary Table S6. The resulting PCR products were used to transform the recipient strain harboring plasmid pKD46 [48], which expresses Lambda Red recombinase. PCR was performed to verify the insertion of markers.

### Plasmid construction

Plasmids used in this study were constructed by cloning PCR products amplified with oligonucleotide primers purchased from Invitrogen. Clones were sequenced by Eurofins. Plasmids and primers are listed in Supplementary Table S8 and Table S6.

### Southern blot

Following plasmid (arabinose) or phage (MC) induction, samples were taken and pelleted at the indicated times. Samples were re-suspended in 50 μl lysis buffer (47.5 μl TES-Sucrose and 2.5 μl lysozyme [10 μg/ml]) and incubated at 37 °C for 1 h. Subsequently, 55 μl of SDS 2% proteinase K buffer (47.25 μl H2O, 5.25 μl SDS 20%, 2.5 μl proteinase K [20 mg/ml]) was added and incubated at 55 °C for 30 min. Samples were vortexed with 10 μl of 10 × loading dye. Cycles of incubation in dry ice with ethanol and at 65 °C were performed. Chromosomal DNA was separated by agarose gel electrophoresis. Samples were run on 0.7% agarose gel at 30 V overnight. Nylon membranes (Hybond-N 0.45 mm pore size filters; Amersham Life Science) were used for the transfer of DNA. DNA was detected using a DIG-labeled probe and anti-DIG antibody.

### β-galactosidase assays

Strains were grown at 37 °C in LB medium containing the appropriate antibiotics. Following plasmid (arabinose) or phage induction (MC), samples were taken and pelleted at the indicated times. The Miller method [49] was used to measure β-galactosidase levels. The average of at least three independent experiments is shown in Miller units.

### Electron microscopy (*P. multocida*)

Ten milliliters of filtered *P. multocida* Pm172 phage lysate were centrifuged at 40,000 × *g* for 90 min at 4 °C. The supernatant was carefully removed and the pellet was re-suspended gently with 0.5 ml of 0.1 M ammonium acetate (pH 7.3). The suspension was stored overnight at 4 °C for negative staining and visualization by TEM. Three hundred-mesh carbon-coated nickel grids were dropped onto 50–100 µL of phage suspension. The grids were allowed to adsorb phage suspension for 2 min. The grids were washed 3 times with dH_2_O for 10″, excess fluid was removed using Whatman filter paper and the grids were negatively stained with 2% ammonium molybdate for 30″. Excess staining solution was removed with Whatman filter paper. The grids were allowed to dry at room temperature for ~15–20 min. The samples were examined by TEM (FEI Tecnai TF20) at 200 kV using Gatan Microscopy Suite Software.

## Electronic supplementary material

Supplementary tables

Supplemtary figures

## References

[1] Mir-Sanchis I, Martínez-Rubio R, Martí M, Chen J, Lasa I, Novick RP (2012). Control of *Staphylococcus aureus* pathogenicity island excision. Mol Microbiol.

[2] Ubeda C, Tormo MA, Cucarella C, Trotonda P, Foster TJ, Lasa I (2003). Sip, an integrase protein with excision, circularization and integration activities, defines a new family of mobile *Staphylococcus aureus* pathogenicity islands. Mol Microbiol.

[3] Ubeda C, Barry P, Penadés JR, Novick RP (2007). A pathogenicity island replicon in *Staphylococcus aureus* replicates as an unstable plasmid. Proc Natl Acad Sci USA.

[4] Quiles-Puchalt N, Martínez-Rubio R, Ram G, Lasa I, Penadés JR (2014). Unravelling bacteriophage ϕ11 requirements for packaging and transfer of mobile genetic elements in Staphylococcus aureus. Mol Microbiol.

[5] Tallent SM, Christie GE (2007). Transducing particles of Staphylococcus aureus pathogenicity island SaPI1 are comprised of helper phage-encoded proteins. J Bacteriol.

[6] Tormo MA, Ferrer MD, Maiques E, Ubeda C, Selva L, Lasa I (2008). *Staphylococcus aureus* pathogenicity island DNA is packaged in particles composed of phage proteins. J Bacteriol.

[7] Ubeda C, Maiques E, Tormo MA, Campoy S, Lasa I, Barbé J (2007). SaPI operon I is required for SaPI packaging and is controlled by LexA. Mol Microbiol.

[8] Carpena N, Manning KA, Dokland T, Marina A, Penadés JR (2016). Convergent evolution of pathogenicity islands in helper cos phage interference. Philos Trans R Soc Lond B Biol Sci.

[9] Penadés JR, Christie GE (2015). The phage-inducible chromosomal islands: a family of highly evolved molecular parasites. Annu Rev Virol.

[10] Ubeda C, Maiques E, Barry P, Matthews A, Tormo MA, Lasa I (2008). SaPI mutations affecting replication and transfer and enabling autonomous replication in the absence of helper phage. Mol Microbiol.

[11] Bowring J, Neamah MM, Donderis J, Mir-Sanchis I, Alite C, Ciges-Tomas JR (2017). Pirating conserved phage mechanisms promotes promiscuous staphylococcal pathogenicity island transfer. eLife.

[12] Tormo-Más MAacute, Donderis J, García-Caballer M, Alt A, Mir-Sanchis I, Marina A (2013). Phage dUTPases control transfer of virulence genes by a proto-oncogenic G protein-like mechanism. Mol Cell.

[13] Tormo-Más MAacute, Mir I, Shrestha A, Tallent SM, Campoy S, Lasa I (2010). Moonlighting bacteriophage proteins derepress staphylococcal pathogenicity islands. Nature.

[14] Quiles-Puchalt N, Carpena N, Alonso JC, Novick RP, Marina A, Penadés JR (2014). Staphylococcal pathogenicity island DNA packaging system involving cos-site packaging and phage-encoded HNH endonucleases. Proc Natl Acad Sci USA.

[15] Maiques E, Ubeda C, Tormo MA, Ferrer MD, Lasa I, Novick RP (2007). Role of staphylococcal phage and SaPI integrase in intra- and interspecies SaPI transfer. J Bacteriol.

[16] Chen J, Carpena N, Quiles-Puchalt N, Ram G, Novick RP, Penadés JR (2015). Intra- and inter-generic transfer of pathogenicity island-encoded virulence genes by cos phages. ISME J.

[17] Chen J, Novick RP (2009). Phage-mediated intergeneric transfer of toxin genes. Science.

[18] Ram G, Chen J, Kumar K, Ross HF, Ubeda C, Damle PK (2012). Staphylococcal pathogenicity island interference with helper phage reproduction is a paradigm of molecular parasitism. Proc Natl Acad Sci USA.

[19] Ram G, Chen J, Ross HF, Novick RP (2014). Precisely modulated pathogenicity island interference with late phage gene transcription. Proc Natl Acad Sci USA.

[20] Damle PK, Wall EA, Spilman MS, Dearborn AD, Ram G, Novick RP (2012). The roles of SaPI1 proteins gp7 (CpmA) and gp6 (CpmB) in capsid size determination and helper phage interference. Virology.

[21] Frígols B, Quiles-Puchalt N, Mir-Sanchis I, Donderis J, Elena SF, Buckling A (2015). Virus satellites drive viral evolution and ecology. PLoS Genet.

[22] Martínez-Rubio R, Quiles-Puchalt N, Martí M, Humphrey S, Ram G, Smyth D (2017). Phage-inducible islands in the Gram-positive cocci. ISME J.

[23] Novick RP, Christie GE, Penadés JR (2010). The phage-related chromosomal islands of Gram-positive bacteria. Nat Rev Microbiol.

[24] Novick RP, Ram G (2016). The floating (Pathogenicity) Island: a genomic dessert. Trends Genet.

[25] Asadulghani M, Ogura Y, Ooka T, Itoh T, Sawaguchi A, Iguchi A (2009). The defective prophage pool of *Escherichia coli* O157: prophage-prophage interactions potentiate horizontal transfer of virulence determinants. PLoS Pathog.

[26] Dearborn AD, Wall EA, Kizziah JL, Klenow L, Parker LK, Manning KA (2017). Competing scaffolding proteins determine capsid size during mobilization of *Staphylococcus aureus* pathogenicity islands. eLife.

[27] Matos RC, Lapaque N, Rigottier-Gois L, Debarbieux L, Meylheuc T, Gonzalez-Zorn B (2013). *Enterococcus faecalis* prophage dynamics and contributions to pathogenic traits. PLoS Genet.

[28] Welch RA, Burland V, Plunkett G, Redford P, Roesch P, Rasko D (2002). Extensive mosaic structure revealed by the complete genome sequence of uropathogenic *Escherichia coli*. Proc Natl Acad Sci USA.

[29] Solano C, García B, Latasa C, Toledo-Arana A, Zorraquino V, Valle J (2009). Genetic reductionist approach for dissecting individual roles of GGDEF proteins within the c-di-GMP signaling network in Salmonella. Proc Natl Acad Sci USA.

[30] Rao VB, Feiss M (2008). The bacteriophage DNA packaging motor. Annu Rev Genet.

[31] Viana D, Blanco J, Tormo-Más MAacute, Selva L, Guinane CM, Baselga R (2010). Adaptation of Staphylococcus aureus to ruminant and equine hosts involves SaPI-carried variants of von Willebrand factor-binding protein. Mol Microbiol.

[32] Ruzin A, Lindsay J, Novick RP (2001). Molecular genetics of SaPI1–a mobile pathogenicity island in *Staphylococcus aureus*. Mol Microbiol.

[33] Kirby JE, Trempy JE, Gottesman S (1994). Excision of a P4-like cryptic prophage leads to Alp protease expression in *Escherichia coli*. J Bacteriol.

[34] Trempy JE, Kirby JE, Gottesman S (1994). Alp suppression of Lon: dependence on the *slp*A gene. J Bacteriol.

[35] Ubeda C, Maiques E, Knecht E, Lasa I, Novick RP, Penadés JR (2005). Antibiotic-induced SOS response promotes horizontal dissemination of pathogenicity island-encoded virulence factors in staphylococci. Mol Microbiol.

[36] Flockhart AF, Tree JJ, Xu X, Karpiyevich M, McAteer SP, Rosenblum R (2012). Identification of a novel prophage regulator in *Escherichia coli* controlling the expression of type III secretion. Mol Microbiol.

[37] Porter ME, Mitchell P, Free A, Smith DGE, Gally DL (2005). The LEE1 promoters from both enteropathogenic and enterohemorrhagic *Escherichia coli* can be activated by PerC-like proteins from either organism. J Bacteriol.

[38] Lindqvist BH, Dehò G, Calendar R (1993). Mechanisms of genome propagation and helper exploitation by satellite phage P4. Microbiol Rev.

[39] Christie GE, Dokland T (2012). Pirates of the Caudovirales. Virology.

[40] Saha S, Haggård-Ljungquist E, Nordström K (1989). Activation of prophage P4 by the P2 Cox protein and the sites of action of the Cox protein on the two phage genomes. Proc Natl Acad Sci USA.

[41] Ziegelin G, Lanka E (1995). Bacteriophage P4 DNA replication. FEMS Microbiol Rev.

[42] Magnoni F, Sala C, Forti F, Dehò G, Ghisotti D (2006). DNA replication in phage P4: characterization of replicon II. Plasmid.

[43] Tocchetti A, Serina S, Terzano S, Dehò G, Ghisotti D (1998). Identification of two replicons in phage-plasmid P4. Virology.

[44] Agarwal M, Arthur M, Arbeit RD, Goldstein R (1990). Regulation of icosahedral virion capsid size by the in vivo activity of a cloned gene product. Proc Natl Acad Sci USA.

[45] Calì S, Spoldi E, Piazzolla D, Dodd IB, Forti F, Dehò G (2004). Bacteriophage P4 Vis protein is needed for prophage excision. Virology.

[46] O’Hara BJ, Barth ZK, McKitterick AC, Seed KD (2017). A highly specific phage defense system is a conserved feature of the Vibrio cholerae mobilome. PLoS Genet.

[47] Seed KD, Lazinski DW, Calderwood SB, Camilli A (2013). A bacteriophage encodes its own CRISPR/Cas adaptive response to evade host innate immunity. Nature.

[48] Datsenko KA, Wanner BL (2000). One-step inactivation of chromosomal genes in *Escherichia coli* K-12 using PCR products. Proc Natl Acad Sci USA.

[49] Miller JH. Experiments in molecular genetics. Cold Spring Harbor Laboratory Press, 1972.

