# Supplementary material for: Phage-inducible chromosomal islands are ubiquitous within the bacterial universe
Source: ISME J. 2018 Jun 6;12(9):2114–28. doi: 10.1038/s41396-018-0156-3 (PMC6092414; doi:10.1038/s41396-018-0156-3)
Supplement: Supplementary file 1 — Supplementary tables [file 41396_2018_156_MOESM1_ESM.pdf]

Table S1. PICIs in the Gram-negative (GN) bacteria: Genomes and characteristics.

PICI	Strain	Accession number (Genomic location)	Size (Kb)	*att* site core	*att* gene	Accessory genes
EcCICFT073	*Escherichia coli CFT073*	AE014075.1 (1377749-1389210)	11.4	GGACATATTT	*ycf*D-*pho*Q	*rdg*R; *per*C
EcCIRM12579	*Escherichia coli O55:H7 str. RM12579*	NC_017656.1 (1438155-1449376)	11.2	GGACATATTT	*ycf*D-*pho*Q	*rdg*R; *per*C
EcCI042	*Escherichia coli 042*	FN554766.1 (1832521-1843554)	11	ATAACAGCA	*tqs*A	*rdg*R; *per*C
EcCIK1516	*Escherichia coli O15:H18 str. K1516*	JHJE01000001.1 (141718-153137)	11.4	ATAACAGCA	*tqs*A	*rdg*R
EcCIATCC 25922	*Escherichia coli ATCC 25922*	CP009072.1 (106749-118031)	11.3	TTTTAGTTATACGTTTAGTTATACGG/ TTTTAGTTATACGTTTGGTTATACGG	*yej*M-*lux*R	*rdg*R
EcCIED1a.1	*Escherichia coli ED1a*	CU928162.2 (2568989-2580428)	11.4	TTTTAGTTATACGTTTAGTTATACGG/ TTTTAGTTATACGTTTGGTTATACGG	*yej*M-*lux*R	*rdg*R
EcCI11128	*Escherichia coli O111:H- str. 11128*	AP010960.1 (2855234-2864550)	9.3	TTTTAGTTATACGTTTAGTTATACGG	*yej*M-*lux*R	*rdg*R
EcCI11368.1	*Escherichia coli O26:H11 str. 11368*	AP010953.1 (3041660-3050231)	8.5	TTTTAGTTATACGTTTAGTTATACGG	*yej*M-*lux*R	*rdg*R
EcCIIHE3034	*Escherichia coli IHE3034*	NC_017628.1 (920272-930825)	10.5	ATGATAGTTAT	*csp*D-*clp*S	*rdg*R
EcCI18098	*Escherichia coli ST127 18098*		10.8	ATGATAGTTAT	*csp*D-*clp*S	*rdg*R
EcCIEC2733.1	*Escheichia coli genome assembly 1.EC2733.1*	CXXR01000010.1 (90531-101020)	10.5	ACATTATTAATATCGTGA	*lys*R-*smr*A	*rdg*R
EcCIDi14	*Escherichia coli clone D i14*	CP002212 (249047-259688)	10.6	CGGTGTAGTTAATGGTGTAGTTAATT	IS110 transposa se	*rdg*R
EcCIRM13514	*Escherichia coli O145 H28 str. RM13514*	CP006027 (4527455-4539396)	11.9	GAGCAGATTCAA	*yic*C-*din*D	*rdg*R
EcCI224	*Escherichia coli strain Ecol_224*	CP018948.1 (1961620-1972242)	10.6	AGAAATACCCGTAT	*frm*R-*thi*M	*rdg*R
PmCIHB03	*Pasteurella multocida subsp. multocida str. HB03*	CP003328.1 (1623899-1638673)	14.7	TTCGACTCCCATTCTC	*sel*A-*hsr*A	*abi*F
PmCIOH1905	*Pasteurella multocida OH1905*	CP004392.1 (1709089-1717107)	8	AGTCCTGTTGGG	*dct*Q-*yea*Q	
PmCI172	*Pasteurella multocida 172*	MH238469	7.8	AGTCCTGTTGGG	*dct*Q-*yea*Q	
PmCI82	*Pasteurella multocida 82*	MH238468	7.9	AGTCCTGTTGGG	*dct*Q-*yea*Q	
SdCI1012	*Shigella dysenteriae 1012*	AAMJ02000027.1 (22790-33855)	11	TTTTAGTTATACGTTTAGTTATACGG/ TTTTAGTTATACGTTTGGTTATACGG	*yej*M-*aid*A	*rdg*R

PICI	Strain	Accession number	Size	*att* site core	*att* gene	Accessory genes
SbCISb277	*Shigella boydii Sb227*	CP000036 (2097873-2108605)	10.7	TTTTAGTTATACGCTTAGTTATACGG/ TTTTAGTTATACGTTTGGTTATACGG	*yej*M-*aid*A	*rdg*R; *per*C
DiCI3937	*Dickeya dadantii 3937*	CP002038.1 (3753070-3764812)	11.7	GAGTGGATTTTAGTAGACTTAGGGAAACGT	*pro*A	*yaf*N; *yaf*O
PcCIPCC221	*Pectobacterium carotovorum subsp. carotovorum PCC21*	CP003776.1 (1838416-1848145)	9.7	CTATCGGCATA	CMP	*par*E; *rel*B
PcCISCRI1043	*Pectobacterium carotovora subsp. atroseptica SCRI1043*	NC_004547.2 (3091994-3101397)	9.4	GCATTCTCACTGAGTTTGCGGGTTTTTTGT TGCCTGTTATCTATCGGGATACCCCGTTTA CAGCCGGAAA	*yej*M	*par*E
CfCIP10159	*Citrobacter freundii strain P10159*	CP012554.1 (2684917-2697361)	12.4	ATCGAGTAAG	*yic*C-*yic*G	*per*C
AaCIF0387	*Aggregatibacter aphrophilus F0387*	ACZJ01000009.1 (141541-149751)	8.2	AGTCCTGTTGGG	*rpo*D	
AacCIRhAA1	*Aggregatibacter actinomycetemcomitans RhAA1*	AHGR01000004.1 (357167-365116)	7.9	AAAATAGTACCCATTT	*glt*X-*csp*C	
PhaCIATCC43949	*Photorhabdus asymbiotica ATCC43949*	FM162591 (3134213-3145217)	11	AACAAAATCAAACAA	*tus*E-*cas*I	
NmCIFF6	*Necropsobacter massiliensis genome assembly strain FF6*	CDON01000001 (135049-142542)	7.5	TTTGGTAACACGTTTAGTAACAC	*glt*X-*ton*B	
CnCIND14b	*Cedecea neteri strain ND14b*	CP009460.1 (3954409-3966716)	12.3	TCCTATTATC	ADH-SDR	*yaf*N; *yaf*O

Table S2. EcCICFT073 orthologies.

EcCICFT073 gene	Function	Orthologs	Species	Full name	Length	Similarity	Overlap	Element	Start	End	Size	Comments
c1483	*int*	ecc:c1483	*Escherichia coli*	*Escherichia coli O6:K2:H1 CFT073*	314	1.000		EcCICFT073				
		ece:Z1835	*Escherichia coli*	*Escherichia coli O157:H7 EDL933*	416	0.978	312	PICI?	1678546	1694015	15.5	HNH; *ter*L
		ecs:ECs1574	*Escherichia coli*	*Escherichia coli O157:H7 Sakai*	409	0.978	312	PICI?	1594570	1610042	15.5	Same as *Escherichia coli O157:H7 EDL933*
		elx:CDCO157_1506	*Escherichia coli*	*Escherichia coli O157:H7 Xuzhou21*	409	0.978	312	PICI?	1596512	1611982	15.5	Same as *Escherichia coli O157:H7 EDL933*
		sfe:SFxv_1284	*Shigella flexneri*	*Shigella flexneri 2002017*	416	0.978	312	PICI?	1232114	1246489	14.4	HNH; *ter*L
		sfn:SFy_1623	*Shigella flexneri*	*Shigella flexneri 2003036*	416	0.978	312	PICI?	1198620	1213000	14.4	Same as *Shigella flexneri 2002017*
		sfs:SFyv_1675	*Shigella flexneri*	*Shigella flexneri Shi06HN006*	416	0.978	312	PICI?	1217313	1231688	14.4	Same as *Shigella flexneri 2002017*
		sft:NCTC1_01185	*Shigella flexneri*	*Shigella flexneri NCTC1*	409	0.978	312	PICI?	1133207	1147945	14.7	Similar to *Shigella flexneri 2002017*
		sfx:S1211	*Shigella flexneri*	*Shigella flexneri 2457T*	409	0.978	312	PICI?	1177656	1192394	14.7	Similar to *Shigella flexneri 2002017*
		ecf:ECH74115_1571	*Escherichia coli*	*Escherichia coli O157:H7 EC4115*	409	0.971	312	PICI	1535498	15466717	11.2	
		ecoh:ECRM13516_1440	*Escherichia coli*	*Escherichia coli O145:H28 RM13516*	409	0.971	312	PICI	1438775	1449995	11.2	Same as *Escherichia coli O157:H7 EC4115*
		ecoo:ECRM13514_1484	*Escherichia coli*	*Escherichia coli O145:H28 RM13514*	409	0.971	312	PICI	1462244	1473465	11.2	Same as *Escherichia coli O157:H7 EC4115*
		ect:ECIAI39_2027	*Escherichia coli*	*Escherichia coli O7:K1 IAI39*	416	0.974	312	PICI?	2060161	2074688	14.5	HNH; *ter*L
		elr:ECO55CA74_06770	*Escherichia coli*	*Escherichia coli O55:H7 RM12579*	409	0.971	312	PICI	1438165	1449376	11.2	Same as *Escherichia coli O157:H7 EC4115*
		eok:G2583_1387	*Escherichia coli*	*Escherichia coli O55:H7 CB9615*	416	0.971	312	PICI	1444572	1455794	11.2	Same as *Escherichia coli O157:H7 EC4115*
		etw:ECSP_1490	*Escherichia coli*	*Escherichia coli O157:H7 TW14359*	409	0.971	312	PICI	1535786	1547005	11.2	Same as *Escherichia coli O157:H7 EC4115*
		sfv:SFV_1147	*Shigella flexneri*	*Shigella flexneri 8401*	416	0.974	312	PICI?	1192843	1208358	15.5	Similar to *Shigella flexneri 2002017*
		eoc:CE10_1211	*Escherichia coli*	*Escherichia coli O7:K1 CE10*	409	0.968	312	PICI?	1260279	1274777	14.5	HNH; *ter*L
		eoh:ECO103_1236	*Escherichia coli*	*Escherichia coli O103:H2 12009*	415	0.968	312	PICI	1325875	1336965	11.1	Similar to *Escherichia coli O157:H7 EC4115*
		eoj:ECO26_1646	*Escherichia coli*	*Escherichia coli O26:H11 11368*	409	0.968	312	PICI	1619447	1630554	11.1	Same as *Escherichia coli O157:H7 EC4115*
		elo:EC042_1767	*Escherichia coli*	*Escherichia coli O44:H18 042*	405	0.946	312	PICI	1832521	1843545	11	
		ecq:ECED1_1798	*Escherichia coli*	*Escherichia coli O81 ED1a*	409	0.942	312	PICI?	1739927	1756201	16.3	HNH; *ter*L
		elc:i14_0243	*Escherichia coli*	*Escherichia coli clone D i14*	291	0.862	290	PICI	249047	259688	10.6	
		eld:i02_0243	*Escherichia coli*	*Escherichia coli clone D i2*	291	0.862	290	PICI	249047	259688	10.6	Same as *Escherichia coli clone D i14*
		ecoi:ECOPMV1_00884	*Escherichia coli*	*Escherichia coli PMV-1*	412	0.756	308	PICI	919885	930438	10.5	
		eih:ECOK1_0883	*Escherichia coli*	*Escherichia coli IHE3034*	412	0.756	308	PICI	920272	930825	10.5	Same as *Escherichia coli PMV-1*
		elu:UM146_13240	*Escherichia coli*	*Escherichia coli UM146*	412	0.756	308	PICI	2721434	2731984	10.5	Same as *Escherichia coli PMV-1*
		ssn:SSON_0868	*Shigella sonnei*	*Shigella sonnei Ss046*	412	0.760	308	PICI	922309	936578	14.3	
		ecm:EcSMS35_0939	*Escherichia coli*	*Escherichia coli SMS-3-5*	406	0.685	305	PICI?	955625	970780	15.2	HNH; *ter*L
		yak:ACZ76_07050	*Yersinia aleksiciae*	*Yersinia aleksiciae 159*	407	0.543	3013	PICI?	1512198	1528170	15.9	
		xbv:XBW1_1862	*Xenorhabdus bovienii*	*Xenorhabdus bovienii CS03*	406	0.537	309	Defective PICI?				*att*? Packaging?

EcCICFT073 gene	Function	Orthologs	Species	Full name	Length	Similarity	Overlap	Element	Start	End	Size	Comments
c1487	*icd*-like	ecc:c1487	*Escherichia coli*	*Escherichia coli O6:K2:H1 CFT073*	428	1.000		EcCICFT073				
		elc:i14_0245	*Escherichia coli*	*Escherichia coli clone D i14*	396	0.684	380	PICI	249047	259688	10.6	
		eld:i02_0245	*Escherichia coli*	*Escherichia coli clone D i2*	396	0.684	380	PICI	249047	259688	10.6	Same as *Escherichia coli clone D i14*
		elo:EC042_2166	*Escherichia coli*	*Escherichia coli O44:H18 042*	357	0.630	354	Prophage?				Chimera prophage & PICI
		eoh:ECO103_1238	*Escherichia coli*	*Escherichia coli O103:H2 12009*	342	0.639	319	PICI	1325875	1336965	11.1	Similar to *Escherichia coli O157:H7 EC4115*
		eoj:ECO26_1648	*Escherichia coli*	*Escherichia coli O26:H11 11368*	342	0.639	319	PICI	1619447	1630554	11.1	Same as *Escherichia coli O157:H7 EC4115*
		ect:ECIAI39_2024	*Escherichia coli*	*Escherichia coli O7:K1 IAI39*	353	0.481	337	PICI?	2060161	2074688	14.5	HNH; *ter*L
		ecoo:ECRM13514_4638	*Escherichia coli*	*Escherichia coli O145:H28 RM13514*	488	0.425	334	PICI	4527455	4539396	11.9	
		sbc:SbBS512_E2184	*Shigella boydii*	*Shigella boydii CDC 3083-94*	329	0.429	329	Defective PICI?				Only an *int* fragment and *alp*A
		eab:ECABU_c40880	*Escherichia coli*	*Escherichia coli ABU 83972*	283	0.610	200	Defective PICI?				Only an *int* fragment and *alp*A
		ecf:ECH74115_1572	*Escherichia coli*	*Escherichia coli O157:H7 EC4115*	314	0.422	327	PICI	1535498	15466717	11.2	
c1488		ecc:c1488	*Escherichia coli*	*Escherichia coli O6:K2:H1 CFT073*	76	1.000		EcCICFT073				
		eoi:ECO111_2916	*Escherichia coli*	*Escherichia coli O111:H- 11128*	152	0.903	72	PICI	2855234	2864550	9.3	
		ecq:ECED1_1789	*Escherichia coli*	*Escherichia coli O81 ED1a*	63	0.525	59	PICI?	1739927	1756201	16.3	HNH; *ter*L
		ecs:ECs1579	*Escherichia coli*	*Escherichia coli O157:H7 Sakai*	63	0.525	59	PICI?	1594570	1610042	15.5	Same as *Escherichia coli O157:H7 EDL933*
		elx:CDCO157_1512	*Escherichia coli*	*Escherichia coli O157:H7 Xuzhou21*	63	0.525	59	PICI?	1596512	1611982	15.5	Same as *Escherichia coli O157:H7 EDL933*
		pge:LG71_03455	*Pluralibacter gergoviae*	*Pluralibacter gergoviae FB2*	65	0.541	61	PICI?	713730	733670	19.9	tail
		eclo:ENC_17430	*Enterobacter cloacae*	*Enterobacter cloacae subsp. cloacae NCTC 9394*	63	0.466	58	No insert				
		pfq:QQ39_05060	*Pragia fontium*	*Pragia fontium 24613*	74	0.508	65	Prophage				
		cif:AL515_00785	*Citrobacter*	*Citrobacter sp. FDAARGOS_156*	66	0.492	61	PICI?	164335	175860	11.5	Not commun structure
		xpo:XPG1_0943	*Xenorhabdus poinarii*	*Xenorhabdus poinarii G6*	84	0.519	54	Prophage?				Chimera prophage & PICI
		xbv:XBW1_2816	*Xenorhabdus bovienii*	*Xenorhabdus bovienii CS03*	82	0.400	70	Prophage?				Chimera prophage & PICI
c1490		ecc:c1490	*Escherichia coli*	*Escherichia coli O6:K2:H1 CFT073*	73	1.000		EcCICFT073				
		eoj:ECO26_3108	*Escherichia coli*	*Escherichia coli O26:H11 11368*	80	1.000	71	PICI	3041660	3050231	8.5	*Similar to Escherichia coli O111:H- 11128*
		sfs:SFyv_1680	*Shigella flexneri*	*Shigella flexneri Shi06HN006*	73	0.973	73	PICI?	1217313	1231688	14.4	Same as *Shigella flexneri 2002017*
		sft:NCTC1_01188	*Shigella flexneri*	*Shigella flexneri NCTC1*	73	0.973	73	PICI?	1133207	1147945	14.7	Similar to *Shigella flexneri 2002017*
		eoi:ECO111_2916	*Escherichia coli*	*Escherichia coli O111:H- 11128*	152	0.972	71	PICI	2855234	2864550	9.3	
		elo:EC042_1762	*Escherichia coli*	*Escherichia coli O44:H18 042*	155	0.513	78	PICI	1832521	1843545	11	
		ecoh:ECRM13516_1443	*Escherichia coli*	*Escherichia coli O145:H28 RM13516*	154	0.506	77	PICI	1438775	1449995	11.2	Same as *Escherichia coli O157:H7 EC4115*
		ecoo:ECRM13514_1487	*Escherichia coli*	*Escherichia coli O145:H28 RM13514*	154	0.506	77	PICI	1462244	1473465	11.2	Same as *Escherichia coli O157:H7 EC4115*
		etw:ECSP_1494	*Escherichia coli*	*Escherichia coli O157:H7 TW14359*	154	0.506	77	PICI	1535786	1547005	11.2	Same as *Escherichia coli O157:H7 EC4115*
		eoh:ECO103_1239	*Escherichia coli*	*Escherichia coli O103:H2 12009*	154	0.481	77	PICI	1325875	1336965	11.1	Similar to *Escherichia coli O157:H7 EC4115*

EcCICFT073 gene	Function	Orthologs	Species	Full name	Length	Similarity	Overlap	Element	Start	End	Size	Comments
		elc:i14_0247	*Escherichia coli*	*Escherichia coli clone D i14*	78	0.405	74	PICI	249047	259688	10.6	
c1491		ecc:c1491	*Escherichia coli*	*Escherichia coli O6:K2:H1 CFT073*	79	1.000		EcCICFT073				
		sbo:SBO_2132	*Shigella boydii*	*Shigella boydii Sb227*	93	0.835	79	PICI	2097873	2108560	10.7	
		ecoh:ECRM13516_1443	*Escherichia coli*	*Escherichia coli O145:H28 RM13516*	154	0.855	76	PICI	1438775	1449995	11.2	Same as *Escherichia coli O157:H7 EC4115*
		ecoo:ECRM13514_1487	*Escherichia coli*	*Escherichia coli O145:H28 RM13514*	154	0.855	76	PICI	1462244	1473465	11.2	Same as *Escherichia coli O157:H7 EC4115*
		elo:EC042_1762	*Escherichia coli*	*Escherichia coli O44:H18 042*	155	0.855	76	PICI	1832521	1843545	11	
		eoj:ECO26_1649	*Escherichia coli*	*Escherichia coli O26:H11 11368*	154	0.855	76	PICI	1619447	1630554	11.1	Same as *Escherichia coli O157:H7 EC4115*
		etw:ECSP_1494	*Escherichia coli*	*Escherichia coli O157:H7 TW14359*	154	0.855	76	PICI	1535786	1547005	11.2	Same as *Escherichia coli O157:H7 EC4115*
		eoh:ECO103_1239	*Escherichia coli*	*Escherichia coli O103:H2 12009*	154	0.842	76	PICI	1325875	1336965	11.1	Similar to *Escherichia coli O157:H7 EC4115*
		eoi:ECO111_2917	*Escherichia coli*	*Escherichia coli O111:H- 11128*	77	0.849	73	PICI	2855234	2864550	9.3	
		eok:G2583_1393	*Escherichia coli*	*Escherichia coli O55:H7 CB9615*	77	0.849	73	PICI	1444572	1455794	11.2	Same as *Escherichia coli O157:H7 EC4115*
		sfe:SFxv_1288	*Shigella flexneri*	*Shigella flexneri 2002017*	66	0.981	54	PICI?	1232114	1246489	14.4	HNH; *ter*L
c1492		ecc:c1492	*Escherichia coli*	*Escherichia coli O6:K2:H1 CFT073*	77	1.000		EcCICFT073				
		sfe:SFxv_1289	*Shigella flexneri*	*Shigella flexneri 2002017*	77	0.896	77	PICI?	1232114	1246489	14.4	HNH; *ter*L
		sfl:SF1133a	*Shigella flexneri*	*Shigella flexneri 301*	77	0.896	77	PICI?	1174763	1189501	14.7	Similar to *Shigella flexneri 2002017*
		sfn:SFy_1629	*Shigella flexneri*	*Shigella flexneri 2003036*	77	0.896	77	PICI?	1198620	1213000	14.4	Same as *Shigella flexneri 2002017*
		sfs:SFyv_1682	*Shigella flexneri*	*Shigella flexneri Shi06HN006*	77	0.896	77	PICI?	1217313	1231688	14.4	Same as *Shigella flexneri 2002017*
		sft:NCTC1_01189	*Shigella flexneri*	*Shigella flexneri NCTC1*	77	0.896	77	PICI?	1133207	1147945	14.7	Similar to *Shigella flexneri 2002017*
		ecf:ECH74115_1575	*Escherichia coli*	*Escherichia coli O157:H7 EC4115*	77	0.870	77	PICI	1535498	15466717	11.2	
		ecoh:ECRM13516_1444	*Escherichia coli*	*Escherichia coli O145:H28 RM13516*	77	0.870	77	PICI	1438775	1449995	11.2	Same as *Escherichia coli O157:H7 EC4115*
		ecoo:ECRM13514_0974	*Escherichia coli*	*Escherichia coli O145:H28 RM13514*	77	0.870	77	PICI?	998523	1009658	11.1	*ter*L?
		elo:EC042_1761	*Escherichia coli*	*Escherichia coli O44:H18 042*	77	0.870	77	PICI	1832521	1843545	11	
		elr:ECO55CA74_06805	*Escherichia coli*	*Escherichia coli O55:H7 RM12579*	77	0.870	77	PICI	1438165	1449376	11.2	Same as *Escherichia coli O157:H7 EC4115*
c1493	DUF4222	ecc:c1493	*Escherichia coli*	*Escherichia coli O6:K2:H1 CFT073*	99	1.000		EcCICFT073				
		ecoo:ECRM13514_0975	*Escherichia coli*	*Escherichia coli O145:H28 RM13514*	99	0.939	99	PICI?	998523	1009658	11.1	*ter*L?
		eoh:ECO103_1241	*Escherichia coli*	*Escherichia coli O103:H2 12009*	99	0.939	99	PICI	1325875	1336965	11.1	Similar to *Escherichia coli O157:H7 EC4115*
		eoj:ECO26_1651	*Escherichia coli*	*Escherichia coli O26:H11 11368*	99	0.939	99	PICI	1619447	1630554	11.1	Same as *Escherichia coli O157:H7 EC4115*
		ecf:ECH74115_1576	*Escherichia coli*	*Escherichia coli O157:H7 EC4115*	99	0.929	99	PICI	1535498	15466717	11.2	
		ecoh:ECRM13516_1445	*Escherichia coli*	*Escherichia coli O145:H28 RM13516*	99	0.929	99	PICI	1438775	1449995	11.2	Same as *Escherichia coli O157:H7 EC4115*
		eok:G2583_1395	*Escherichia coli*	*Escherichia coli O55:H7 CB9615*	99	0.929	99	PICI	1444572	1455794	11.2	Same as *Escherichia coli O157:H7 EC4115*
		etw:ECSP_1496	*Escherichia coli*	*Escherichia coli O157:H7 TW14359*	99	0.929	99	PICI	1535786	1547005	11.2	Same as *Escherichia coli O157:H7 EC4115*

EcCICFT073 gene	Function	Orthologs	Species	Full name	Length	Similarity	Overlap	Element	Start	End	Size	Comments
		elo:EC042_1760	*Escherichia coli*	*Escherichia coli O44:H18 042*	99	0.919	99	PICI	1832521	1843545	11	
		eoi:ECO111_2919	*Escherichia coli*	*Escherichia coli O111:H- 11128*	99	0.909	99	PICI	2855234	2864550	9.3	
		ecs:ECs1581	*Escherichia coli*	*Escherichia coli O157:H7 Sakai*	99	0.828	99	PICI?	1594570	1610042	15.5	Same as *Escherichia coli O157:H7 EDL933*
c1495	*pri*	ecc:c1495	*Escherichia coli*	*Escherichia coli O6:K2:H1 CFT073*	584	1.000		EcCICFT073				
		ecoo:ECRM13514_0976	*Escherichia coli*	*Escherichia coli O145:H28 RM13514*	584	0.986	584	PICI?	998523	1009658	11.1	*ter*L?
		ecoi:ECOPMV1_00890	*Escherichia coli*	*Escherichia coli PMV-1*	582	0.916	584	PICI	919885	930438	10.5	
		eih:ECOK1_0889	*Escherichia coli*	*Escherichia coli IHE3034*	582	0.916	584	PICI	920272	930825	10.5	Same as *Escherichia coli PMV-1*
		eoc:CE10_1217	*Escherichia coli*	*Escherichia coli O7:K1 CE10*	582	0.904	584	PICI?	1260279	1274777	14.5	HNH; *ter*L
		ect:ECIAI39_2020	*Escherichia coli*	*Escherichia coli O7:K1 IAI39*	582	0.904	584	PICI?	2060161	2074688	14.5	HNH; *ter*L
		sft:NCTC1_01191	*Shigella flexneri*	*Shigella flexneri NCTC1*	582	0.904	584	PICI?	1133207	1147945	14.7	Similar to *Shigella flexneri 2002017*
		sfl:SF1135	*Shigella flexneri*	*Shigella flexneri 301*	582	0.902	584	PICI?	1174763	1189501	14.7	Similar to *Shigella flexneri 2002017*
		sfs:SFyv_1688	*Shigella flexneri*	*Shigella flexneri Shi06HN006*	582	0.902	584	PICI?	1217313	1231688	14.4	Same as *Shigella flexneri 2002017*
		sfx:S1215	*Shigella flexneri*	*Shigella flexneri 2457T*	582	0.902	584	PICI?	1177656	1192394	14.7	Similar to *Shigella flexneri 2002017*
		sfv:SFV_1150	*Shigella flexneri*	*Shigella flexneri 8401*	582	0.901	584	PICI?	1192843	1208358	15.5	Similar to *Shigella flexneri 2002017*
c1496		ecc:c1496	*Escherichia coli*	*Escherichia coli O6:K2:H1 CFT073*	85	1.000		EcCICFT073				
		ecoi:ECOPMV1_00891	*Escherichia coli*	*Escherichia coli PMV-1*	85	1.000	85	PICI	919885	930438	10.5	
		eih:ECOK1_0890	*Escherichia coli*	*Escherichia coli IHE3034*	85	1.000	85	PICI	920272	930825	10.5	Same as *Escherichia coli PMV-1*
		elu:UM146_13205	*Escherichia coli*	*Escherichia coli UM146*	85	1.000	85	PICI	2721434	2731984	10.5	Same as *Escherichia coli PMV-1*
		ect:ECIAI39_2019	*Escherichia coli*	*Escherichia coli O7:K1 IAI39*	85	0.988	85	PICI?	2060161	2074688	14.5	HNH; *ter*L
		eoc:CE10_1218	*Escherichia coli*	*Escherichia coli O7:K1 CE10*	85	0.988	85	PICI?	1260279	1274777	14.5	HNH; *ter*L
		ecm:EcSMS35_0947	*Escherichia coli*	*Escherichia coli SMS-3-5*	81	0.875	72	PICI?	955625	970780	15.2	HNH; *ter*L
		ssn:SSON_0875	*Shigella sonnei*	*Shigella sonnei Ss046*	81	0.875	72	PICI	922309	936578	14.3	
		eoj:ECO26_2320	*Escherichia coli*	*Escherichia coli O26:H11 11368*	81	0.861	72	PICI?	2239126	2254011	14.9	HNH; *ter*L
		eln:NRG857_05750	*Escherichia coli*	*Escherichia coli O83:H1 NRG 857C*	81	0.833	72	Prophage?				Chimera prophage & PICI
		sfe:SFxv_1293	*Shigella flexneri*	*Shigella flexneri 2002017*	92	0.728	92	PICI?	1232114	1246489	14.4	HNH; *ter*L
c1497	*ssb*	ecc:c1497	*Escherichia coli*	*Escherichia coli O6:K2:H1 CFT073*	136	1.000		EcCICFT073				
		ecq:ECED1_1784	*Escherichia coli*	*Escherichia coli O81 ED1a*	136	1.000	136	PICI?	1739927	1756201	16.3	HNH; *ter*L
		ece:Z1845	*Escherichia coli*	*Escherichia coli O157:H7 EDL933*	136	0.985	136	PICI?	1678546	1694015	15.5	HNH; *ter*L
		ecs:ECs1587	*Escherichia coli*	*Escherichia coli O157:H7 Sakai*	136	0.985	136	PICI?	1594570	1610042	15.5	Same as *Escherichia coli O157:H7 EDL933*
		elx:CDCO157_1520	*Escherichia coli*	*Escherichia coli O157:H7 Xuzhou21*	136	0.985	136	PICI?	1596512	1611982	15.5	Same as *Escherichia coli O157:H7 EDL933*
		eoj:ECO26_2319	*Escherichia coli*	*Escherichia coli O26:H11 11368*	136	0.985	136	PICI?	2239126	2254011	14.9	HNH; *ter*L

EcCICFT073 gene	Function	Orthologs	Species	Full name	Length	Similarity	Overlap	Element	Start	End	Size	Comments
		ecoh:ECRM13516_1448	*Escherichia coli*	*Escherichia coli O145:H28 RM13516*	136	0.978	136	PICI	1438775	1449995	11.2	Same as *Escherichia coli O157:H7 EC4115*
		ecoo:ECRM13514_1492	*Escherichia coli*	*Escherichia coli O145:H28 RM13514*	136	0.978	136	PICI	1462244	1473465	11.2	Same as *Escherichia coli O157:H7 EC4115*
		elo:EC042_1757	*Escherichia coli*	*Escherichia coli O44:H18 042*	136	0.978	136	PICI	1832521	1843545	11	
		elr:ECO55CA74_06820	*Escherichia coli*	*Escherichia coli O55:H7 RM12579*	136	0.978	136	PICI	1438165	1449376	11.2	Same as *Escherichia coli O157:H7 EC4115*
		eoh:ECO103_1244	*Escherichia coli*	*Escherichia coli O103:H2 12009*	136	0.978	136	PICI	1325875	1336965	11.1	Similar to *Escherichia coli O157:H7 EC4115*
c1498		ecc:c1498	*Escherichia coli*	*Escherichia coli O6:K2:H1 CFT073*	68	1.000		EcCICFT073				
		ecq:ECED1_2650	*Escherichia coli*	*Escherichia coli O81 ED1a*	68	0.971	68	PICI	2568989	2580418	11.4	
		ecoh:ECRM13516_1452	*Escherichia coli*	*Escherichia coli O145:H28 RM13516*	68	0.971	68	PICI	1438775	1449995	11.2	Same as *Escherichia coli O157:H7 EC4115*
		ecoo:ECRM13514_1496	*Escherichia coli*	*Escherichia coli O145:H28 RM13514*	68	0.971	68	PICI	1462244	1473465	11.2	Same as *Escherichia coli O157:H7 EC4115*
		eoj:ECO26_1657	*Escherichia coli*	*Escherichia coli O26:H11 11368*	68	0.971	68	PICI	1619447	1630554	11.1	Same as *Escherichia coli O157:H7 EC4115*
		eoh:ECO103_1247	*Escherichia coli*	*Escherichia coli O103:H2 12009*	68	0.956	68	PICI	1325875	1336965	11.1	Similar to *Escherichia coli O157:H7 EC4115*
		elc:i14_0253	*Escherichia coli*	*Escherichia coli clone D i14*	68	0.974	68	PICI	249047	259688	10.6	
		eld:i02_0253	*Escherichia coli*	*Escherichia coli clone D i2*	68	0.974	68	PICI	249047	259688	10.6	Same as *Escherichia coli clone D i14*
		ecoi:ECOPMV1_00893	*Escherichia coli*	*Escherichia coli PMV-1*	68	0.676	68	PICI	919885	930438	10.5	
		eih:ECOK1_0892	*Escherichia coli*	*Escherichia coli IHE3034*	68	0.676	68	PICI	920272	930825	10.5	Same as *Escherichia coli PMV-1*
		elo:EC042_1754	*Escherichia coli*	*Escherichia coli O44:H18 042*	79	0.676	68	PICI	1832521	1843545	11	
c1499	*capsid*	ecc:c1499	*Escherichia coli*	*Escherichia coli O6:K2:H1 CFT073*	351	1.000		EcCICFT073				
		ecoh:ECRM13516_1453	*Escherichia coli*	*Escherichia coli O145:H28 RM13516*	351	0.966	350	PICI	1438775	1449995	11.2	Same as *Escherichia coli O157:H7 EC4115*
		eoh:ECO103_1248	*Escherichia coli*	*Escherichia coli O103:H2 12009*	351	0.966	350	PICI	1325875	1336965	11.1	Similar to *Escherichia coli O157:H7 EC4115*
		eoj:ECO26_1658	*Escherichia coli*	*Escherichia coli O26:H11 11368*	351	0.966	350	PICI	1619447	1630554	11.1	Same as *Escherichia coli O157:H7 EC4115*
		ecoo:ECRM13514_1497	*Escherichia coli*	*Escherichia coli O145:H28 RM13514*	351	0.963	350	PICI	1462244	1473465	11.2	Same as *Escherichia coli O157:H7 EC4115*
		ecf:ECH74115_1583	*Escherichia coli*	*Escherichia coli O157:H7 EC4115*	351	0.969	350	PICI	1535498	15466717	11.2	
		elo:EC042_1753	*Escherichia coli*	*Escherichia coli O44:H18 042*	351	0.969	350	PICI	1832521	1843545	11	
		elr:ECO55CA74_06840	*Escherichia coli*	*Escherichia coli O55:H7 RM12579*	351	0.969	350	PICI	1438165	1449376	11.2	Same as *Escherichia coli O157:H7 EC4115*
		eok:G2583_1400	*Escherichia coli*	*Escherichia coli O55:H7 CB9615*	351	0.969	350	PICI	1444572	1455794	11.2	Same as *Escherichia coli O157:H7 EC4115*
		etw:ECSP_1503	*Escherichia coli*	*Escherichia coli O157:H7 TW14359*	351	0.969	350	PICI	1535786	1547005	11.2	Same as *Escherichia coli O157:H7 EC4115*
		sbo:SBO_2125	*Shigella boydii*	*Shigella boydii Sb227*	351	0.960	350	PICI	2097873	2108560	10.7	
c1500	HDHD	ecc:c1500	*Escherichia coli*	*Escherichia coli O6:K2:H1 CFT073*	111	1.000		EcCICFT073				
		ecoi:ECOPMV1_00895	*Escherichia coli*	*Escherichia coli PMV-1*	111	0.991	111	PICI	919885	930438	10.5	
		eih:ECOK1_0894	*Escherichia coli*	*Escherichia coli IHE3034*	111	0.991	111	PICI	920272	930825	10.5	Same as *Escherichia coli PMV-1*
		elu:UM146_13185	*Escherichia coli*	*Escherichia coli UM146*	111	0.991	111	PICI	2721434	2731984	10.5	Same as *Escherichia coli PMV-1*

EcCICFT073 gene	Function	Orthologs	Species	Full name	Length	Similarity	Overlap	Element	Start	End	Size	Comments
		ecf:ECH74115_1584	*Escherichia coli*	*Escherichia coli O157:H7 EC4115*	111	0.982	111	PICI	1535498	15466717	11.2	
		elo:EC042_1752	*Escherichia coli*	*Escherichia coli O44:H18 042*	111	0.982	111	PICI	1832521	1843545	11	
		elr:ECO55CA74_06845	*Escherichia coli*	*Escherichia coli O55:H7 RM12579*	111	0.982	111	PICI	1438165	1449376	11.2	Same as *Escherichia coli O157:H7 EC4115*
		eok:G2583_1401	*Escherichia coli*	*Escherichia coli O55:H7 CB9615*	111	0.982	111	PICI	1444572	1455794	11.2	Same as *Escherichia coli O157:H7 EC4115*
		etw:ECSP_1504	*Escherichia coli*	*Escherichia coli O157:H7 TW14359*	111	0.982	111	PICI	1535786	1547005	11.2	Same as *Escherichia coli O157:H7 EC4115*
		ecq:ECED1_2652	*Escherichia coli*	*Escherichia coli O81 ED1a*	111	0.973	111	PICI	2568989	2580418	11.4	
		elc:i14_0255	*Escherichia coli*	*Escherichia coli clone D i14*	111	0.973	111	PICI	249047	259688	10.6	
c1501		ecc:c1501	*Escherichia coli*	*Escherichia coli O6:K2:H1 CFT073*	137	1.000		EcCICFT073				
		ecoi:ECOPMV1_00896	*Escherichia coli*	*Escherichia coli PMV-1*	137	0.993	137	PICI	919885	930438	10.5	
		eih:ECOK1_0895	*Escherichia coli*	*Escherichia coli IHE3034*	137	0.993	137	PICI	920272	930825	10.5	Same as *Escherichia coli PMV-1*
		elu:UM146_13180	*Escherichia coli*	*Escherichia coli UM146*	137	0.993	137	PICI	2721434	2731984	10.5	Same as *Escherichia coli PMV-1*
		eok:G2583_1402	*Escherichia coli*	*Escherichia coli O55:H7 CB9615*	137	0.971	137	PICI	1444572	1455794	11.2	Same as *Escherichia coli O157:H7 EC4115*
		elr:ECO55CA74_06850	*Escherichia coli*	*Escherichia coli O55:H7 RM12579*	137	0.964	137	PICI	1438165	1449376	11.2	Same as *Escherichia coli O157:H7 EC4115*
		ecf:ECH74115_1585	*Escherichia coli*	*Escherichia coli O157:H7 EC4115*	137	0.964	137	PICI	1535498	15466717	11.2	
		etw:ECSP_1505	*Escherichia coli*	*Escherichia coli O157:H7 TW14359*	137	0.964	137	PICI	1535786	1547005	11.2	Same as *Escherichia coli O157:H7 EC4115*
		ecoh:ECRM13516_1455	*Escherichia coli*	*Escherichia coli O145:H28 RM13516*	137	0.942	137	PICI	1438775	1449995	11.2	Same as *Escherichia coli O157:H7 EC4115*
		ecoo:ECRM13514_1499	*Escherichia coli*	*Escherichia coli O145:H28 RM13514*	137	0.942	137	PICI	1462244	1473465	11.2	Same as *Escherichia coli O157:H7 EC4115*
		eoh:ECO103_1250	*Escherichia coli*	*Escherichia coli O103:H2 12009*	137	0.942	137	PICI	1325875	1336965	11.1	Similar to *Escherichia coli O157:H7 EC4115*
c1502		ecc:c1502	*Escherichia coli*	*Escherichia coli O6:K2:H1 CFT073*	60	1.000		EcCICFT073				
		sbo:SBO_2122	*Shigella boydii*	*Shigella boydii Sb227*	147	0.933	60	PICI	2097873	2108560	10.7	
c1503		ecc:c1503	*Escherichia coli*	*Escherichia coli O6:K2:H1 CFT073*	113	1.000		EcCICFT073				
		sbo:SBO_2121	*Shigella boydii*	*Shigella boydii Sb227*	113	1.000	113	PICI	2097873	2108560	10.7	
		elo:EC042_1750	*Escherichia coli*	*Escherichia coli O44:H18 042*	113	0.982	113	PICI	1832521	1843545	11	
		aaw:AVL56_03045	*Alteromonas addita*	*Alteromonas addita R10SW13*	774	0.349	106	No insert				
		asp:AOR13_2906	*Alteromonas addita*	*Alteromonas stellipolaris LMG 21856*	774	0.349	106	No insert				
		asq:AVL57_04270	*Alteromonas addita*	*Alteromonas stellipolaris LMG 21861*	774	0.349	106	No insert				
		pte:PTT_18114	*Pyrenophora teres*	*Pyrenophora teres f. teres 0-1*	380	0.318	88					RefSeq (Assembly: GCA_000166005.1)
		bve:AK36_4596	*Burkholderia vietnamiensis*	*Burkholderia vietnamiensis LMG 10929*	402	0.321	81	No insert				
		miu:ABE85_20515	*Mitsuaria*	*Mitsuaria sp. 7*	285	0.322	87	No insert				
		nda:Ndas_4923	*Nocardiopsis dassonvillei*	*Nocardiopsis dassonvillei subsp. dassonvillei DSM 43111*	768	0.308	117	No insert				
		spaa:SPAPADRAFT_153792	*Spathaspora passalidarum*	*Spathaspora passalidarum NRRL Y-27907*	711	0.309	81					RefSeq (Assembly: GCF_000223485.1)

EcCICFT073 gene	Function	Orthologs	Species	Full name	Length	Similarity	Overlap	Element	Start	End	Size	Comments
c1504		ecc:c1504	*Escherichia coli*	*Escherichia coli O6:K2:H1 CFT073*	53	1.000		EcCICFT073				
		ola:105353925	*Oryzias latipes*	*Oryzias latipes*	372	0.372	42					RefSeq (Assembly: GCF_000313675.1)
c1505		ecc:c1505	*Escherichia coli*	*Escherichia coli O6:K2:H1 CFT073*	93	1.000		EcCICFT073				
		sfe:SFxv_1307	*Shigella flexneri*	*Shigella flexneri 2002017*	93	0.914	93	1232114	1246489	14.4	HNH; *ter*L	
		sfl:SF1147	*Shigella flexneri*	*Shigella flexneri 301*	93	0.914	93	PICI?	1174763	1189501	14.7	Similar to *Shigella flexneri 2002017*
		sfn:SFy_1654	*Shigella flexneri*	*Shigella flexneri 2003036*	93	0.914	93	PICI?	1198620	1213000	14.4	Same as *Shigella flexneri 2002017*
		sfs:SFyv_1706	*Shigella flexneri*	*Shigella flexneri Shi06HN006*	93	0.914	93	PICI?	1217313	1231688	14.4	Same as *Shigella flexneri 2002017*
		sft:NCTC1_01205	*Shigella flexneri*	*Shigella flexneri NCTC1*	93	0.914	93	PICI?	1133207	1147945	14.7	Similar to *Shigella flexneri 2002017*
		sfv:SFV_1162	*Shigella flexneri*	*Shigella flexneri 8401*	93	0.914	93	PICI?	1192843	1208358	15.5	Similar to *Shigella flexneri 2002017*
		sfx:S1229	*Shigella flexneri*	*Shigella flexneri 2457T*	93	0.914	93	PICI?	1177656	1192394	14.7	Similar to *Shigella flexneri 2002017*
		eih:ECOK1_0897	*Escherichia coli*	*Escherichia coli IHE3034*	93	0.914	93	PICI	920272	930825	10.5	Same as *Escherichia coli PMV-1*
		elo:EC042_1749	*Escherichia coli*	*Escherichia coli O44:H18 042*	93	0.914	93	PICI	1832521	1843545	11	
		sbo:SBO_2120	*Shigella boydii*	*Shigella boydii Sb227*	93	0.914	93	PICI	2097873	2108560	10.7	
c1506		ecc:c1506	*Escherichia coli*	*Escherichia coli O6:K2:H1 CFT073*	145	1.000		EcCICFT073				
		ecoi:ECOPMV1_00898	*Escherichia coli*	*Escherichia coli PMV-1*	145	0.936	140	PICI	919885	930438	10.5	
		elu:UM146_13170	*Escherichia coli*	*Escherichia coli UM146*	168	0.936	140	PICI	2721434	2731984	10.5	Same as *Escherichia coli PMV-1*
		sbo:SBO_2119	*Shigella boydii*	*Shigella boydii Sb227*	145	0.929	140	PICI	2097873	2108560	10.7	
		ece:Z1856	*Escherichia coli*	*Escherichia coli O157:H7 EDL933*	112	0.836	110	PICI?	1678546	1694015	15.5	HNH; *ter*L
		tbr:Tb10.70.3460	*Trypanosoma brucei*	*Trypanosoma brucei brucei TREU927*	2151	0.329	70					RefSeq (Assembly: GCF_000002445.1)
		mamo:A6B35_05040	*Mesorhizobium amorphae*	*Mesorhizobium amorphae CCNWGS0123*	278	0.314	102	No insert				
		apac:S7S_10755	*Alcanivorax pacificus*	*Alcanivorax pacificus W11-5*	223	0.318	66	No insert				
		phi:102103469	*Pseudopodoces humilis*	*Pseudopodoces humilis*	172	0.380	79					RefSeq (Assembly: GCF_000331425.1)
		sbq:101031964	*Saimiri boliviensis*	*Saimiri boliviensis boliviensis*	1243	0.333	51					RefSeq (Assembly: GCF_000235385.1)
		cin:100186217	*Ciona intestinalis*	*Ciona intestinalis*	2053	0.304	69					RefSeq (Assembly: GCF_000224145.2)
c1507		ecc:c1507	*Escherichia coli*	*Escherichia coli O6:K2:H1 CFT073*	46	1.000		EcCICFT073				No matches in database (DB)

Table S3. EcCIO42 orthologies.

EcCIO42 gene	Function	Orthologs	Species	Full name	Length	Similarity	Overlap	Element	Start	End	Size	Comments
EC042_1767	*int*	elo:EC042_1767	*Escherichia coli*	*Escherichia coli O44:H18 042*	405	1.000		EcCIO42				
		eoj:ECO26_2328	*Escherichia coli*	*Escherichia coli O26:H11 11368*	405	0.985	405	PICI?	2239126	2254011	14.9	HNH; *ter*L
		ecq:ECED1_1798	*Escherichia coli*	*Escherichia coli O81 ED1a*	409	0.983	405	PICI?	1739927	1756201	16.3	HNH; *ter*L
		ecf:ECH74115_1571	*Escherichia coli*	*Escherichia coli O157:H7 EC4115*	409	0.921	405	PICI	1535498	15466717	11.2	
		ecoh:ECRM13516_1440	*Escherichia coli*	*Escherichia coli O145:H28 RM13516*	409	0.921	405	PICI	1438775	1449995	11.2	Same as *Escherichia coli O157:H7 EC4115*
		ecoo:ECRM13514_1484	*Escherichia coli*	*Escherichia coli O145:H28 RM13514*	409	0.921	405	PICI	1462244	1473465	11.2	Same as *Escherichia coli O157:H7 EC4115*
		elr:ECO55CA74_06770	*Escherichia coli*	*Escherichia coli O55:H7 RM12579*	409	0.921	405	PICI	1438165	1449376	11.2	Same as *Escherichia coli O157:H7 EC4115*
		eok:G2583_1387	*Escherichia coli*	*Escherichia coli O55:H7 CB9615*	416	0.921	405	PICI	1444572	1455794	11.2	Same as *Escherichia coli O157:H7 EC4115*
		etw:ECSP_1490	*Escherichia coli*	*Escherichia coli O157:H7 TW14359*	409	0.921	405	PICI	1535786	1547005	11.2	Same as *Escherichia coli O157:H7 EC4115*
		ece:Z1835	*Escherichia coli*	*Escherichia coli O157:H7 EDL933*	416	0.921	405	PICI?	1678546	1694015	15.5	HNH; *ter*L
		ecs:ECs1574	*Escherichia coli*	*Escherichia coli O157:H7 Sakai*	409	0.921	405	PICI?	1594570	1610042	15.5	Same as *Escherichia coli O157:H7 EDL933*
		elx:CDCO157_1506	*Escherichia coli*	*Escherichia coli O157:H7 Xuzhou21*	409	0.921	405	PICI?	1596512	1611982	15.5	Same as *Escherichia coli O157:H7 EDL933*
		sfe:SFxv_1284	*Shigella flexneri*	*Shigella flexneri 2002017*	416	0.921	405	PICI?	1232114	1246489	14.4	HNH; *ter*L
		sfn:SFy_1623	*Shigella flexneri*	*Shigella flexneri 2003036*	416	0.921	405	PICI?	1198620	1213000	14.4	Same as *Shigella flexneri 2002017*
		sfs:SFyv_1675	*Shigella flexneri*	*Shigella flexneri Shi06HN006*	416	0.921	405	PICI?	1217313	1231688	14.4	Same as *Shigella flexneri 2002017*
		sft:NCTC1_01185	*Shigella flexneri*	*Shigella flexneri NCTC1*	409	0.921	405	PICI?	1133207	1147945	14.7	Similar to *Shigella flexneri 2002017*
		sfx:S1211	*Shigella flexneri*	*Shigella flexneri 2457T*	409	0.921	405	PICI?	1177656	1192394	14.7	Similar to *Shigella flexneri 2002017*
		ect:ECIAI39_2027	*Escherichia coli*	*Escherichia coli O7:K1 IAI39*	416	0.919	405	PICI?	2060161	2074688	14.5	HNH; *ter*L
		sfv:SFV_1147	*Shigella flexneri*	*Shigella flexneri 8401*	416	0.919	405	PICI?	1192843	1208358	15.5	Similar to *Shigella flexneri 2002017*
		eoc:CE10_1211	*Escherichia coli*	*Escherichia coli O7:K1 CE10*	409	0.911	405	PICI?	1260279	1274777	14.5	HNH; *ter*L
		eoh:ECO103_1236	*Escherichia coli*	*Escherichia coli O103:H2 12009*	415	0.909	405	PICI	1325875	1336965	11.1	Similar to *Escherichia coli O157:H7 EC4115*
		ecoi:ECOPMV1_00884	*Escherichia coli*	*Escherichia coli PMV-1*	412	0.754	399	PICI	919885	930438	10.5	
		eih:ECOK1_0883	*Escherichia coli*	*Escherichia coli IHE3034*	412	0.754	399	PICI	920272	930825	10.5	Same as *Escherichia coli PMV-1*
		elu:UM146_13240	*Escherichia coli*	*Escherichia coli UM146*	412	0.754	399	PICI	2721434	2731984	10.5	Same as *Escherichia coli PMV-1*
EC042_1766	*alpA*	elo:EC042_1766	*Escherichia coli*	*Escherichia coli O44:H18 042*	62	1.000		EcCIO42				
		ect:ECIAI39_2026	*Escherichia coli*	*Escherichia coli O7:K1 IAI39*	62	1.000	62	PICI?	2060161	2074688	14.5	HNH; *ter*L
		elr:ECO55CA74_06775	*Escherichia coli*	*Escherichia coli O55:H7 RM12579*	62	1.000	62	PICI	1438165	1449376	11.2	Same as *Escherichia coli O157:H7 EC4115*
		elx:CDCO157_1507	*Escherichia coli*	*Escherichia coli O157:H7 Xuzhou21*	62	1.000	62	PICI?	1596512	1611982	15.5	Same as *Escherichia coli O157:H7 EDL933*
		eok:G2583_1388	*Escherichia coli*	*Escherichia coli O55:H7 CB9615*	62	1.000	62	PICI	1444572	1455794	11.2	Same as *Escherichia coli O157:H7 EC4115*
		etw:ECSP_1491	*Escherichia coli*	*Escherichia coli O157:H7 TW14359*	62	1.000	62	PICI	1535786	1547005	11.2	Same as *Escherichia coli O157:H7 EC4115*
		ecq:ECED1_1797	*Escherichia coli*	*Escherichia coli O81 ED1a*	62	0.984	62	PICI?	1739927	1756201	16.3	HNH; *ter*L
		eoh:ECO103_1237	*Escherichia coli*	*Escherichia coli O103:H2 12009*	106	0.984	62	PICI	1325875	1336965	11.1	Similar to *Escherichia coli O157:H7 EC4115*

EcCIO42 gene	Function	Orthologs	Species	Full name	Length	Similarity	Overlap	Element	Start	End	Size	Comments
		eoj:ECO26_1647	*Escherichia coli*	*Escherichia coli O26:H11 11368*	89	0.984	62	PICI	1619447	1630554	11.1	Same as *Escherichia coli O157:H7 EC4115*
		ecm:EcSMS35_0941	*Escherichia coli*	*Escherichia coli SMS-3-5*	74	0.741	54	PICI?	955625	970780	15.2	HNH; *ter*L
		ddd:Dda3937_01590	*Dickeya dadantii*	*Dickeya dadantii 3937*	86	0.698	53	PICI?	296519	307403	10.8	tail protein
		ecoi:ECOPMV1_00885	*Escherichia coli*	*Escherichia coli PMV-1*	69	0.678	56	PICI	919885	930438	10.5	
		ecoo:ECRM13514_0968	*Escherichia coli*	*Escherichia coli O145:H28 RM13514*	74	0.704	54	PICI?	998523	1009658	11.1	*ter*L?
		eih:ECOK1_0884	*Escherichia coli*	*Escherichia coli IHE3034*	69	0.679	56	PICI	920272	930825	10.5	Same as *Escherichia coli PMV-1*
		elu:UM146_13235	*Escherichia coli*	*Escherichia coli UM146*	69	0.679	56	PICI	2721434	2731984	10.5	Same as *Escherichia coli PMV-1*
		eas:Entas_3977	*Enterobacter asburiae*	*Enterobacter asburiae LF7a*	69	0.660	53	PICI?	4253162	4268649	15.5	*ter*L
		raa:Q7S_08695	*Rahnella aquatilis*	*Rahnella aquatilis HX2*	100	0.654	52	Phage				
		sbc:SbBS512_E2186	*Shigella boydii*	*Shigella boydii CDC 3083-94*	85	0.556	54	Defective PICI?				Only an *int* fragment, *alp*A…
EC042_1765		elo:EC042_1765	*Escherichia coli*	*Escherichia coli O44:H18 042*	263	1.000		EcCIO42				
		eoh:ECO103_1238	*Escherichia coli*	*Escherichia coli O103:H2 12009*	342	0.738	260	PICI	1325875	1336965	11.1	Similar to *Escherichia coli O157:H7 EC4115*
		eoj:ECO26_1648	*Escherichia coli*	*Escherichia coli O26:H11 11368*	342	0.738	260	PICI	1619447	1630554	11.1	Same as *Escherichia coli O157:H7 EC4115*
		ecs:ECs1576	*Escherichia coli*	*Escherichia coli O157:H7 Sakai*	247	0.662	269	PICI?	1594570	1610042	15.5	Same as *Escherichia coli O157:H7 EDL933*
		elx:CDCO157_1508	*Escherichia coli*	*Escherichia coli O157:H7 Xuzhou21*	247	0.662	269	PICI?	1596512	1611982	15.5	Same as *Escherichia coli O157:H7 EDL933*
		ecf:ECH74115_1572	*Escherichia coli*	*Escherichia coli O157:H7 EC4115*	314	0.686	236	PICI	1535498	15466717	11.2	
		ecoh:ECRM13516_1441	*Escherichia coli*	*Escherichia coli O145:H28 RM13516*	314	0.686	236	PICI	1438775	1449995	11.2	Same as *Escherichia coli O157:H7 EC4115*
		ecoo:ECRM13514_1485	*Escherichia coli*	*Escherichia coli O145:H28 RM13514*	314	0.686	236	PICI	1462244	1473465	11.2	Same as *Escherichia coli O157:H7 EC4115*
		eok:G2583_1390	*Escherichia coli*	*Escherichia coli O55:H7 CB9615*	314	0.700	233	PICI	1444572	1455794	11.2	Same as *Escherichia coli O157:H7 EC4115*
		etw:ECSP_1492	*Escherichia coli*	*Escherichia coli O157:H7 TW14359*	314	0.700	233	PICI	1535786	1547005	11.2	Same as *Escherichia coli O157:H7 EC4115*
		ect:ECIAI39_2024	*Escherichia coli*	*Escherichia coli O7:K1 IAI39*	353	0.546	273	PICI?	2060161	2074688	14.5	HNH; *ter*L
EC042_1764		elo:EC042_1764	*Escherichia coli*	*Escherichia coli O44:H18 042*	65	1.000		EcCIO42				
		ecf:ECH74115_1572	*Escherichia coli*	*Escherichia coli O157:H7 EC4115*	314	1.000	65	PICI	1535498	15466717	11.2	
		ecoh:ECRM13516_1441	*Escherichia coli*	*Escherichia coli O145:H28 RM13516*	314	1.000	65	PICI	1438775	1449995	11.2	Same as *Escherichia coli O157:H7 EC4115*
		ecoo:ECRM13514_1485	*Escherichia coli*	*Escherichia coli O145:H28 RM13514*	314	1.000	65	PICI	1462244	1473465	11.2	Same as *Escherichia coli O157:H7 EC4115*
		elr:ECO55CA74_06785	*Escherichia coli*	*Escherichia coli O55:H7 RM12579*	109	1.000	65	PICI	1438165	1449376	11.2	Same as *Escherichia coli O157:H7 EC4115*
		eok:G2583_1390	*Escherichia coli*	*Escherichia coli O55:H7 CB9615*	314	1.000	65	PICI	1444572	1455794	11.2	Same as *Escherichia coli O157:H7 EC4115*
		etw:ECSP_1492	*Escherichia coli*	*Escherichia coli O157:H7 TW14359*	314	1.000	65	PICI	1535786	1547005	11.2	Same as *Escherichia coli O157:H7 EC4115*
		ecs:ECs1577	*Escherichia coli*	*Escherichia coli O157:H7 Sakai*	65	0.953	64	PICI?	1594570	1610042	15.5	Same as *Escherichia coli O157:H7 EDL933*
		elx:CDCO157_1510	*Escherichia coli*	*Escherichia coli O157:H7 Xuzhou21*	65	0.953	64	PICI?	1596512	1611982	15.5	Same as *Escherichia coli O157:H7 EDL933*
		sbc:SbBS512_E2184	*Shigella boydii*	*Shigella boydii CDC 3083-94*	329	0.952	63	Defective PICI?				Only an *int* fragment, *alp*A…
		sfe:SFxv_1287	*Shigella flexneri*	*Shigella flexneri 2002017*	100	0.986	60	PICI?	1232114	1246489	14.4	HNH; *ter*L

EcCIO42 gene	Function	Orthologs	Species	Full name	Length	Similarity	Overlap	Element	Start	End	Size	Comments
		sft:NCTC1_01187	*Shigella flexneri*	*Shigella flexneri NCTC1*	66	0.986	60	PICI?	1133207	1147945	14.7	Similar to *Shigella flexneri 2002017*
		sfx:S1212	*Shigella flexneri*	*Shigella flexneri 2457T*	100	0.986	60	PICI?	1177656	1192394	14.7	Similar to *Shigella flexneri 2002017*
		eoj:ECO26_3107	*Escherichia coli*	*Escherichia coli O26:H11 11368*	186	0.810	58	PICI	3041660	3050231	8.5	*Similar to Escherichia coli O111:H-11128*
EC042_1763		elo:EC042_1763	*Escherichia coli*	*Escherichia coli O44:H18 042*	70	1.000		EcCIO42				
		ecf:ECH74115_1573	*Escherichia coli*	*Escherichia coli O157:H7 EC4115*	70	1.000	70	PICI	1535498	15466717	11.2	
		ecoh:ECRM13516_1442	*Escherichia coli*	*Escherichia coli O145:H28 RM13516*	70	1.000	70	PICI	1438775	1449995	11.2	Same as *Escherichia coli O157:H7 EC4115*
		ecoo:ECRM13514_1486	*Escherichia coli*	*Escherichia coli O145:H28 RM13514*	70	1.000	70	PICI	1462244	1473465	11.2	Same as *Escherichia coli O157:H7 EC4115*
		elr:ECO55CA74_06790	*Escherichia coli*	*Escherichia coli O55:H7 RM12579*	70	1.000	70	PICI	1438165	1449376	11.2	Same as *Escherichia coli O157:H7 EC4115*
		eok:G2583_1391	*Escherichia coli*	*Escherichia coli O55:H7 CB9615*	70	1.000	70	PICI	1444572	1455794	11.2	Same as *Escherichia coli O157:H7 EC4115*
		etw:ECSP_1493	*Escherichia coli*	*Escherichia coli O157:H7 TW14359*	70	1.000	70	PICI	1535786	1547005	11.2	Same as *Escherichia coli O157:H7 EC4115*
		eck:EC55989_0274	*Escherichia coli*	*Escherichia coli 55989*	121	0.661	59	PICI	296286	311648	15.3	
		esl:O3K_20150	*Escherichia coli*	*Escherichia coli O104:H4 2011C-3493*	121	0.661	59	Prophage?				Chimera prophage & PICI
		eab:ECABU_c40900	*Escherichia coli*	*Escherichia coli ABU 83972*	118	0.627	59	PICI?	4152946	4166644	13.7	*ter*L?
		pcv:BCS7_08090	*Pectobacterium carotovorum*	*Pectobacterium carotovorum subsp. odoriferum*	69	0.351	57	PICI	1837956	1854875	16.9	
EC042_1762		elo:EC042_1762	*Escherichia coli*	*Escherichia coli O44:H18 042*	155	1.000		EcCIO42				
		ecoh:ECRM13516_1443	*Escherichia coli*	*Escherichia coli O145:H28 RM13516*	154	0.994	155	PICI	1438775	1449995	11.2	Same as *Escherichia coli O157:H7 EC4115*
		ecoo:ECRM13514_1487	*Escherichia coli*	*Escherichia coli O145:H28 RM13514*	154	0.994	155	PICI	1462244	1473465	11.2	Same as *Escherichia coli O157:H7 EC4115*
		etw:ECSP_1494	*Escherichia coli*	*Escherichia coli O157:H7 TW14359*	154	0.994	155	PICI	1535786	1547005	11.2	Same as *Escherichia coli O157:H7 EC4115*
		eoj:ECO26_1649	*Escherichia coli*	*Escherichia coli O26:H11 11368*	154	0.942	155	PICI	1619447	1630554	11.1	Same as *Escherichia coli O157:H7 EC4115*
		eoh:ECO103_1239	*Escherichia coli*	*Escherichia coli O103:H2 12009*	154	0.935	155	PICI	1325875	1336965	11.1	Similar to *Escherichia coli O157:H7 EC4115*
		sbo:SBO_2132	*Escherichia coli*	*Shigella boydii Sb227*	93	0.988	80	PICI	2097873	2108560	10.7	
		eoi:ECO111_2917	*Escherichia coli*	*Escherichia coli O111:H- 11128*	77	1.000	77	PICI	2855234	2864550	9.3	
		eok:G2583_1393	*Escherichia coli*	*Escherichia coli O55:H7 CB9615*	77	1.000	77	PICI	1444572	1455794	11.2	Same as *Escherichia coli O157:H7 EC4115*
		ecc:c1491	*Escherichia coli*	*Escherichia coli O6:K2:H1 CFT073*	79	0.855	76	PICI	1377749	1389210	11.4	
		elc:i14_0247	*Escherichia coli*	*Escherichia coli clone D i14*	78	0.836	73	PICI	249047	259688	10.6	
EC042_1761		elo:EC042_1761	*Escherichia coli*	*Escherichia coli O44:H18 042*	77	1.000		EcCIO42				
		ecf:ECH74115_1575	*Escherichia coli*	*Escherichia coli O157:H7 EC4115*	77	1.000	77	PICI	1535498	15466717	11.2	
		ecoh:ECRM13516_1444	*Escherichia coli*	*Escherichia coli O145:H28 RM13516*	77	1.000	77	PICI	1438775	1449995	11.2	Same as *Escherichia coli O157:H7 EC4115*
		ecoo:ECRM13514_0974	*Escherichia coli*	*Escherichia coli O145:H28 RM13514*	77	1.000	77	PICI?	998523	1009658	11.1	*ter*L?
		elr:ECO55CA74_06805	*Escherichia coli*	*Escherichia coli O55:H7 RM12579*	77	1.000	77	PICI	1438165	1449376	11.2	Same as *Escherichia coli O157:H7 EC4115*
		eok:G2583_1394	*Escherichia coli*	*Escherichia coli O55:H7 CB9615*	77	1.000	77	PICI	1444572	1455794	11.2	Same as *Escherichia coli O157:H7 EC4115*

EcCIO42 gene	Function	Orthologs	Species	Full name	Length	Similarity	Overlap	Element	Start	End	Size	Comments
		etw:ECSP_1495	*Escherichia coli*	*Escherichia coli O157:H7 TW14359*	77	1.000	77	PICI	1535786	1547005	11.2	Same as *Escherichia coli O157:H7 EC4115*
		eoh:ECO103_1240	*Escherichia coli*	*Escherichia coli O103:H2 12009*	77	0.974	77	PICI	1325875	1336965	11.1	Similar to *Escherichia coli O157:H7 EC4115*
		eoj:ECO26_1650	*Escherichia coli*	*Escherichia coli O26:H11 11368*	77	0.974	77	PICI	1619447	1630554	11.1	Same as *Escherichia coli O157:H7 EC4115*
		sfe:SFxv_1289	*Shigella flexneri*	*Shigella flexneri 2002017*	77	0.974	77	PICI?	1232114	1246489	14.4	HNH; *ter*L
		sfl:SF1133a	*Shigella flexneri*	*Shigella flexneri 301*	77	0.974	77	PICI?	1174763	1189501	14.7	Similar to *Shigella flexneri 2002017*
		sfn:SFy_1629	*Shigella flexneri*	*Shigella flexneri 2003036*	77	0.974	77	PICI?	1198620	1213000	14.4	Same as *Shigella flexneri 2002017*
		sfs:SFyv_1682	*Shigella flexneri*	*Shigella flexneri Shi06HN006*	77	0.974	77	PICI?	1217313	1231688	14.4	Same as *Shigella flexneri 2002017*
		sft:NCTC1_01189	*Shigella flexneri*	*Shigella flexneri NCTC1*	77	0.974	77	PICI?	1133207	1147945	14.7	Similar to *Shigella flexneri 2002017*
		ecc:c1492	*Escherichia coli*	*Escherichia coli O6:K2:H1 CFT073*	77	0.870	77	PICI	1377749	1389210	11.4	
		eoi:ECO111_2918	*Escherichia coli*	*Escherichia coli O111:H- 11128*	77	0.766	77	PICI	2855234	2864550	9.3	
EC042_1760		elo:EC042_1760	*Escherichia coli*	*Escherichia coli O44:H18 042*	99	1.000		EcCIO42				
		ecf:ECH74115_1576	*Escherichia coli*	*Escherichia coli O157:H7 EC4115*	99	0.990	99	PICI	1535498	15466717	11.2	
		ecoh:ECRM13516_1445	*Escherichia coli*	*Escherichia coli O145:H28 RM13516*	99	0.990	99	PICI	1438775	1449995	11.2	Same as *Escherichia coli O157:H7 EC4115*
		ecoo:ECRM13514_1489	*Escherichia coli*	*Escherichia coli O145:H28 RM13514*	99	0.990	99	PICI	1462244	1473465	11.2	Same as *Escherichia coli O157:H7 EC4115*
		eok:G2583_1395	*Escherichia coli*	*Escherichia coli O55:H7 CB9615*	99	0.990	99	PICI	1444572	1455794	11.2	Same as *Escherichia coli O157:H7 EC4115*
		etw:ECSP_1496	*Escherichia coli*	*Escherichia coli O157:H7 TW14359*	99	0.990	99	PICI	1535786	1547005	11.2	Same as *Escherichia coli O157:H7 EC4115*
		eoh:ECO103_1241	*Escherichia coli*	*Escherichia coli O103:H2 12009*	99	0.980	99	PICI	1325875	1336965	11.1	Similar to *Escherichia coli O157:H7 EC4115*
		eoj:ECO26_1651	*Escherichia coli*	*Escherichia coli O26:H11 11368*	99	0.980	99	PICI	1619447	1630554	11.1	Same as *Escherichia coli O157:H7 EC4115*
		eoi:ECO111_2919	*Escherichia coli*	*Escherichia coli O111:H- 11128*	99	0.949	99	PICI	2855234	2864550	9.3	
		ecc:c1493	*Escherichia coli*	*Escherichia coli O6:K2:H1 CFT073*	99	0.919	99	PICI	1377749	1389210	11.4	
		sfe:SFxv_1290	*Shigella flexneri*	*Shigella flexneri 2002017*	99	0.818	99	PICI?	1232114	1246489	14.4	HNH; *ter*L
EC042_1759	*pri*	elo:EC042_1759	*Escherichia coli*	*Escherichia coli O44:H18 042*	466	1.000		EcCIO42				
		ecoh:ECRM13516_1446	*Escherichia coli*	*Escherichia coli O145:H28 RM13516*	466	0.998	466	PICI	1438775	1449995	11.2	Same as *Escherichia coli O157:H7 EC4115*
		ecoo:ECRM13514_1490	*Escherichia coli*	*Escherichia coli O145:H28 RM13514*	466	0.998	466	PICI	1462244	1473465	11.2	Same as *Escherichia coli O157:H7 EC4115*
		elr:ECO55CA74_06810	*Escherichia coli*	*Escherichia coli O55:H7 RM12579*	466	0.998	466	PICI	1438165	1449376	11.2	Same as *Escherichia coli O157:H7 EC4115*
		eoh:ECO103_1242	*Escherichia coli*	*Escherichia coli O103:H2 12009*	466	0.998	466	PICI	1325875	1336965	11.1	Similar to *Escherichia coli O157:H7 EC4115*
		eok:G2583_1396	*Escherichia coli*	*Escherichia coli O55:H7 CB9615*	466	0.998	466	PICI	1444572	1455794	11.2	Same as *Escherichia coli O157:H7 EC4115*
		ecf:ECH74115_1577	*Escherichia coli*	*Escherichia coli O157:H7 EC4115*	466	0.996	466	PICI	1535498	15466717	11.2	
		etw:ECSP_1497	*Escherichia coli*	*Escherichia coli O157:H7 TW14359*	466	0.996	466	PICI	1535786	1547005	11.2	Same as *Escherichia coli O157:H7 EC4115*
		eoj:ECO26_1652	*Escherichia coli*	*Escherichia coli O26:H11 11368*	466	0.996	466	PICI	1619447	1630554	11.1	Same as *Escherichia coli O157:H7 EC4115*
		sbo:SBO_2131	Shigella boydii	*Shigella boydii Sb227*	466	0.991	466	PICI	2097873	2108560	10.7	
		eoi:ECO111_2920	*Escherichia coli*	*Escherichia coli O111:H- 11128*	466	0.951	466	PICI	2855234	2864550	9.3	

EcCIO42 gene	Function	Orthologs	Species	Full name	Length	Similarity	Overlap	Element	Start	End	Size	Comments
		elc:i14_0250	*Escherichia coli*	*Escherichia coli clone D i14*	471	0.875	471	PICI	249047	259688	10.6	
		eld:i02_0250	*Escherichia coli*	*Escherichia coli clone D i2*	471	0.875	471	PICI	249047	259688	10.6	Same as *Escherichia coli clone D i14*
		enc:ECL_01694	*Enterobacter cloacae*	*Enterobacter cloacae subsp. cloacae ATCC 13047*	469	0.591	463	Prophage?				Chimera prophage & PICI
EC042_1758		elo:EC042_1758	*Escherichia coli*	*Escherichia coli O44:H18 042*	83	1.000		EcCIO42				
		ecf:ECH74115_1578	*Escherichia coli*	*Escherichia coli O157:H7 EC4115*	83	1.000	83	PICI	1535498	15466717	11.2	
		ecoh:ECRM13516_1447	*Escherichia coli*	*Escherichia coli O145:H28 RM13516*	83	1.000	83	PICI	1438775	1449995	11.2	Same as *Escherichia coli O157:H7 EC4115*
		ecoo:ECRM13514_1491	*Escherichia coli*	*Escherichia coli O145:H28 RM13514*	83	1.000	83	PICI	1462244	1473465	11.2	Same as *Escherichia coli O157:H7 EC4115*
		elr:ECO55CA74_06815	*Escherichia coli*	*Escherichia coli O55:H7 RM12579*	83	1.000	83	PICI	1438165	1449376	11.2	Same as *Escherichia coli O157:H7 EC4115*
		eoh:ECO103_1243	*Escherichia coli*	*Escherichia coli O103:H2 12009*	83	1.000	83	PICI	1325875	1336965	11.1	Similar to *Escherichia coli O157:H7 EC4115*
		eoj:ECO26_1653	*Escherichia coli*	*Escherichia coli O26:H11 11368*	83	1.000	83	PICI	1619447	1630554	11.1	Same as *Escherichia coli O157:H7 EC4115*
		eok:G2583_1397	*Escherichia coli*	*Escherichia coli O55:H7 CB9615*	83	1.000	83	PICI	1444572	1455794	11.2	Same as *Escherichia coli O157:H7 EC4115*
		etw:ECSP_1498	*Escherichia coli*	*Escherichia coli O157:H7 TW14359*	83	1.000	83	PICI	1535786	1547005	11.2	Same as *Escherichia coli O157:H7 EC4115*
		sbo:SBO_2129	*Shigella boydii*	*Shigella boydii Sb227*	93	1.000	83	PICI	2097873	2108560	10.7	
		ece:Z1844	*Escherichia coli*	*Escherichia coli O157:H7 EDL933*	83	0.915	82	PICI?	1678546	1694015	15.5	HNH; *ter*L
		ecs:ECs1586	*Escherichia coli*	*Escherichia coli O157:H7 Sakai*	83	0.915	82	PICI?	1594570	1610042	15.5	Same as *Escherichia coli O157:H7 EDL933*
		elx:CDCO157_1519	*Escherichia coli*	*Escherichia coli O157:H7 Xuzhou21*	83	0.915	82	PICI?	1596512	1611982	15.5	Same as *Escherichia coli O157:H7 EDL933*
		sfe:SFxv_1293	*Shigella flexneri*	*Shigella flexneri 2002017*	92	0.900	80	PICI?	1232114	1246489	14.4	HNH; *ter*L
		sfl:SF1136	*Shigella flexneri*	*Shigella flexneri 301*	92	0.900	80	PICI?	1174763	1189501	14.7	Similar to *Shigella flexneri 2002017*
		sft:NCTC1_01192	*Shigella flexneri*	*Shigella flexneri NCTC1*	92	0.900	80	PICI?	1133207	1147945	14.7	Similar to *Shigella flexneri 2002017*
		eoi:ECO111_2922	*Escherichia coli*	*Escherichia coli O111:H- 11128*	81	0.852	81	PICI	2855234	2864550	9.3	
		ecq:ECED1_1785	*Escherichia coli*	*Escherichia coli O81 ED1a*	92	0.808	78	PICI?	1739927	1756201	16.3	HNH; *ter*L
		ect:ECIAI39_2019	*Escherichia coli*	*Escherichia coli O7:K1 IAI39*	85	0.759	79	PICI?	2060161	2074688	14.5	HNH; *ter*L
EC042_1757	*ssb*	elo:EC042_1757	*Escherichia coli*	*Escherichia coli O44:H18 042*	136	1.000		EcCIO42				
		ecoh:ECRM13516_1448	*Escherichia coli*	*Escherichia coli O145:H28 RM13516*	136	1.000	136	PICI	1438775	1449995	11.2	Same as *Escherichia coli O157:H7 EC4115*
		ecoo:ECRM13514_1492	*Escherichia coli*	*Escherichia coli O145:H28 RM13514*	136	1.000	136	PICI	1462244	1473465	11.2	Same as *Escherichia coli O157:H7 EC4115*
		elr:ECO55CA74_06820	*Escherichia coli*	*Escherichia coli O55:H7 RM12579*	136	1.000	136	PICI	1438165	1449376	11.2	Same as *Escherichia coli O157:H7 EC4115*
		eoh:ECO103_1244	*Escherichia coli*	*Escherichia coli O103:H2 12009*	136	1.000	136	PICI	1325875	1336965	11.1	Similar to *Escherichia coli O157:H7 EC4115*
		eoj:ECO26_1654	*Escherichia coli*	*Escherichia coli O26:H11 11368*	136	1.000	136	PICI	1619447	1630554	11.1	Same as *Escherichia coli O157:H7 EC4115*
		eok:G2583_1398	*Escherichia coli*	*Escherichia coli O55:H7 CB9615*	136	1.000	136	PICI	1444572	1455794	11.2	Same as *Escherichia coli O157:H7 EC4115*
		sbo:SBO_2128	*Shigella boydii*	*Shigella boydii Sb227*	136	1.000	136	PICI	2097873	2108560	10.7	
		ecf:ECH74115_1579	*Escherichia coli*	*Escherichia coli O157:H7 EC4115*	136	0.993	136	PICI	1535498	15466717	11.2	
		etw:ECSP_1499	*Escherichia coli*	*Escherichia coli O157:H7 TW14359*	136	0.993	136	PICI	1535786	1547005	11.2	Same as *Escherichia coli O157:H7 EC4115*

EcClO42 gene	Function	Orthologs	Species	Full name	Length	Similarity	Overlap	Element	Start	End	Size	Comments
		ecc:c1497	*Escherichia coli*	*Escherichia coli O6:K2:H1 CFT073*	136	0.978	136	PICI	1377749	1389210	11.4	
		ecq:ECED1_1784	*Escherichia coli*	*Escherichia coli O81 ED1a*	136	0.978	136	PICI?	1739927	1756201	16.3	HNH; *ter*L
		ece:Z1845	*Escherichia coli*	*Escherichia coli O157:H7 EDL933*	136	0.978	136	PICI?	1678546	1694015	15.5	HNH; *ter*L
		ecs:ECs1587	*Escherichia coli*	*Escherichia coli O157:H7 Sakai*	136	0.978	136	PICI?	1594570	1610042	15.5	Same as *Escherichia coli O157:H7 EDL933*
		elx:CDCO157_1520	*Escherichia coli*	*Escherichia coli O157:H7 Xuzhou21*	136	0.978	136	PICI?	1596512	1611982	15.5	Same as *Escherichia coli O157:H7 EDL933*
		ect:ECIAI39_2018	*Escherichia coli*	*Escherichia coli O7:K1 IAI39*	136	0.831	136	PICI?	2060161	2074688	14.5	HNH; *ter*L
EC042_1756	*per*C	elo:EC042_1756	*Escherichia coli*	*Escherichia coli O44:H18 042*	90	1.000		EcClO42				
		ecoh:ECRM13516_1449	*Escherichia coli*	*Escherichia coli O145:H28 RM13516*	90	1.000	90	PICI	1438775	1449995	11.2	Same as *Escherichia coli O157:H7 EC4115*
		ecoo:ECRM13514_1493	*Escherichia coli*	*Escherichia coli O145:H28 RM13514*	90	1.000	90	PICI	1462244	1473465	11.2	Same as *Escherichia coli O157:H7 EC4115*
		elr:ECO55CA74_06825	*Escherichia coli*	*Escherichia coli O55:H7 RM12579*	90	1.000	90	PICI	1438165	1449376	11.2	Same as *Escherichia coli O157:H7 EC4115*
		eoh:ECO103_1245	*Escherichia coli*	*Escherichia coli O103:H2 12009*	90	1.000	90	PICI	1325875	1336965	11.1	Similar to *Escherichia coli O157:H7 EC4115*
		eoj:ECO26_1655	*Escherichia coli*	*Escherichia coli O26:H11 11368*	90	1.000	90	PICI	1619447	1630554	11.1	Same as *Escherichia coli O157:H7 EC4115*
		etw:ECSP_1500	*Escherichia coli*	*Escherichia coli O157:H7 TW14359*	90	1.000	90	PICI	1535786	1547005	11.2	Same as *Escherichia coli O157:H7 EC4115*
		ecs:ECs1588	*Escherichia coli*	*Escherichia coli O157:H7 Sakai*	90	0.900	90	PICI?	1594570	1610042	15.5	Same as *Escherichia coli O157:H7 EDL933*
		elx:CDCO157_1521	*Escherichia coli*	*Escherichia coli O157:H7 Xuzhou21*	90	0.900	90	PICI?	1596512	1611982	15.5	Same as *Escherichia coli O157:H7 EDL933*
		ecq:ECED1_1783	*Escherichia coli*	*Escherichia coli O81 ED1a*	122	0.844	77	PICI?	1739927	1756201	16.3	HNH; *ter*L
		eck:EC55989_0279	*Escherichia coli*	*Escherichia coli 55989*	95	0.489	88	PICI	296286	311648	15.3	
EC042_1755		elo:EC042_1755	*Escherichia coli*	*Escherichia coli O44:H18 042*	74	1.000		EcClO42				
		ecoh:ECRM13516_1451	*Escherichia coli*	*Escherichia coli O145:H28 RM13516*	74	1.000	74	PICI	1438775	1449995	11.2	Same as *Escherichia coli O157:H7 EC4115*
		ecoo:ECRM13514_1495	*Escherichia coli*	*Escherichia coli O145:H28 RM13514*	74	1.000	74	PICI	1462244	1473465	11.2	Same as *Escherichia coli O157:H7 EC4115*
		elr:ECO55CA74_06830	*Escherichia coli*	*Escherichia coli O55:H7 RM12579*	74	1.000	74	PICI	1438165	1449376	11.2	Same as *Escherichia coli O157:H7 EC4115*
		eoh:ECO103_1246	*Escherichia coli*	*Escherichia coli O103:H2 12009*	74	1.000	74	PICI	1325875	1336965	11.1	Similar to *Escherichia coli O157:H7 EC4115*
		eoj:ECO26_1656	*Escherichia coli*	*Escherichia coli O26:H11 11368*	74	1.000	74	PICI	1619447	1630554	11.1	Same as *Escherichia coli O157:H7 EC4115*
		etw:ECSP_1501	*Escherichia coli*	*Escherichia coli O157:H7 TW14359*	74	1.000	74	PICI	1535786	1547005	11.2	Same as *Escherichia coli O157:H7 EC4115*
		sbo:SBO_2127	*Shigella boydii*	*Shigella boydii Sb227*	74	1.000	74	PICI	2097873	2108560	10.7	
		ece:Z1846	*Escherichia coli*	*Escherichia coli O157:H7 EDL933*	100	0.986	74	PICI?	1678546	1694015	15.5	HNH; *ter*L
		ecs:ECs1589	*Escherichia coli*	*Escherichia coli O157:H7 Sakai*	100	0.986	74	PICI?	1594570	1610042	15.5	Same as *Escherichia coli O157:H7 EDL933*
		elx:CDCO157_1522	*Escherichia coli*	*Escherichia coli O157:H7 Xuzhou21*	100	0.986	74	PICI?	1596512	1611982	15.5	Same as *Escherichia coli O157:H7 EDL933*
		ecq:ECED1_1781	*Escherichia coli*	*Escherichia coli O81 ED1a*	100	0.932	74	PICI?	1739927	1756201	16.3	HNH; *ter*L
		sfl:SF1137	*Shigella flexneri*	*Shigella flexneri 301*	100	0.932	74	PICI?	1174763	1189501	14.7	Similar to *Shigella flexneri 2002017*
		sft:NCTC1_01195	*Shigella flexneri*	*Shigella flexneri NCTC1*	74	0.932	74	PICI?	1133207	1147945	14.7	Similar to *Shigella flexneri 2002017*
		sfv:SFV_1153	*Shigella flexneri*	*Shigella flexneri 8401*	100	0.932	74	PICI?	1192843	1208358	15.5	Similar to *Shigella flexneri 2002017*

EcClO42 gene	Function	Orthologs	Species	Full name	Length	Similarity	Overlap	Element	Start	End	Size	Comments
		sfx:S1219	*Shigella flexneri*	*Shigella flexneri 2457T*	74	0.932	74	PICI?	1177656	1192394	14.7	Similar to *Shigella flexneri 2002017*
		ecm:EcSMS35_0949	*Escherichia coli*	*Escherichia coli SMS-3-5*	80	0.444	72	PICI?	955625	970780	15.2	HNH; *ter*L
EC042_1754		elo:EC042_1754	*Escherichia coli*	*Escherichia coli O44:H18 042*	79	1.000		EcClO42				
		sbo:SBO_2126	*Shigella boydii*	*Shigella boydii Sb227*	79	1.000	79	PICI	2097873	2108560	10.7	
		ecoi:ECOPMV1_00893	*Escherichia coli*	*Escherichia coli PMV-1*	68	1.000	68	PICI	919885	930438	10.5	
		eih:ECOK1_0892	*Escherichia coli*	*Escherichia coli IHE3034*	68	1.000	68	PICI	920272	930825	10.5	Same as *Escherichia coli PMV-1*
		elr:ECO55CA74_06835	*Escherichia coli*	*Escherichia coli O55:H7 RM12579*	68	1.000	68	PICI	1438165	1449376	11.2	Same as *Escherichia coli O157:H7 EC4115*
		elu:UM146_13195	*Escherichia coli*	*Escherichia coli UM146*	68	1.000	68	PICI	2721434	2731984	10.5	Same as *Escherichia coli PMV-1*
		eok:G2583_1399	*Escherichia coli*	*Escherichia coli O55:H7 CB9615*	68	1.000	68	PICI	1444572	1455794	11.2	Same as *Escherichia coli O157:H7 EC4115*
		ecf:ECH74115_1582	*Escherichia coli*	*Escherichia coli O157:H7 EC4115*	68	0.985	68	PICI	1535498	15466717	11.2	
		etw:ECSP_1502	*Escherichia coli*	*Escherichia coli O157:H7 TW14359*	68	0.985	68	PICI	1535786	1547005	11.2	Same as *Escherichia coli O157:H7 EC4115*
		eoh:ECO103_1247	*Escherichia coli*	*Escherichia coli O103:H2 12009*	68	0.691	68	PICI	1325875	1336965	11.1	Similar to *Escherichia coli O157:H7 EC4115*
		ecoh:ECRM13516_1452	*Escherichia coli*	*Escherichia coli O145:H28 RM13516*	68	0.691	68	PICI	1438775	1449995	11.2	Same as *Escherichia coli O157:H7 EC4115*
EC042_1753	capsid	elo:EC042_1753	*Escherichia coli*	*Escherichia coli O44:H18 042*	351	1.000		EcClO42				
		ecf:ECH74115_1583	*Escherichia coli*	*Escherichia coli O157:H7 EC4115*	351	1.000	351	PICI	1535498	15466717	11.2	
		elr:ECO55CA74_06840	*Escherichia coli*	*Escherichia coli O55:H7 RM12579*	351	1.000	351	PICI	1438165	1449376	11.2	Same as *Escherichia coli O157:H7 EC4115*
		eok:G2583_1400	*Escherichia coli*	*Escherichia coli O55:H7 CB9615*	351	1.000	351	PICI	1444572	1455794	11.2	Same as *Escherichia coli O157:H7 EC4115*
		etw:ECSP_1503	*Escherichia coli*	*Escherichia coli O157:H7 TW14359*	351	1.000	351	PICI	1535786	1547005	11.2	Same as *Escherichia coli O157:H7 EC4115*
		sbo:SBO_2125	*Shigella boydii*	*Shigella boydii Sb227*	351	0.972	351	PICI	2097873	2108560	10.7	
		ecoh:ECRM13516_1453	*Escherichia coli*	*Escherichia coli O145:H28 RM13516*	351	0.960	351	PICI	1438775	1449995	11.2	Same as *Escherichia coli O157:H7 EC4115*
		eoh:ECO103_1248	*Escherichia coli*	*Escherichia coli O103:H2 12009*	351	0.960	351	PICI	1325875	1336965	11.1	Similar to *Escherichia coli O157:H7 EC4115*
		eoj:ECO26_1658	*Escherichia coli*	*Escherichia coli O26:H11 11368*	351	0.960	351	PICI	1619447	1630554	11.1	Same as *Escherichia coli O157:H7 EC4115*
		ecoo:ECRM13514_1497	*Escherichia coli*	*Escherichia coli O145:H28 RM13514*	351	0.957	351	PICI	1462244	1473465	11.2	Same as *Escherichia coli O157:H7 EC4115*
		ecc:c1499	*Escherichia coli*	*Escherichia coli O6:K2:H1 CFT073*	351	0.969	350	PICI	1377749	1389210	11.4	
		ecoi:ECOPMV1_00894	*Escherichia coli*	*Escherichia coli PMV-1*	351	0.954	350	PICI	919885	930438	10.5	
		eih:ECOK1_0893	*Escherichia coli*	*Escherichia coli IHE3034*	351	0.954	350	PICI	920272	930825	10.5	Same as *Escherichia coli PMV-1*
		elu:UM146_13190	*Escherichia coli*	*Escherichia coli UM146*	351	0.954	350	PICI	2721434	2731984	10.5	Same as *Escherichia coli PMV-1*
		ecq:ECED1_2651	*Escherichia coli*	*Escherichia coli O81 ED1a*	351	0.937	351	PICI	2568989	2580418	11.4	
		elc:i14_0254	*Escherichia coli*	*Escherichia coli clone D i14*	351	0.934	350	PICI	249047	259688	10.6	
		eld:i02_0254	*Escherichia coli*	*Escherichia coli clone D i2*	351	0.934	350	PICI	249047	259688	10.6	Same as *Escherichia coli clone D i14*
		eln:NRG857_05760	*Escherichia coli*	*Escherichia coli O83:H1 NRG 857C*	346	0.547	340	Prophage?				Chimera prophage & PICI

EcCIO42 gene	Function	Orthologs	Species	Full name	Length	Similarity	Overlap	Element	Start	End	Size	Comments
EC042_1752	HDHD	elo:EC042_1752	*Escherichia coli*	*Escherichia coli O44:H18 042*	111	1.000		EcCIO42				
		ecf:ECH74115_1584	*Escherichia coli*	*Escherichia coli O157:H7 EC4115*	111	1.000	111	PICI	1535498	15466717	11.2	
		elr:ECO55CA74_06845	*Escherichia coli*	*Escherichia coli O55:H7 RM12579*	111	1.000	111	PICI	1438165	1449376	11.2	Same as *Escherichia coli O157:H7 EC4115*
		eok:G2583_1401	*Escherichia coli*	*Escherichia coli O55:H7 CB9615*	111	1.000	111	PICI	1444572	1455794	11.2	Same as *Escherichia coli O157:H7 EC4115*
		etw:ECSP_1504	*Escherichia coli*	*Escherichia coli O157:H7 TW14359*	111	1.000	111	PICI	1535786	1547005	11.2	Same as *Escherichia coli O157:H7 EC4115*
		ecoi:ECOPMV1_00895	*Escherichia coli*	*Escherichia coli PMV-1*	111	0.991	111	PICI	919885	930438	10.5	
		eih:ECOK1_0894	*Escherichia coli*	*Escherichia coli IHE3034*	111	0.991	111	PICI	920272	930825	10.5	Same as *Escherichia coli PMV-1*
		elu:UM146_13185	*Escherichia coli*	*Escherichia coli UM146*	111	0.991	111	PICI	2721434	2731984	10.5	Same as *Escherichia coli PMV-1*
		ecq:ECED1_2652	*Escherichia coli*	*Escherichia coli O81 ED1a*	111	0.991	111	PICI	2568989	2580418	11.4	
		elc:i14_0255	*Escherichia coli*	*Escherichia coli clone D i14*	111	0.991	111	PICI	249047	259688	10.6	
		eld:i02_0255	*Escherichia coli*	*Escherichia coli clone D i2*	111	0.991	111	PICI	249047	259688	10.6	Same as *Escherichia coli clone D i14*
		ecc:c1500	*Escherichia coli*	*Escherichia coli O6:K2:H1 CFT073*	111	0.982	111	PICI	1377749	1389210	11.4	
		ecoh:ECRM13516_1454	*Escherichia coli*	*Escherichia coli O145:H28 RM13516*	111	0.973	111	PICI	1438775	1449995	11.2	Same as *Escherichia coli O157:H7 EC4115*
		ecoo:ECRM13514_1498	*Escherichia coli*	*Escherichia coli O145:H28 RM13514*	111	0.973	111	PICI	1462244	1473465	11.2	Same as *Escherichia coli O157:H7 EC4115*
		eoh:ECO103_1249	*Escherichia coli*	*Escherichia coli O103:H2 12009*	111	0.973	111	PICI	1325875	1336965	11.1	Similar to *Escherichia coli O157:H7 EC4115*
		eoj:ECO26_1659	*Escherichia coli*	*Escherichia coli O26:H11 11368*	111	0.973	111	PICI	1619447	1630554	11.1	Same as *Escherichia coli O157:H7 EC4115*
		sbo:SBO_2124	*Shigella boydii*	*Shigella boydii Sb227*	111	0.982	111	PICI	2097873	2108560	10.7	
		ect:ECIAI39_0514	*Escherichia coli*	*Escherichia coli O7:K1 IAI39*	113	0.545	112	Prophage?				Chimera prophage & PICI
EC042_1751		elo:EC042_1751	*Escherichia coli*	*Escherichia coli O44:H18 042*	130	1.000		EcCIO42				
		eok:G2583_1402	*Escherichia coli*	*Escherichia coli O55:H7 CB9615*	137	0.985	130	PICI	1444572	1455794	11.2	Same as *Escherichia coli O157:H7 EC4115*
		elr:ECO55CA74_06850	*Escherichia coli*	*Escherichia coli O55:H7 RM12579*	137	0.977	130	PICI	1438165	1449376	11.2	Same as *Escherichia coli O157:H7 EC4115*
		ecf:ECH74115_1585	*Escherichia coli*	*Escherichia coli O157:H7 EC4115*	137	0.977	130	PICI	1535498	15466717	11.2	
		etw:ECSP_1505	*Escherichia coli*	*Escherichia coli O157:H7 TW14359*	137	0.977	130	PICI	1535786	1547005	11.2	Same as *Escherichia coli O157:H7 EC4115*
		ecc:c1501	*Escherichia coli*	*Escherichia coli O6:K2:H1 CFT073*	137	0.954	130	PICI	1377749	1389210	11.4	
		ecoi:ECOPMV1_00896	*Escherichia coli*	*Escherichia coli PMV-1*	137	0.946	130	PICI	919885	930438	10.5	
		eih:ECOK1_0895	*Escherichia coli*	*Escherichia coli IHE3034*	137	0.946	130	PICI	920272	930825	10.5	Same as *Escherichia coli PMV-1*
		elu:UM146_13180	*Escherichia coli*	*Escherichia coli UM146*	137	0.946	130	PICI	2721434	2731984	10.5	Same as *Escherichia coli PMV-1*
		sbo:SBO_2123	*Shigella boydii*	*Shigella boydii Sb227*	137	0.931	130	PICI	2097873	2108560	10.7	
		ecq:ECED1_2653	*Escherichia coli*	*Escherichia coli O81 ED1a*	137	0.915	130	PICI	2568989	2580418	11.4	
		ecoh:ECRM13516_1455	*Escherichia coli*	*Escherichia coli O145:H28 RM13516*	137	0.915	130	PICI	1438775	1449995	11.2	Same as *Escherichia coli O157:H7 EC4115*
		ecoo:ECRM13514_1499	*Escherichia coli*	*Escherichia coli O145:H28 RM13514*	137	0.915	130	PICI	1462244	1473465	11.2	Same as *Escherichia coli O157:H7 EC4115*
		eoh:ECO103_1250	*Escherichia coli*	*Escherichia coli O103:H2 12009*	137	0.915	130	PICI	1325875	1336965	11.1	Similar to *Escherichia coli O157:H7 EC4115*
		eoj:ECO26_1660	*Escherichia coli*	*Escherichia coli O26:H11 11368*	137	0.915	130	PICI	1619447	1630554	11.1	Same as *Escherichia coli O157:H7 EC4115*

EcCIO42 gene	Function	Orthologs	Species	Full name	Length	Similarity	Overlap	Element	Start	End	Size	Comments
		elc:i14_0256	*Escherichia coli*	*Escherichia coli clone D i14*	137	0.885	130	PICI	249047	259688	10.6	
		eld:i02_0256	*Escherichia coli*	*Escherichia coli clone D i2*	137	0.885	130	PICI	249047	259688	10.6	Same as *Escherichia coli clone D i14*
		eoc:CE10_1475	*Escherichia coli*	*Escherichia coli O7:K1 CE10*	501	0.386	83	Phage				
EC042_1750		elo:EC042_1750	*Escherichia coli*	*Escherichia coli O44:H18 042*	113	1.000		EcCIO42				
		ecc:c1503	*Escherichia coli*	*Escherichia coli O6:K2:H1 CFT073*	113	0.982	113	PICI	1377749	1389210	11.4	
		sbo:SBO_2121	*Shigella boydii*	*Shigella boydii Sb227*	113	0.982	113	PICI	2097873	2108560	10.7	
		aaw:AVL56_03045	*Alteromonas addita*	*Alteromonas addita R10SW13*	774	0.340	106	No insert				
		asp:AOR13_2906	*Alteromonas stellipolaris*	*Alteromonas stellipolaris LMG 21856*	774	0.340	106	No insert				
		asq:AVL57_04270	*Alteromonas stellipolaris*	*Alteromonas stellipolaris LMG 21861*	774	0.340	106	No insert				
		pte:PTT_18114	*Pyrenophora teres*	*Pyrenophora teres f. teres 0-1*	380	0.318	88					RefSeq (Assembly: GCF_000166005.1)
		tru:101068395	*Takifugu rubripes*	*Takifugu rubripes*	1244	0.312	96					RefSeq (Assembly: GCF_000180615.1)
		miu:ABE85_20515	*Mitsuaria sp. 7*	*Mitsuaria sp. 7*	285	0.322	87	No insert				
		spaa:SPAPADRAFT_153792	*Spathaspora passalidarum*	*Spathaspora passalidarum NRRL Y-27907*	711	0.309	81					RefSeq (Assembly: GCF_000223485.1)
		ang:ANI_1_2260104	*Aspergillus niger*	*Aspergillus niger CBS 513.88*	412	0.354	113					RefSeq (Assembly: GCF_000002855.3)
EC042_1749		elo:EC042_1749	*Escherichia coli*	*Escherichia coli O44:H18 042*	93	1.000		EcCIO42				
		eih:ECOK1_0897	*Escherichia coli*	*Escherichia coli IHE3034*	93	0.957	93	PICI	920272	930825	10.5	Same as *Escherichia coli PMV-1*
		sbo:SBO_2120	*Shigella boydii*	*Shigella boydii Sb227*	93	0.957	93	PICI	2097873	2108560	10.7	
		ecf:ECH74115_1588	*Escherichia coli*	*Escherichia coli O157:H7 EC4115*	93	0.925	93	PICI	1535498	15466717	11.2	
		ecoh:ECRM13516_1457	*Escherichia coli*	*Escherichia coli O145:H28 RM13516*	93	0.925	93	PICI	1438775	1449995	11.2	Same as *Escherichia coli O157:H7 EC4115*
		ecoo:ECRM13514_1501	*Escherichia coli*	*Escherichia coli O145:H28 RM13514*	93	0.925	93	PICI	1462244	1473465	11.2	Same as *Escherichia coli O157:H7 EC4115*
		elr:ECO55CA74_06860	*Escherichia coli*	*Escherichia coli O55:H7 RM12579*	93	0.925	93	PICI	1438165	1449376	11.2	Same as *Escherichia coli O157:H7 EC4115*
		eoh:ECO103_1252	*Escherichia coli*	*Escherichia coli O103:H2 12009*	93	0.925	93	PICI	1325875	1336965	11.1	Similar to *Escherichia coli O157:H7 EC4115*
		eoj:ECO26_1662	*Escherichia coli*	*Escherichia coli O26:H11 11368*	93	0.925	93	PICI	1619447	1630554	11.1	Same as *Escherichia coli O157:H7 EC4115*
		eok:G2583_1404	*Escherichia coli*	*Escherichia coli O55:H7 CB9615*	93	0.925	93	PICI	1444572	1455794	11.2	Same as *Escherichia coli O157:H7 EC4115*
		etw:ECSP_1507	*Escherichia coli*	*Escherichia coli O157:H7 TW14359*	93	0.925	93	PICI	1535786	1547005	11.2	Same as *Escherichia coli O157:H7 EC4115*
		ece:Z1855	*Escherichia coli*	*Escherichia coli O157:H7 EDL933*	93	0.925	93	PICI?	1678546	1694015	15.5	HNH; *ter*L
		ecs:ECs1599	*Escherichia coli*	*Escherichia coli O157:H7 Sakai*	93	0.925	93	PICI?	1594570	1610042	15.5	Same as *Escherichia coli O157:H7 EDL933*
		elx:CDCO157_1532	*Escherichia coli*	*Escherichia coli O157:H7 Xuzhou21*	93	0.925	93	PICI?	1596512	1611982	15.5	Same as *Escherichia coli O157:H7 EDL933*
		ecc:c1505	*Escherichia coli*	*Escherichia coli O6:K2:H1 CFT073*	93	0.914	93	PICI	1377749	1389210	11.4	
		sfe:SFxv_1307	*Shigella flexneri*	*Shigella flexneri 2002017*	93	0.903	93	PICI?	1232114	1246489	14.4	HNH; *ter*L

Table S4. PmCIATCC43137 orthologies.

PmCIATCC 43137	Function	Orthologs	Species	Full name	Length	Similarity	Overlap	Element	Start	End	Size	Comments
DR93_170	*int*	pmul:DR93_170	*Pasteurella multocida*	*Pasteurella multocida ATCC 43137*	427	1.000		PmCIATCC 43137				
		pmp:Pmu_15680	*Pasteurella multocida*	*Pasteurella multocida 36950*	427	0.998	427	PmCIATCC 43137	1677145	1691873	14.7	
		pul:NT08PM_1626	*Pasteurella multocida*	*Pasteurella multocida subsp. multocida 3480*	427	0.965	427	PICI	1690704	1703854	13.1	
		pmu:PM1769	*Pasteurella multocida*	*Pasteurella multocida subsp. multocida Pm70*	417	0.962	417	PICI	1994540	2009007	14.5	Similar to PmCIPm3480
		hap:HAPS_1587	*Haemophilus parasuis*	*Haemophilus parasuis SH0165*	416	0.644	417	PICI?	1557480	1565531	8	Not commun structure
		aah:CF65_01449	*Aggregatibacter actinomycetemcomitans*	*Aggregatibacter actinomycetemcomitans HK1651*	307	0.639	277	No insert				
		mhae:F382_05840	*Mannheimia haemolytica*	*Mannheimia haemolytica D153*	408	0.447	425	Defective PICI?				
		mhal:N220_11990	*Mannheimia haemolytica*	*Mannheimia haemolytica USMARC_2286*	408	0.447	425	Defective PICI?				Same as *Mannheimia haemolytica D153*
		mhao:J451_06080	*Mannheimia haemolytica*	*Mannheimia haemolytica D174*	408	0.447	425	Defective PICI?				Same as *Mannheimia haemolytica D153*
		mhaq:WC39_02240	*Mannheimia haemolytica*	*Mannheimia haemolytica 89010807N*	408	0.447	425	Defective PICI?				Same as *Mannheimia haemolytica D153*
		mhay:VK67_02245	*Mannheimia haemolytica*	*Mannheimia haemolytica 89010807N lktA-*	408	0.447	425	Defective PICI?				Same as *Mannheimia haemolytica D153*
DR93_169		pmul:DR93_169	*Pasteurella multocida*	*Pasteurella multocida ATCC 43137*	201	1.000		PmCIATCC 43137				
		pmp:Pmu_15680	*Pasteurella multocida*	*Pasteurella multocida 36950*	201	1.000	201	PmCIATCC 43137	1677145	1691873	14.7	
		aap:NT05HA_1837	*Aggregatibacter aphrophilus*	*Aggregatibacter aphrophilus NJ8700*	167	0.310	142	PICI	1811901	1821358	9.4	
		bip:Bint_1433	*Brachyspira intermedia*	*Brachyspira intermedia*	7866	0.266	154	No insert				Plasmid
		arc:ABLL_0889	*Arcobacter sp. L*	*Arcobacter sp. L*	744	0.308	107	No insert				Plasmid
		stur:STURON_00280	*Spiroplasma turonicum*	*Spiroplasma turonicum Tab4c*	638	0.320	128	No insert				
		kga:ST1E_0968	*Candidatus Kinetoplastibacterium galatii*	*Candidatus Kinetoplastibacterium galatii TCC219*	295	0.316	79	No insert				
		cbv:U729_1798	*Clostridium baratii*	*Clostridium baratii Sullivan*	205	0.302	116	No insert				Plasmid
		gmx:100798343	*Glycine max (soybean)*	*Glycine max (soybean)*	345	0.330	112					RefSeq (Assembly: GCF_000004515.4)
		bvt:P613_03425	*Borreliella valaisiana*	*Borreliella valaisiana Tom4006*	466	0.333	90	No insert				
		cdg:CDBI1_18763	*Clostridioides difficile*	*Clostridioides difficile BI1*	333	0.305	174	No insert				
DR93_168	*mer*R	pmul:DR93_168	*Pasteurella multocida*	*Pasteurella multocida ATCC 43137*	65	1.000		PmCIATCC 43137				
		pmp:Pmu_15660	*Pasteurella multocida*	*Pasteurella multocida 36950*	65	1.000	65	PmCIATCC 43137	1677145	1691873	14.7	
		pmu:PM1772	*Pasteurella multocida*	*Pasteurella multocida subsp. multocida Pm70*	65	1.000	65	PICI	1994540	2009007	14.5	Similar to PmCIPm3480
		pul:NT08PM_1624	*Pasteurella multocida*	*Pasteurella multocida subsp. multocida 3480*	65	0.985	65	PICI	1690704	1703854	13.1	
		aap:NT05HA_2319	*Aggregatibacter aphrophilus*	*Aggregatibacter aphrophilus NJ8700*	63	0.390	59	PICI	1811901	1821358	9.4	
		asu:Asuc_1205	*Actinobacillus succinogenes*	*Actinobacillus succinogenes 130Z*	61	0.393	56	No insert				
		emu:EMQU_1667	*Enterococcus mundtii*	*Enterococcus mundtii QU 25*	152	0.385	52	No insert				

PmCIATCC 43137	Function	Orthologs	Species	Full name	Length	Similarity	Overlap	Element	Start	End	Size	Comments
		tin:Tint_1645	*Thiomonas intermedia*	*Thiomonas intermedia K12*	73	0.339	62	No insert				
		dly:Dehly_1676	*Dehalogenimonas lykanthroporepellens*	*Dehalogenimonas lykanthroporepellens BL-DC-9*	119	0.351	57	Prophage				
		thi:THI_3204	*Thiomonas arsenitoxydans*	*Thiomonas arsenitoxydans*	73	0.323	62	No insert				
		gsb:GSUB_10080	*Geoalkalibacter subterraneus*	*Geoalkalibacter subterraneus Red1*	69	0.377	53	Prophage				
DR93_167		pmul:DR93_167	*Pasteurella multocida*	*Pasteurella multocida ATCC 43137*	140	1.000		PmCIATCC 43137				
		pmp:Pmu_15650	*Pasteurella multocida*	*Pasteurella multocida 36950*	140	1.000	140	PmCIATCC 43137	1677145	1691873	14.7	
		pmu:PM1773	*Pasteurella multocida*	*Pasteurella multocida subsp. multocida Pm70*	140	0.979	140	PICI	1994540	2009007	14.5	Similar to PmCIPm3480
		pul:NT08PM_1623	*Pasteurella multocida*	*Pasteurella multocida subsp. multocida 3480*	140	0.964	85	PICI	1690704	1703854	13.1	
		etc:ETAC_13005	*Edwardsiella piscicida*	*Edwardsiella piscicida C07-087*	144	0.329	85	PICI	2913709	2929995	16.3	
		pge:LG71_03425	*Pluralibacter gergoviae*	*Pluralibacter gergoviae FB2*	144	0.329	92	Prophage				Tail protein
		yef:FORC2_0765	*Yersinia enterocolitica*	*Yersinia enterocolitica FORC_002*	129	0.315	85	PICI?	869225	883876	14.6	Tail protein
		pec:W5S_3563	*Pectobacterium*	*Pectobacterium sp. SCC3193*	143	0.306	85	Defective PICI				
		senc:SEET0819_22135	*Salmonella enterica subsp. enterica*	*Salmonella enterica subsp. enterica serovar Tennessee TXSC_TXSC08-19*	144	0.318	85	PICI?	4601635	4620336	18.7	Tail protein
		sbc:SbBS512_E4075	*Shigella boydii*	*Shigella boydii CDC 3083-94*	143	0.318	85	Defective PICI	3800221	3813466	13	Transposases
		eta:ETA_24300	*Erwinia tasmaniensis*	*Erwinia tasmaniensis Et1/99*	138	0.306	85	PICI?	2728486	2741458	13	Tail protein
DR93_166	Rha	pmul:DR93_166	*Pasteurella multocida*	*Pasteurella multocida ATCC 43137*	239	1.000		PmCIATCC 43137				
		pmp:Pmu_15640	*Pasteurella multocida*	*Pasteurella multocida 36950*	239	1.000	239	PmCIATCC 43137	1677145	1691873	14.7	
		pul:NT08PM_1622	*Pasteurella multocida*	*Pasteurella multocida subsp. multocida 3480*	239	0.912	239	PICI	1690704	1703854	13.1	
		pmu:PM1774	*Pasteurella multocida*	*Pasteurella multocida subsp. multocida Pm70*	239	0.883	239	PICI	1994540	2009007	14.5	Similar to PmCIPm3480
		mhat:B824_10850	*Mannheimia haemolytica*	*Mannheimia haemolytica USDA-ARS-USMARC-184*	222	0.668	214	PICI	1058301	1066853	8.5	
		hia:H733_0898	*Haemophilus influenzae*	*Haemophilus influenzae CGSHiCZ412602*	227	0.656	212	Defective PICI				
		aeu:ACEE_02550	*Actinobacillus equuli*	*Actinobacillus equuli subsp. equuli 19392*	233	0.650	214	PICI?	557158	568179	11	Not commun structure
		asi:ASU2_00405	*Actinobacillus suis*	*Actinobacillus suis H91-0380*	233	0.645	214	PICI?	73077	80675	7.6	Not commun structure
		ass:ASU1_00410	*Actinobacillus suis*	*Actinobacillus suis ATCC 33415*	233	0.645	214	PICI?	73071	82984	9.9	Not commun structure
		mham:J450_11860	*Mannheimia haemolytica*	*Mannheimia haemolytica D171*	202	0.668	199	PICI?	2445215	2453749	8.5	Same as *Mannheimia haemolytica USDA-ARS-USMARC-184*
		aacn:AANUM_0892	*Aggregatibacter actinomycetemcomitans*	*Aggregatibacter actinomycetemcomitans NUM4039*	228	0.537	205	PICI?	855426	865368	9.9	No primase
DR93_165		pmul:DR93_165	*Pasteurella multocida*	*Pasteurella multocida ATCC 43137*	92	1.000		PmCIATCC 43137				
		pmp:Pmu_15630	*Pasteurella multocida*	*Pasteurella multocida 36950*	92	1.000	92	PmCIATCC 43137	1677145	1691873	14.7	
		pmu:PM1775	*Pasteurella multocida*	*Pasteurella multocida subsp. multocida Pm70*	92	0.935	92	PICI	1994540	2009007	14.5	Similar to PmCIPm3480

PmCIATCC 43137	Function	Orthologs	Species	Full name	Length	Similarity	Overlap	Element	Start	End	Size	Comments
		pul:NT08PM_1621	*Pasteurella multocida*	*Pasteurella multocida subsp. multocida 3480*	95	0.925	93	PICI	1690704	1703854	13.1	
		swd:Swoo_3850	*Shewanella woodyi*	*Shewanella woodyi ATCC 51908*	470	0.306	85	No insert				
		tva:TVAG_244940	*Trichomonas vaginalis*	*Trichomonas vaginalis G3*	2416	0.302	86					RefSeq (Assembly: GCF_000002825.2)
		pjd:Pjdr2_4470	*Paenibacillus*	*Paenibacillus sp. JDR-2*	721	0.339	56	No insert				
		cbv:U729_1200	*Clostridium baratii*	*Clostridium baratii Sullivan*	218	0.381	42	No insert				
		tch:CHITON_0004	*Thermococcus chitonophagus*	*Thermococcus chitonophagus 1*	589	0.333	84	No insert				
		bvg:104901537	*Beta vulgaris*	*Beta vulgaris subsp. vulgaris (sugar beet)*	853	0.321	56					RefSeq (Assembly: GCF_000511025.1)
		riv:Riv7116_2425	*Rivularia*	*Rivularia sp. PCC 7116*	563	0.462	39	No insert				
DR93_164		pmul:DR93_164	*Pasteurella multocida*	*Pasteurella multocida ATCC 43137*	91	1.000		PmCIATCC 43137				
		pmp:Pmu_15610	*Pasteurella multocida*	*Pasteurella multocida 36950*	95	1.000	91	PmCIATCC 43137	1677145	1691873	14.7	
		pmu:PM1776	*Pasteurella multocida*	*Pasteurella multocida subsp. multocida Pm70*	95	0.945	91	PICI	1994540	2009007	14.5	Similar to PmCIPm3480
		aan:D7S_01414	*Aggregatibacter actinomycetemcomitans*	*Aggregatibacter actinomycetemcomitans D7S-1*	91	0.371	89	PICI	1321056	1330241	9.2	
		mvr:X781_11180	*Mannheimia sp.*	*Mannheimia sp. USDA-ARS-USMARC-1261*	87	0.429	91	PICI?	1128249	1143315	15	Not commun structure; *ter*L
		hif:HIBPF_06732	*Haemophilus influenzae*	*Haemophilus influenzae F3031*	98	0.323	96	PICI?	617232	628294	11	Not commun structure
		hil:HICON_15220	*Haemophilus influenzae*	*Haemophilus influenzae F3047*	100	0.313	99	PICI?	1359833	1373077	13.2	Not commun structure; similar to *H.influenzae* F3031
		hiu:HIB_05670	*Haemophilus influenzae*	*Haemophilus influenzae 10810*	94	0.333	93	PICI?	563371	577256	13.8	Not commun structure
		mham:J450_11855	*Mannheimia haemolytica*	*Mannheimia haemolytica D171*	95	0.310	84	PICI?	2445215	2453749	8.5	Same as *Mannheimia haemolytica USDA-ARS-USMARC-184*
		mhat:B824_10840	*Mannheimia haemolytica*	*Mannheimia haemolytica USDA-ARS-USMARC-184*	83	0.312	80	PICI	1058301	1066853	8.5	
		aqu:105314043	*Amphimedon*	*Amphimedon queenslandica*	436	0.347	49					RefSeq (Assembly: GCF_000090795.1)
DR93_163		pmul:DR93_163	*Pasteurella multocida*	*Pasteurella multocida ATCC 43137*	120	1.000		PmCIATCC 43137				
		pmp:Pmu_15600	*Pasteurella multocida*	*Pasteurella multocida 36950*	120	1.000	120	PmCIATCC 43137	1677145	1691873	14.7	
		pul:NT08PM_1619	*Pasteurella multocida*	*Pasteurella multocida subsp. multocida 3480*	120	1.000	120	PICI	1690704	1703854	13.1	
		aacn:AANUM_0889	*Aggregatibacter actinomycetemcomitans*	*Aggregatibacter actinomycetemcomitans NUM4039*	124	0.646	99	PICI?	855426	865368	9.9	No primase
		hia:H733_0899	*Haemophilus influenzae*	*Haemophilus influenzae CGSHiCZ412602*	124	0.616	99	PICI?	972960	980869	7.9	
		aan:D7S_01415	*Aggregatibacter actinomycetemcomitans*	*Aggregatibacter actinomycetemcomitans D7S-1*	127	0.555	110	PICI	1321056	1330241	9.2	
		aap:NT05HA_1832	*Aggregatibacter aphrophilus*	*Aggregatibacter aphrophilus NJ8700*	127	0.555	110	PICI	1811901	1821358	9.4	
		aeu:ACEE_02555	*Actinobacillus equuli*	*Actinobacillus equuli subsp. equuli 19392*	127	0.492	124	PICI?	557158	568179	11	Not commun structure
		asi:ASU2_00400	*Actinobacillus suis*	*Actinobacillus suis H91-0380*	127	0.480	125	PICI?	73077	80675	7.6	Not commun structure
		ass:ASU1_00405	*Actinobacillus suis*	*Actinobacillus suis ATCC 33415*	127	0.480	125	PICI?	73071	82984	9.9	Not commun structure
		kpb:FH42_07375	*Klebsiella pneumoniae*	*Klebsiella pneumoniae blaNDM-1*	934	0.324	102	No insert				

PmCIATCC 43137	Function	Orthologs	Species	Full name	Length	Similarity	Overlap	Element	Start	End	Size	Comments
DR93_162	*ash*	pmul:DR93_162	*Pasteurella multocida*	*Pasteurella multocida ATCC 43137*	186	1.000		PmCIATCC 43137				
		pmp:Pmu_15590	*Pasteurella multocida*	*Pasteurella multocida 36950*	186	1.000	186	PmCIATCC 43137	1677145	1691873	14.7	
		pul:NT08PM_1618	*Pasteurella multocida*	*Pasteurella multocida subsp. multocida 3480*	186	1.000	186	PICI	1690704	1703854	13.1	
		pmu:PM1778	*Pasteurella multocida*	*Pasteurella multocida subsp. multocida Pm70*	186	0.962	186	PICI	1994540	2009007	14.5	Similar to PmCIPm3480
		aap:NT05HA_2318	*Aggregatibacter aphrophilus*	*Aggregatibacter aphrophilus NJ8700*	182	0.481	185	PICI	2254923	2263135	8.2	
		mhae:F382_00545	*Mannheimia haemolytica*	*Mannheimia haemolytica D153*	353	0.444	160	Defective PICI?				No primase; two integrases
		mhal:N220_07655	*Mannheimia haemolytica*	*Mannheimia haemolytica USMARC_2286*	353	0.444	160	Defective PICI?				Same as *Mannheimia haemolytica D153*
		mhao:J451_00515	*Mannheimia haemolytica*	*Mannheimia haemolytica D174*	353	0.444	160	Defective PICI?				Same as *Mannheimia haemolytica D153*
		mhq:D650_15250	*Mannheimia haemolytica*	*Mannheimia haemolytica USDA-ARS-USMARC-183*	353	0.444	160	Defective PICI?				Same as *Mannheimia haemolytica D153*
		mhx:MHH_c21280	*Mannheimia haemolytica*	*Mannheimia haemolytica M42548*	353	0.444	160	Defective PICI?				Same as *Mannheimia haemolytica D153*
		mvi:X808_17960	*Mannheimia varigena*	*Mannheimia varigena USDA-ARS-USMARC-1296*	188	0.441	161	PICI	1898061	1907756	9.7	
DR93_161		pmul:DR93_161	*Pasteurella multocida*	*Pasteurella multocida ATCC 43137*	131	1.000		PmCIATCC 43137				
		pmu:PM1779	*Pasteurella multocida*	*Pasteurella multocida subsp. multocida Pm70*	136	0.607	140	PICI	1994540	2009007	14.5	Similar to PmCIPm3480
		pul:NT08PM_1617	*Pasteurella multocida*	*Pasteurella multocida subsp. multocida 3480*	73	0.808	73	PICI	1690704	1703854	13.1	
		hif:HIBPF_06730	*Haemophilus influenzae*	*Haemophilus influenzae F3031*	320	0.485	101	PICI?	617232	628294	11	Not commun structure
		hiu:HIB_05620	*Haemophilus influenzae*	*Haemophilus influenzae 10810*	309	0.500	100	PICI?	563371	577256	13.8	Not commun structure
		aacn:AANUM_0885	*Aggregatibacter actinomycetemcomitans*	*Aggregatibacter actinomycetemcomitans NUM4039*	204	0.430	107	PICI?	855426	865368	9.9	No primase
		aan:D7S_01409	*Aggregatibacter actinomycetemcomitans*	*Aggregatibacter actinomycetemcomitans D7S-1*	267	0.430	107	PICI	1321056	1330241	9.2	
		hsm:HSM_0239	*Haemophilus somnus*	*Haemophilus somnus 2336*	162	0.398	123	Defective PICI?				No integrase
		mvr:X781_11090	*Mannheimia*	*Mannheimia sp. USDA-ARS-USMARC-1261*	149	0.416	113	PICI?	1128249	1143315	15	Not commun structure; *ter*L
		aap:NT05HA_1826	*Aggregatibacter aphrophilus*	*Aggregatibacter aphrophilus NJ8700*	307	0.395	129	PICI	1811901	1821358	9.4	
		hso:HS_0528	*Haemophilus somnus*	*Haemophilus somnus 129PT*	163	0.420	88	PICI?	562065	574352	12.3	Not commun structure
DR93_160		pmul:DR93_160	*Pasteurella multocida*	*Pasteurella multocida ATCC 43137*	108	1.000		PmCIATCC 43137				
		pmp:Pmu_15580	*Pasteurella multocida*	*Pasteurella multocida 36950*	108	1.000	108	PmCIATCC 43137	1677145	1691873	14.7	
		pmu:PM1780	*Pasteurella multocida*	*Pasteurella multocida subsp. multocida Pm70*	108	0.972	108	PICI	1994540	2009007	14.5	Similar to PmCIPm3480
		pul:NT08PM_1616	*Pasteurella multocida*	*Pasteurella multocida subsp. multocida 3480*	108	0.917	108	PICI	1690704	1703854	13.1	
		eft:M395_03270	*Enterococcus faecium*	*Enterococcus faecium T110*	289	0.337	98	No insert				
		efau:EFAU085_00598	*Enterococcus faecium*	*Enterococcus faecium Aus0085*	289	0.337	98	No insert				
		efc:EFAU004_00650	*Enterococcus faecium*	*Enterococcus faecium Aus0004*	289	0.337	98	No insert				

PmCIATCC 43137	Function	Orthologs	Species	Full name	Length	Similarity	Overlap	Element	Start	End	Size	Comments
		efm:M7W_838	*Enterococcus faecium*	*Enterococcus faecium NRRL B-2354*	289	0.337	98	No insert				
		efu:HMPREF0351_10674	*Enterococcus faecium*	*Enterococcus faecium DO*	289	0.337	98	No insert				
		sbd:ATN00_10730	*Sphingobium baderi*	*Sphingobium baderi DE-13*	462	0.306	108	No insert				
		hvo:HVO_1943	*Haloferax volcanii*	*Haloferax volcanii DS2*	746	0.305	95	No insert				
DR93_159		pmul:DR93_159	*Pasteurella multocida*	*Pasteurella multocida ATCC 43137*	144	1.000		PmCIATCC 43137				
		pmp:Pmu_15570	*Pasteurella multocida*	*Pasteurella multocida 36950*	143	1.000	143	PmCIATCC 43137	1677145	1691873	14.7	
		pmu:PM1781	*Pasteurella multocida*	*Pasteurella multocida subsp. multocida Pm70*	144	0.979	144	PICI	1994540	2009007	14.5	Similar to PmCIPm3480
		pul:NT08PM_1615	*Pasteurella multocida*	*Pasteurella multocida subsp. multocida 3480*	143	0.958	144	PICI	1690704	1703854	13.1	
		aan:D7S_01406	*Aggregatibacter actinomycetemcomitans*	*Aggregatibacter actinomycetemcomitans D7S-1*	169	0.443	97	PICI	1321056	1330241	9.2	
		aacn:AANUM_0882	*Aggregatibacter actinomycetemcomitans*	*Aggregatibacter actinomycetemcomitans NUM4039*	169	0.443	97	PICI?	855426	865368	9.9	No primase
		aah:CF65_00782	*Aggregatibacter actinomycetemcomitans*	*Aggregatibacter actinomycetemcomitans HK1651*	106	0.396	91	No insert				Only integrase
		mhat:B824_4650	*Mannheimia haemolytica*	*Mannheimia haemolytica USDA-ARS-USMARC-184*	79	0.462	78	No insert				Only integrase
		aap:NT05HA_1823	*Aggregatibacter aphrophilus*	*Aggregatibacter aphrophilus NJ8700*	179	0.349	106	PICI	1811901	1821358	9.4	
		hil:HICON_15170	*Haemophilus influenzae*	*Haemophilus influenzae F3047*	227	0.317	139	PICI?	1359833	1373077	13.2	Not commun structure; similar to *H.influenzae* F3031
		hif:HIBPF_06690	*Haemophilus influenzae*	*Haemophilus influenzae F3031*	227	0.317	139	PICI?	617232	628294	11	Not commun structure
DR93_158	*pri*	pmul:DR93_158	*Pasteurella multocida*	*Pasteurella multocida ATCC 43137*	725	1.000		PmCIATCC 43137				
		pmp:Pmu_15560	*Pasteurella multocida*	*Pasteurella multocida 36950*	725	1.000	725	PmCIATCC 43137	1677145	1691873	14.7	
		pul:NT08PM_1614	*Pasteurella multocida*	*Pasteurella multocida subsp. multocida 3480*	725	0.999	725	PICI	1690704	1703854	13.1	
		pmu:PM1782	*Pasteurella multocida*	*Pasteurella multocida subsp. multocida Pm70*	725	0.992	725	PICI	1994540	2009007	14.5	Similar to PmCIPm3480
		hpas:JL26_02260	*Haemophilus parasuis*	*Haemophilus parasuis SH03*	724	0.532	726	PICI?	395669	4055177	9.8	Not commun structure
		hpaz:K756_02740	*Haemophilus parasuis*	*Haemophilus parasuis ZJ0906*	724	0.530	726	PICI?	515457	524474	9	Not commun structure; similar to *H. parasuis SH03*
		hap:HAPS_1581	*Haemophilus parasuis*	*Haemophilus parasuis SH0165*	728	0.528	726	PICI?	781463	790311	8.8	Not commun structure
		hpak:JT17_11140	*Haemophilus parasuis*	*Haemophilus parasuis KL0318*	728	0.517	726	PICI?	2198349	2207437	9.1	Not commun structure; similar to *H. parasuis SH03*
		hif:HIBPF_06680	*Haemophilus influenzae*	*Haemophilus influenzae F3031*	730	0.489	736	PICI?	617232	628294	11	Not commun structure
		hil:HICON_15160	*Haemophilus influenzae*	*Haemophilus influenzae F3047*	730	0.488	735	PICI?	1359833	1373077	13.2	Not commun structure; similar to *H.influenzae F3031*
		kqu:AVR78_10885	*Klebsiella quasipneumoniae*	*Klebsiella quasipneumoniae ATCC 700603*	704	0.429	721	PICI	5087576	5108642	21	
DR93_157	*abi*F	pmul:DR93_157	*Pasteurella multocida*	*Pasteurella multocida ATCC 43137*	316	1.000		PmCIATCC 43137				
		pmp:Pmu_15550	*Pasteurella multocida*	*Pasteurella multocida 36950*	316	1.000	316	PmCIATCC 43137	1677145	1691873	14.7	
		pul:NT08PM_1613	*Pasteurella multocida*	*Pasteurella multocida subsp. multocida 3480*	316	0.997	316	PICI	1690704	1703854	13.1	

PmCIATCC 43137	Function	Orthologs	Species	Full name	Length	Similarity	Overlap	Element	Start	End	Size	Comments
		pmu:PM1783	*Pasteurella multocida*	*Pasteurella multocida subsp. multocida Pm70*	316	0.994	316	PICI	1994540	2009007	14.5	Similar to PmCIPm3480
		mhae:F382_09970	*Mannheimia haemolytica*	*Mannheimia haemolytica D153*	315	0.521	313	No insert				
		mhal:N220_02070	*Mannheimia haemolytica*	*Mannheimia haemolytica USMARC_2286*	315	0.521	313	No insert				
		mham:J450_08895	*Mannheimia haemolytica*	*Mannheimia haemolytica D171*	315	0.521	313	No insert				
		mhao:J451_10190	*Mannheimia haemolytica*	*Mannheimia haemolytica D174*	315	0.521	313	No insert				
		mhaq:WC39_11775	*Mannheimia haemolytica*	*Mannheimia haemolytica 89010807N*	315	0.521	313	No insert				
		mhat:B824_22520	*Mannheimia haemolytica*	*Mannheimia haemolytica USDA-ARS-USMARC-184*	315	0.521	313	No insert				
		mhay:VK67_11780	*Mannheimia haemolytica*	*Mannheimia haemolytica 89010807N lktA-*	315	0.521	313	No insert				
DR93_156		pmul:DR93_156	*Pasteurella multocida*	*Pasteurella multocida ATCC 43137*	349	1.000		PmCIATCC 43137				
		pmp:Pmu_15540	*Pasteurella multocida*	*Pasteurella multocida 36950*	349	1.000	349	PmCIATCC 43137	1677145	1691873	14.7	
		eec:EcWSU1_02439	*Enterobacter cloacae*	*Enterobacter cloacae EcWSU1*	353	0.531	354	Defective phage?				
		ecf:ECH74115_3238	*Escherichia coli*	*Escherichia coli O157:H7 EC4115*	353	0.537	352	Prophage				
		ecp:ECP_1145	*Escherichia coli*	*Escherichia coli O6:K15:H31 536*	353	0.537	352	Prophage				
		ese:ECSF_1048	*Escherichia coli*	*Escherichia coli O150:H5 SE15*	357	0.537	352	Prophage				
		etw:ECSP_2982	*Escherichia coli*	*Escherichia coli O157:H7 TW14359*	353	0.537	352	Prophage				
		senj:CFSAN001992_20510	*Salmonella enterica*	*Salmonella enterica subsp. enterica serovar Javiana CFSAN001992*	353	0.511	352	Prophage				
		eok:G2583_0668	*Escherichia coli*	*Escherichia coli O55:H7 CB9615*	353	0.534	352	Prophage				
		eln:NRG857_05550	*Escherichia coli*	*Escherichia coli O83:H1 NRG 857C*	353	0.534	352	Prophage				
		vvl:VV93_v1c41070	*Vibrio vulnificus*	*Vibrio vulnificus 93U204*	351	0.490	349	No insert				
DR93_155		pmul:DR93_155	*Pasteurella multocida*	*Pasteurella multocida ATCC 43137*	124	1.000		PmCIATCC 43137				
		pmp:Pmu_15530	*Pasteurella multocida*	*Pasteurella multocida 36950*	124	1.000	124	PmCIATCC 43137	1677145	1691873	14.7	
		lrz:BJI69_06080	*Luteibacter rhizovicinus*	*Luteibacter rhizovicinus LJ96T*	124	0.565	124	No insert				
		pao:Pat9b_3438	*Pantoea*	*Pantoea sp. At-9b*	123	0.553	123	Prophage				
		aal:EP13_18185	*Alteromonas australica*	*Alteromonas australica H 17*	125	0.577	123	No insert				
		ddd:Dda3937_01763	*Dickeya dadantii*	*Dickeya dadantii 3937*	126	0.561	123	Prophage				
		vvl:VV93_v1c41080	*Vibrio vulnificus*	*Vibrio vulnificus 93U204*	132	0.565	124	No insert				
		shal:SHALO_2495	*Sulfurospirillum halorespirans*	*Sulfurospirillum halorespirans DSM 13726*	124	0.537	123	No insert				
		ecp:ECP_1144	*Escherichia coli*	*Escherichia coli O6:K15:H31 536*	125	0.537	123	Prophage				
		eln:NRG857_05545	*Escherichia coli*	*Escherichia coli O83:H1 NRG 857C*	125	0.537	123	Prophage				
		eok:G2583_0667	*Escherichia coli*	*Escherichia coli O55:H7 CB9615*	125	0.537	123	Prophage				

PmCIATCC 43137	Function	Orthologs	Species	Full name	Length	Similarity	Overlap	Element	Start	End	Size	Comments
DR93_154		pmul:DR93_154	*Pasteurella multocida*	*Pasteurella multocida ATCC 43137*	31			PmCIATCC 43137				No matches in database (DB)

Table S5. P4 orthologies.

P4	Function	Orthologs	Species	Full name	Length	Similarity	Overlap	Element	Start	End	Size	Comments
1261092	*int*	vg:1261092		*Enterobacteria phage P4*	439	1.000						
		eih:ECOK1_4788	*Escherichia coli*	*Escherichia coli IHE3034*	422	0.995	410	P4-like	4929672	4941310	11.6	
		csj:CSK29544_02175	*Cronobacter sakazakii*	*Cronobacter sakazakii ATCC 29544*	420	0.943	420	Defective P4-like				Only *int*, *psu* and *pri* fragment
		senn:SN31241_8570	*Salmonella enterica*	*Salmonella enterica subsp. enterica serovar Newport USMARC-S3124.1*	425	0.954	414	P4-like	867520	879568	12	
		ron:TE10_04950	*Raoultella ornithinolytica*	*Raoultella ornithinolytica S12*	421	0.961	410	P4-like	1067123	1079262	12.1	
		sent:TY21A_22985	*Salmonella enterica*	*Salmonella enterica subsp. enterica serovar Typhi Ty21a*	421	0.961	410	P4-like	4666556	4677971	11.4	
		stt:t4518	*Salmonella enterica*	*Salmonella enterica subsp. enterica serovar Typhi Ty2*	421	0.961	410	P4-like	4666559	4677974	11.4	
		sty:STY4821	*Salmonella enterica*	*Salmonella enterica subsp. enterica serovar Typhi CT18*	421	0.961	410	P4-like	4683670	4695086	11.4	
		eclo:ENC_43890	*Enterobacter cloacae*	*Enterobacter cloacae subsp. cloacae NCTC 9394*	418	0.940	418	P4-like	4472603	4482957	10.5	Draft genome
		seeb:SEEB0189_019950	*Salmonella enterica*	*Salmonella enterica subsp. enterica serovar Bareilly CFSAN000189*	419	0.963	410	P4-like	4089428	4101526	12	
		csi:P262_05052	*Cronobacter sakazakii*	*Cronobacter sakazakii CMCC 45402*	425	0.937	414	P4-like	3760642	3771860	11.2	
		sek:SSPA3984	*Salmonella enterica*	*Salmonella enterica subsp. enterica serovar Paratyphi A AKU1260*	419	0.961	410	P4-like	4456338	4466061	9.7	
		spt:SPA4288	*Salmonella enterica*	*Salmonella enterica subsp. enterica serovar Paratyphi A ATCC9150*	419	0.961	410	P4-like	4461107	4470831	9.7	
		sbo:SBO_4174	*Shigella boydii*	*Shigella boydii Sb227*	421	0.956	410	P4-like	4217005	4228846	11.8	
		kpz:KPNIH27_02710	*Klebsiella pneumoniae*	*Klebsiella pneumoniae subsp. pneumoniae KPNIH27*	423	0.954	410	P4-like	576483	588499	12	
		eoj:ECO26_5435	*Escherichia coli*	*Escherichia coli O26:H11 11368*	420	0.949	410	P4-like	5524027	5538050	14	
		etr:ETAE_3092	*Edwardsiella tarda*	*Edwardsiella tarda EIB202*	420	0.959	410	Defective P4-like				Only *int*, *psu* and *sid*
		kpa:KPNJ1_04920	*Klebsiella pneumoniae*	*Klebsiella pneumoniae 30660/NJST258_1*	420	0.954	410	P4-like	4695067	4706266	11.2	
		kpc:KPNIH10_02775	*Klebsiella pneumoniae*	*Klebsiella pneumoniae subsp. pneumoniae KPNIH10*	420	0.954	410	P4-like	585958	597158	11.2	
		kpg:KPNIH32_02935	*Klebsiella pneumoniae*	*Klebsiella pneumoniae subsp. pneumoniae KPNIH32*	420	0.954	410	P4-like	588185	599385	11.2	
		kph:KPNIH24_02795	*Klebsiella pneumoniae*	*Klebsiella pneumoniae subsp. pneumoniae KPNIH24*	420	0.954	410	P4-like	585958	597158	11.2	
		kpj:N559_4601	*Klebsiella pneumoniae*	*Klebsiella pneumoniae JM45*	420	0.954	410	P4-like	4681698	4692896	11.2	
		kpm:KPHS_05400	*Klebsiella pneumoniae*	*Klebsiella pneumoniae subsp. pneumoniae HS11286*	420	0.954	410	P4-like	581768	594033	12.3	
		kpne:KU54_023815	*Klebsiella pneumoniae*	*Klebsiella pneumoniae 32192*	420	0.954	410	P4-like	4703179	4714379	11.2	
		kpnu:LI86_23655	*Klebsiella pneumoniae*	*Klebsiella pneumoniae 34618*	420	0.954	410	P4-like	4682111	4693311	11.2	
		kpq:KPR0928_02785	*Klebsiella pneumoniae*	*Klebsiella pneumoniae subsp. pneumoniae KPR0928*	420	0.954	410	P4-like	585959	597159	11.2	
		kps:KPNJ2_04869	*Klebsiella pneumoniae*	*Klebsiella pneumoniae 30684/NJST258_2*	420	0.954	410	P4-like	4707358	4718557	11.2	

P4	Function	Orthologs	Species	Full name	Length	Similarity	Overlap	Element	Start	End	Size	Comments
		kpw:KPNIH30_02930	*Klebsiella pneumoniae*	*Klebsiella pneumoniae subsp. pneumoniae KPNIH30*	420	0.954	410	P4-like	585959	597159	11.2	
		kvd:KR75_10995	*Klebsiella variicola*	*Klebsiella variicola DX120E*	420	0.954	410	P4-like	4867698	4878882	11.2	
		see:SNSL254_A4836	*Salmonella enterica*	*Salmonella enterica subsp. enterica serovar Newport SL254*	421	0.950	410	P4-like	4709611	4721659	12	
		ecle:ECNIH2_03585	*Enterobacter cloacae*	*Enterobacter cloacae ECNIH2*	420	0.946	410	Defective P4-like				Disrupted *pri*
		ehm:AB284_22310	*Enterobacter hormaechei*	*Enterobacter hormaechei CAV1176*	420	0.946	410	Defective P4-like				Disrupted *pri*
		lax:APT61_19835	*Leclercia adecarboxylata*	*Leclercia adecarboxylata USDA-ARS-USMARC-60222*	417	0.946	410	Defective P4-like				Only *int* and *psu*
		ecoi:ECOPMV1_04739	*Escherichia coli*	*Escherichia coli PMV-1*	417	0.937	410	P4-like	4850307	4861972	11.6	
		serf:L085_01585	*Serratia*	*Serratia sp. FS14*	425	0.868	409	Defective P4-like				Only *int*
		ebi:EbC_39600	*Erwinia billingiae*	*Erwinia billingiae Eb661*	417	0.856	410	P4-like	4406158	4418027	11.9	
1261084	*cII*	vg:1261084		*Enterobacteria phage P4*	264	1.000						
		eih:ECOK1_4789	*Escherichia coli*	*Escherichia coli IHE3034*	264	1.000	264	P4-like	4929672	4941310	11.6	
		raq:Rahaq2_3977	*Rahnella aquatilis*	*Rahnella aquatilis CIP 78.65 = ATCC 33071*	266	0.532	265	Defective P4-like				*ogr*, *beta* and *gop*
		bug:BC1001_0005	*Burkholderia*	*Burkholderia sp. CCGE1001*	273	0.498	259	Defective P4-like				Only *cII*, *gop* and *beta*
		rsl:RPSI07_0197	*Ralstonia solanacearum*	*Ralstonia solanacearum PSI07*	273	0.372	269	Defective P4-like				Only *cII* and *beta*
		van:VAA_00672	*Vibrio anguillarum*	*Vibrio anguillarum 775*	274	0.343	274	No insert				
		vpk:M636_19810	*Vibrio parahaemolyticus*	*Vibrio parahaemolyticus O1:K33 CDC_K4557*	274	0.343	274	No insert				
		lag:N175_13100	*Vibrio anguillarum*	*Vibrio anguillarum M3*	270	0.344	270	Defective P4-like				
		vau:VANGNB10_cI0418	*Vibrio anguillarum*	*Vibrio anguillarum NB10*	270	0.344	270	No insert				
		del:DelCs14_4395	*Delftia*	*Delftia sp. Cs1-4*	218	0.363	190	Defective P4-like				Only *cII* and *beta*
		psy:PCNPT3_00440	*Psychromonas*	*Psychromonas sp. CNPT3*	237	0.341	214	No insert				
1261083	*beta*	vg:1261083		*Enterobacteria phage P4*	355	1.000						
		eih:ECOK1_4790	*Escherichia coli*	*Escherichia coli IHE3034*	355	1.000	355	P4-like	4929672	4941310	11.6	
		raq:Rahaq2_3978	*Rahnella aquatilis*	*Rahnella aquatilis CIP 78.65 = ATCC 33071*	353	0.701	351	Defective P4-like				*ogr*, *beta* and *gop*
		bug:BC1001_0007	*Burkholderia*	*Burkholderia sp. CCGE1001*	353	0.588	352	Defective P4-like				Only *cII*, *gop* and *beta*
		mpt:Mpe_A1938	*Methylibium petroleiphilum*	*Methylibium petroleiphilum PM1*	359	0.438	356	No insert				
		del:DelCs14_4396	*Delftia*	*Delftia sp. Cs1-4*	360	0.429	361	Defective P4-like				Only *cII* and *beta*
		rsl:RPSI07_0196	*Ralstonia solanacearum*	*Ralstonia solanacearum PSI07*	354	0.415	352	Defective P4-like				Only *cII* and *beta*
		hpas:JL26_02970	*Haemophilus parasuis*	*Haemophilus parasuis SH03*	369	0.378	360	P4-like? Phage?				Phage proteins: *lexA*…
		hpaz:K756_05755	*Haemophilus parasuis*	*Haemophilus parasuis ZJ0906*	369	0.378	360	P4-like? Phage?				Phage proteins: *lexA*…

P4	Function	Orthologs	Species	Full name	Length	Similarity	Overlap	Element	Start	End	Size	Comments
		gan:UMN179_02358	*Gallibacterium anatis*	*Gallibacterium anatis UMN179*	368	0.350	374	Phage				
		lag:N175_13095	*Vibrio anguillarum*	*Vibrio anguillarum M3*	376	0.306	369	Defective P4-like				
1261097	*gop*	vg:1261097		*Enterobacteria phage P4*	133	1.000						
		raq:Rahaq2_3979	*Rahnella aquatilis*	*Rahnella aquatilis CIP 78.65 = ATCC 33071*	137	0.481	133	Defective P4-like				*ogr, beta* and *gop*
		bug:BC1001_0008	*Burkholderia*	*Burkholderia sp. CCGE1001*	141	0.350	137	Defective P4-like				Only *cII, gop* and *beta*
		cbr:CBG19597	*Caenorhabditis briggsae*	*Caenorhabditis briggsae AF16*	513	0.307	88					RefSeq (Assembly: GCF_000004555.1)
		maj:MAA_08300	*Metarhizium robertsii*	*Metarhizium robertsii ARSEF 23*	1753	0.329	82					RefSeq (Assembly: GCF_000187425.2)
		cac:CA_C0250	*Clostridium acetobutylicum*	*Clostridium acetobutylicum ATCC 824*	688	0.353	68	No insert				
		cae:SMB_G0255	*Clostridium acetobutylicum*	*Clostridium acetobutylicum DSM 1731*	688	0.353	68	No insert				
		cay:CEA_G0256	*Clostridium acetobutylicum*	*Clostridium acetobutylicum EA 2018*	688	0.353	68	No insert				
		srd:SD10_27760	*Spirosoma radiotolerans*	*Spirosoma radiotolerans DG5A*	494	0.306	98	No insert				
		nsl:BOX37_10940	*Nocardia soli*	*Nocardia soli Y48*	330	0.339	56	No insert				
		sli:Slin_5408	*Spirosoma linguale*	*Spirosoma linguale DSM 74*	494	0.060	98	No insert				
1261094	*psu*	vg:1261094		*Enterobacteria phage P4*	190	1.000						
		ecoo:ECRM13514_3795	*Escherichia coli*	*Escherichia coli O145:H28 RM13514*	190	0.995	190	P4-like	3690842	3702177	11.3	
		eoj:ECO26_4938	*Escherichia coli*	*Escherichia coli O26:H11 11368*	190	0.995	190	P4-like	4952573	4966595	14	
		sent:TY21A_13410	*Salmonella enterica*	*Salmonella enterica subsp. enterica serovar Typhi Ty21a*	190	0.995	190	P4-like	2734973	2745406	10.4	
		stt:t2648	*Salmonella enterica*	*Salmonella enterica subsp. enterica serovar Typhi Ty2*	190	0.995	190	P4-like	2734973	2745406	10.4	Same as *S. enterica Ty21a*
		ecl:EcolC_1061	*Escherichia coli*	*Escherichia coli ATCC 8739*	190	0.989	190	P4-like	45053	55477	10.4	
		eih:ECOK1_4791	*Escherichia coli*	*Escherichia coli IHE3034*	190	0.989	190	P4-like	4929672	4941310	11.6	
		eal:EAKF1_ch3364	*Escherichia albertii*	*Escherichia albertii KF1*	190	0.984	190	Defective P4-like				Several *int*
		sbo:SBO_4171	*Shigella boydii*	*Shigella boydii Sb227*	190	0.984	190	P4-like	4217005	4228846	11.8	
		seeh:SEEH1578_04860	*Salmonella enterica*	*Salmonella enterica subsp. enterica serovar Heidelberg 41578*	190	0.984	190	P4-like	885261	896545	11.3	
		efe:EFER_0448	*Escherichia fergusonii*	*Escherichia fergusonii ATCC 35469*	190	0.984	190	P4-like	448071	459263	11.2	
		ecoi:ECOPMV1_04741	*Escherichia coli*	*Escherichia coli PMV-1*	190	0.984	190	P4-like	4850307	4861972	11.6	
		ecoh:ECRM13516_4455	*Escherichia coli*	*Escherichia coli O145:H28 RM13516*	190	0.958	190	P4-like	4410536	4422590	12	
		ecm:EcSMS35_4004	*Escherichia coli*	*Escherichia coli SMS-3-5*	190	0.953	190	P4-like	4081414	4092685	11.3	
		elo:EC042_3995	*Escherichia coli*	*Escherichia coli O44:H18 042*	190	0.942	190	P4-like	4236680	4247745	11	
		cmw:AFK63_19225	*Cronobacter muytjensii*	*Cronobacter muytjensii ATCC 51329*	188	0.651	186	P4-like	4183320	4195597	12.3	

P4	Function	Orthologs	Species	Full name	Length	Similarity	Overlap	Element	Start	End	Size	Comments
		enl:A3UG_00580	*Enterobacter cloacae*	*Enterobacter cloacae subsp. dissolvens SDM*	187	0.660	188	P4-like	104977	116426	11.5	
		etd:ETAF_0744	*Edwardsiella tarda*	*Edwardsiella tarda FL6-60*	188	0.638	188	P4-like	864265	875965	11.7	
1261088	*delta*	vg:1261088		*Enterobacteria phage P4*	166	1.000						
		eih:ECOK1_4792	*Escherichia coli*	*Escherichia coli IHE3034*	166	0.982	166	P4-like	4929672	4941310	11.6	
		efe:EFER_0447	*Escherichia fergusonii*	*Escherichia fergusonii ATCC 35469*	166	0.982	166	P4-like	448071	459263	11.2	
		ecoo:ECRM13514_3794	*Escherichia coli*	*Escherichia coli O145:H28 RM13514*	166	0.976	166	P4-like	3690842	3702177	11.3	
		ecl:EcolC_1060	*Escherichia coli*	*Escherichia coli ATCC 8739*	166	0.976	166	P4-like	1146254	1156920	10.7	
		eoj:ECO26_4937	*Escherichia coli*	*Escherichia coli O26:H11 11368*	166	0.976	166	P4-like	4952573	4966595	14	
		ecoi:ECOPMV1_04742	*Escherichia coli*	*Escherichia coli PMV-1*	166	0.970	166	P4-like	4850307	4861972	11.6	
		eal:EAKF1_ch3363	*Escherichia albertii*	*Escherichia albertii KF1*	55	0.982	55	Defective P4-like				Several *int*
		seeh:SEEH1578_04865	*Salmonella enterica*	*Salmonella enterica subsp. enterica serovar Heidelberg 41578*	55	0.945	55	P4-like	885261	896545	11.3	
		sent:TY21A_13415	*Salmonella enterica*	*Salmonella enterica subsp. enterica serovar Typhi Ty21a*	55	0.945	55	P4-like	2734973	2745406	10.4	
		stt:t2649	*Salmonella enterica*	*Salmonella enterica subsp. enterica serovar Typhi Ty2*	55	0.945	55	P4-like	2734973	2745406	10.4	Same as *S. enterica Ty21a*
		serf:L085_00060	*Serratia*	*Serratia sp. FS14*	55	0.545	44	Defective P4-like?				Only *delta* and a lysozime
		sers:SERRSCBI_03910	*Serratia*	*Serratia sp. SCBI*	98	0.545	44	Defective P4-like?				Only *delta* and a lysozime
		smac:SMDB11_0175	*Serratia marcescens*	*Serratia marcescens subsp. marcescens Db11*	75	0.545	44	Defective P4-like?				Only *delta* and a lysozime
1261087	*sid*	vg:1261087		*Enterobacteria phage P4*	244	1.000						
		ecoh:ECRM13516_4457	*Escherichia coli*	*Escherichia coli O145:H28 RM13516*	244	0.992	244	P4-like	4410536	4422590	12	
		elo:EC042_3996	*Escherichia coli*	*Escherichia coli O44:H18 042*	244	0.996	244	P4-like	4236680	4247745	11	
		sbo:SBO_4170	*Shigella boydii*	*Shigella boydii Sb227*	244	0.996	244	P4-like	4217005	4228846	11.8	
		ecl:EcolC_0049	*Escherichia coli*	*Escherichia coli ATCC 8739*	244	0.988	244	P4-like	45053	55477	10.4	
		ecoi:ECOPMV1_04743	*Escherichia coli*	*Escherichia coli PMV-1*	244	0.992	244	P4-like	4850307	4861972	11.6	
		efe:EFER_0446	*Escherichia fergusonii*	*Escherichia fergusonii ATCC 35469*	266	0.992	244	P4-like	448071	459263	11.2	
		ecoo:ECRM13514_3793	*Escherichia coli*	*Escherichia coli O145:H28 RM13514*	244	0.988	244	P4-like	3690842	3702177	11.3	
		eih:ECOK1_4793	*Escherichia coli*	*Escherichia coli IHE3034*	244	0.980	244	P4-like	4929672	4941310	11.6	
		ecm:EcSMS35_4005	*Escherichia coli*	*Escherichia coli SMS-3-5*	244	0.980	244	P4-like	4081414	4092685	11.3	
		eoj:ECO26_4936	*Escherichia coli*	*Escherichia coli O26:H11 11368*	244	0.984	244	P4-like	4952573	4966595	14	

P4	Function	Orthologs	Species	Full name	Length	Similarity	Overlap	Element	Start	End	Size	Comments
		sent:TY21A_13420	*Salmonella enterica*	*Salmonella enterica subsp. enterica serovar Typhi Ty21a*	244	0.980	244	P4-like	2734973	2745406	10.4	
		stt:t2650	*Salmonella enterica*	*Salmonella enterica subsp. enterica serovar Typhi Ty2*	244	0.980	244	P4-like	2734973	2745406	10.4	Same as *S. enterica Ty21a*
		seeh:SEEH1578_04870	*Salmonella enterica*	*Salmonella enterica subsp. enterica serovar Heidelberg 41578*	244	0.980	244	P4-like	885261	896545	11.3	
		seeb:SEEB0189_019925	*Salmonella enterica*	*Salmonella enterica subsp. enterica serovar Bareilly CFSAN000189*	244	0.657	239	P4-like	4089428	4101526	12	
		senj:CFSAN001992_19715	*Salmonella enterica*	*Salmonella enterica subsp. enterica serovar Javiana CFSAN001992*	245	0.653	239	P4-like	4078550	4089203	10.6	
1261090	*alp*A	vg:1261090		*Enterobacteria phage P4*	88	1.000						
		ecl:EcolC_1058	*Escherichia coli*	*Escherichia coli ATCC 8739*	88	0.989	88	P4-like	1146254	1156920	10.7	
		ecm:EcSMS35_4006	*Escherichia coli*	*Escherichia coli SMS-3-5*	88	0.989	88	P4-like	4081414	4092685	11.3	
		ecoh:ECRM13516_4459	*Escherichia coli*	*Escherichia coli O145:H28 RM13516*	88	0.989	88	P4-like	4410536	4422590	12	
		ecoi:ECOPMV1_04744	*Escherichia coli*	*Escherichia coli PMV-1*	88	0.989	88	P4-like	4850307	4861972	11.6	
		ecoo:ECRM13514_3792	*Escherichia coli*	*Escherichia coli O145:H28 RM13514*	88	0.989	88	P4-like	3690842	3702177	11.3	
		efe:EFER_0445	*Escherichia fergusonii*	*Escherichia fergusonii ATCC 35469*	88	0.989	88	P4-like	448071	459263	11.2	
		eih:ECOK1_4794	*Escherichia coli*	*Escherichia coli IHE3034*	88	0.989	88	P4-like	4929672	4941310	11.6	
		elo:EC042_3997	*Escherichia coli*	*Escherichia coli O44:H18 042*	88	0.989	88	P4-like	4236680	4247745	11	
		eoj:ECO26_4934	*Escherichia coli*	*Escherichia coli O26:H11 11368*	88	0.989	88	P4-like	4952573	4966595	14	
		sbo:SBO_4169	*Shigella boydii*	*Shigella boydii Sb227*	88	0.989	88	P4-like	4217005	4228846	11.8	
		seeh:SEEH1578_04875	*Salmonella enterica*	*Salmonella enterica subsp. enterica serovar Heidelberg 41578*	88	0.989	88	P4-like	885261	896545	11.3	
		sent:TY21A_13425	*Salmonella enterica*	*Salmonella enterica subsp. enterica serovar Typhi Ty21a*	88	0.977	88	P4-like	2734973	2745406	10.4	
		stt:t2651	*Salmonella enterica*	*Salmonella enterica subsp. enterica serovar Typhi Ty2*	72	0.972	72	P4-like	2734973	2745406	10.4	Same as *S. enterica Ty21a*
		csi:P262_05045	*Cronobacter sakazakii*	*Cronobacter sakazakii CMCC 45402*	88	0.750	88	P4-like	3760642	3771860	11.2	
		kpv:KPNIH29_25720	*Klebsiella pneumoniae*	*Klebsiella pneumoniae subsp. pneumoniae KPNIH29*	88	0.716	88	P4-like	5163114	5174288	11.2	
1261091	*cI*	vg:1261091		*Enterobacteria phage P4*	138	1.000						
		eoj:ECO26_4933	*Escherichia coli*	*Escherichia coli O26:H11 11368*	199	0.993	138	P4-like	4952573	4966595	14	
		sbo:SBO_4168	*Shigella boydii*	*Shigella boydii Sb227*	199	0.993	138	P4-like	4217005	4228846	11.8	
		eih:ECOK1_4795	*Escherichia coli*	*Escherichia coli IHE3034*	199	0.971	138	P4-like	4929672	4941310	11.6	
		ecoh:ECRM13516_4460	*Escherichia coli*	*Escherichia coli O145:H28 RM13516*	199	0.935	138	P4-like	4410536	4422590	12	
		efe:EFER_0444	*Escherichia fergusonii*	*Escherichia fergusonii ATCC 35469*	137	0.927	137	P4-like	448071	459263	11.2	

P4	Function	Orthologs	Species	Full name	Length	Similarity	Overlap	Element	Start	End	Size	Comments
		ecoo:ECRM13514_4676	*Escherichia coli*	*Escherichia coli O145:H28 RM13514*	199	0.906	138	P4-like	4558846	4569909	11	
		ecl:EcolC_0047	*Escherichia coli*	*Escherichia coli ATCC 8739*	196	0.848	138	P4-like	45053	55477	10.4	
		seeh:SEEH1578_04880	*Salmonella enterica*	*Salmonella enterica subsp. enterica serovar Heidelberg 41578*	196	0.848	138	P4-like	885261	896545	11.3	
		sent:TY21A_13430	*Salmonella enterica*	*Salmonella enterica subsp. enterica serovar Typhi Ty21a*	196	0.848	138	P4-like	2734973	2745406	10.4	
		ecm:EcSMS35_4007	*Escherichia coli*	*Escherichia coli SMS-3-5*	134	0.854	137	P4-like	4081414	4092685	11.3	
		stt:t2652	*Salmonella enterica*	*Salmonella enterica subsp. enterica serovar Typhi Ty2*	134	0.854	137	P4-like	2734973	2745406	10.4	Same as *S. enterica Ty21a*
		elo:EC042_3998	*Escherichia coli*	*Escherichia coli O44:H18 042*	134	0.825	137	P4-like	4236680	4247745	11	
		ses:SARI_03878	*Salmonella enterica*	*Salmonella enterica subsp. arizonae serovar 62:z4,z23:- RSK2980*	200	0.717	138	P4-like	3804516	3815458	10.9	
		kok:KONIH1_28730	*Klebsiella oxytoca*	*Klebsiella oxytoca KONIH1*	183	0.704	115	P4-like	5994027	6004738	10.7	
1261085	epsilon	vg:1261085		*Enterobacteria phage P4*	95	1.000						
		ecm:EcSMS35_4008	*Escherichia coli*	*Escherichia coli SMS-3-5*	95	0.989	95	P4-like	4081414	4092685	11.3	
		ecoh:ECRM13516_4461	*Escherichia coli*	*Escherichia coli O145:H28 RM13516*	95	0.989	95	P4-like	4410536	4422590	12	
		ecoi:ECOPMV1_04748	*Escherichia coli*	*Escherichia coli PMV-1*	95	0.989	95	P4-like	4850307	4861972	11.6	
		ecoo:ECRM13514_3790	*Escherichia coli*	*Escherichia coli O145:H28 RM13514*	95	0.989	95	P4-like	3690842	3702177	11.3	
		efe:EFER_0443	*Escherichia fergusonii*	*Escherichia fergusonii ATCC 35469*	95	0.989	95	P4-like	448071	459263	11.2	
		eih:ECOK1_4796	*Escherichia coli*	*Escherichia coli IHE3034*	95	0.989	95	P4-like	4929672	4941310	11.6	
		elo:EC042_3999	*Escherichia coli*	*Escherichia coli O44:H18 042*	95	0.989	95	P4-like	4236680	4247745	11	
		eoj:ECO26_4932	*Escherichia coli*	*Escherichia coli O26:H11 11368*	95	0.989	95	P4-like	4952573	4966595	14	
		seeh:SEEH1578_04885	*Salmonella enterica*	*Salmonella enterica subsp. enterica serovar Heidelberg 41578*	95	0.989	95	P4-like	885261	896545	11.3	
		sent:TY21A_13435	*Salmonella enterica*	*Salmonella enterica subsp. enterica serovar Typhi Ty21a*	95	0.989	95	P4-like	2734973	2745406	10.4	
		stt:t2653	*Salmonella enterica*	*Salmonella enterica subsp. enterica serovar Typhi Ty2*	95	0.989	95	P4-like	2734973	2745406	10.4	Same as *S. enterica Ty21a*
		ecl:EcolC_0046	*Escherichia coli*	*Escherichia coli ATCC 8739*	95	0.979	95	P4-like	45053	55477	10.4	
		ecle:ECNIH2_03625	*Enterobacter cloacae*	*Enterobacter cloacae ECNIH2*	99	0.687	99	Defective P4-like				Disrupted *pri*
		ehm:AB284_22265	*Enterobacter hormaechei*	*Enterobacter hormaechei CAV1176*	99	0.687	99	Defective P4-like				Disrupted *pri*
1261086	ORF151	vg:1261086		*Enterobacteria phage P4*	151	1.000						
		seeh:SEEH1578_04890	*Salmonella enterica*	*Salmonella enterica subsp. enterica serovar Heidelberg 41578*	151	1.000	151	P4-like	885261	896545	11.3	
		sent:TY21A_13440	*Salmonella enterica*	*Salmonella enterica subsp. enterica serovar Typhi Ty21a*	151	1.000	151	P4-like	2734973	2745406	10.4	

P4	Function	Orthologs	Species	Full name	Length	Similarity	Overlap	Element	Start	End	Size	Comments
		stt:t2654	*Salmonella enterica*	*Salmonella enterica subsp. enterica serovar Typhi Ty2*	151	1.000	151	P4-like	2734973	2745406	10.4	Same as *S. enterica Ty21a*
		ecl:EcolC_0045	*Escherichia coli*	*Escherichia coli ATCC 8739*	151	0.993	151	P4-like	45053	55477	10.4	
		ecoo:ECRM13514_3789	*Escherichia coli*	*Escherichia coli O145:H28 RM13514*	151	0.993	151	P4-like	3690842	3702177	11.3	
		sbo:SBO_4167	*Shigella boydii*	*Shigella boydii Sb227*	151	0.993	151	P4-like	4217005	4228846	11.8	
		eih:ECOK1_4797	*Escherichia coli*	*Escherichia coli IHE3034*	151	0.987	151	P4-like	4929672	4941310	11.6	
		ecoi:ECOPMV1_04749	*Escherichia coli*	*Escherichia coli PMV-1*	151	0.987	151	P4-like	4850307	4861972	11.6	
		efe:EFER_0442	*Escherichia fergusonii*	*Escherichia fergusonii ATCC 35469*	151	0.987	151	P4-like	448071	459263	11.2	
		ecm:EcSMS35_4009	*Escherichia coli*	*Escherichia coli SMS-3-5*	151	0.987	151	P4-like	4081414	4092685	11.3	
		ecoh:ECRM13516_4462	*Escherichia coli*	*Escherichia coli O145:H28 RM13516*	151	0.980	151	P4-like	4410536	4422590	12	
		eoj:ECO26_4931	*Escherichia coli*	*Escherichia coli O26:H11 11368*	151	0.974	151	P4-like	4952573	4966595	14	
		elo:EC042_4000	*Escherichia coli*	*Escherichia coli O44:H18 042*	151	0.974	151	P4-like	4236680	4247745	11	
		ses:SARI_03876	*Salmonella enterica*	*Salmonella enterica subsp. arizonae serovar 62:z4,z23:- RSK2980*	149	0.702	151	P4-like	3804516	3815458	10.9	
		ecle:ECNIH2_03630	*Enterobacter cloacae*	*Enterobacter cloacae ECNIH2*	149	0.675	151	Defective P4-like				Disrupted *pri*
		ehm:AB284_22260	*Enterobacter hormaechei*	*Enterobacter hormaechei CAV1176*	149	0.675	151	Defective P4-like				Disrupted *pri*
		dat:HRM2_26500	*Desulfobacterium autotrophicum*	*Desulfobacterium autotrophicum HRM2*	1206	0.355	107	No insert				
1261093	ORF106	vg:1261093		*Enterobacteria phage P4*	106	1.000						
		ecl:EcolC_0044	*Escherichia coli*	*Escherichia coli ATCC 8739*	106	1.000	106	P4-like	45053	55477	10.4	
		ecm:EcSMS35_4010	*Escherichia coli*	*Escherichia coli SMS-3-5*	106	1.000	106	P4-like	4081414	4092685	11.3	
		ecoh:ECRM13516_4463	*Escherichia coli*	*Escherichia coli O145:H28 RM13516*	106	1.000	106	P4-like	4410536	4422590	12	
		ecoi:ECOPMV1_04750	*Escherichia coli*	*Escherichia coli PMV-1*	106	1.000	106	P4-like	4850307	4861972	11.6	
		ecoo:ECRM13514_3788	*Escherichia coli*	*Escherichia coli O145:H28 RM13514*	106	1.000	106	P4-like	3690842	3702177	11.3	
		eih:ECOK1_4798	*Escherichia coli*	*Escherichia coli IHE3034*	106	1.000	106	P4-like	4929672	4941310	11.6	
		elo:EC042_4001	*Escherichia coli*	*Escherichia coli O44:H18 042*	106	1.000	106	P4-like	4236680	4247745	11	
		eoj:ECO26_4927	*Escherichia coli*	*Escherichia coli O26:H11 11368*	106	1.000	106	P4-like	4952573	4966595	14	
		sbo:SBO_4166	*Shigella boydii*	*Shigella boydii Sb227*	106	1.000	106	P4-like	4217005	4228846	11.8	
		seeh:SEEH1578_04895	*Salmonella enterica*	*Salmonella enterica subsp. enterica serovar Heidelberg 41578*	106	1.000	106	P4-like	885261	896545	11.3	
		sent:TY21A_13445	*Salmonella enterica*	*Salmonella enterica subsp. enterica serovar Typhi Ty21a*	106	1.000	106	P4-like	2734973	2745406	10.4	
		stt:t2655	*Salmonella enterica*	*Salmonella enterica subsp. enterica serovar Typhi Ty2*	106	1.000	106	P4-like	2734973	2745406	10.4	Same as *S. enterica Ty21a*

P4	Function	Orthologs	Species	Full name	Length	Similarity	Overlap	Element	Start	End	Size	Comments
		efe:EFER_0441	*Escherichia fergusonii*	*Escherichia fergusonii ATCC 35469*	106	0.991	106	P4-like	448071	459263	11.2	
		senj:CFSAN001992_19695	*Salmonella enterica*	*Salmonella enterica subsp. enterica serovar Javiana CFSAN001992*	106	0.925	106	P4-like	4078550	4089203	10.6	
		ses:SARI_03874	*Salmonella enterica*	*Salmonella enterica subsp. arizonae serovar 62:z4,z23:- RSK2980*	106	0.925	106	P4-like	3804516	3815458	10.9	
		seb:STM474_2872	*Salmonella enterica*	*Salmonella enterica subsp. enterica serovar Typhimurium ST4/74*	106	0.915	106	P4-like	2890042	2900481	10.4	
		sef:UMN798_2969	*Salmonella enterica*	*Salmonella enterica subsp. enterica serovar Typhimurium 798*	106	0.915	106	P4-like	2888784	2899223	10.4	Same as *S. typhimurium ST4/74*
		sey:SL1344_2721	*Salmonella enterica*	*Salmonella enterica subsp. enterica serovar Typhimurium SL1344*	106	0.915	106	P4-like	2890042	2900481	10.4	Same as *S. typhimurium ST4/74*
		sty:STY4831	*Salmonella enterica*	*Salmonella enterica subsp. enterica serovar Typhi CT18*	106	0.915	106	P4-like	4683670	4695086	11.4	
		ron:TE10_01825	*Raoultella ornithinolytica*	*Raoultella ornithinolytica S12*	106	0.915	106	P4-like	377086	387244	10.2	
		seeb:SEEB0189_019905	*Salmonella enterica*	*Salmonella enterica subsp. enterica serovar Bareilly CFSAN000189*	106	0.915	106	P4-like	4089428	4101526	12	
		sek:SSPA3992	*Salmonella enterica*	*Salmonella enterica subsp. enterica serovar Paratyphi A AKU12601*	106	0.906	106	P4-like	4456338	4466061	9.7	
		senb:BN855_28010	*Salmonella enterica*	*Salmonella enterica subsp. enterica serovar Bovismorbificans 3114*	106	0.915	106	P4-like	2783062	2792517	9.5	
		spq:SPAB_03425	*Salmonella enterica*	*Salmonella enterica subsp. enterica serovar Paratyphi B*	106	0.915	106	Defective P4-like				No *int*
		spt:SPA4298	*Salmonella enterica*	*Salmonella enterica subsp. enterica serovar Paratyphi A ATCC9150*	106	0.906	106	Defective P4-like				No *int*
		etd:ETAF_0749	*Edwardsiella tarda*	*Edwardsiella tarda FL6-60*	106	0.906	106	P4-like	864265	875965	11.7	
		ecle:ECNIH2_03640	*Enterobacter cloacae*	*Enterobacter cloacae ECNIH2*	106	0.887	106	Defective P4-like				Disrupted *pri*
		ehm:AB284_22250	*Enterobacter cloacae*	*Enterobacter hormaechei CAV1176*	106	0.887	106	Defective P4-like				Disrupted *pri*
1261095	*pri/alpha*	vg:1261095		*Enterobacteria phage P4*	777	1.000						
		efe:EFER_0440	*Escherichia fergusonii*	*Escherichia fergusonii ATCC 35469*	777	0.994	777	P4-like	448071	459263	11.2	
		ecm:EcSMS35_4012	*Escherichia coli*	*Escherichia coli SMS-3-5*	777	0.994	777	P4-like	4081414	4092685	11.3	
		elo:EC042_4002	*Escherichia coli*	*Escherichia coli O44:H18 042*	777	0.995	776	P4-like	4236680	4247745	11	
		ecl:EcolC_0043	*Escherichia coli*	*Escherichia coli ATCC 8739*	777	0.992	777	P4-like	45053	55477	10.4	
		eoj:ECO26_4926	*Escherichia coli*	*Escherichia coli O26:H11 11368*	777	0.992	776	P4-like	4952573	4966595	14	
		ecoo:ECRM13514_3787	*Escherichia coli*	*Escherichia coli O145:H28 RM13514*	777	0.992	777	P4-like	3690842	3702177	11.3	
		sent:TY21A_13450	*Salmonella enterica*	*Salmonella enterica subsp. enterica serovar Typhi Ty21a*	777	0.991	776	P4-like	2734973	2745406	10.4	
		stt:t2656	*Salmonella enterica*	*Salmonella enterica subsp. enterica serovar Typhi Ty2*	777	0.991	776	P4-like	2734973	2745406	10.4	
		eih:ECOK1_4799	*Escherichia coli*	*Escherichia coli IHE3034*	777	0.991	776	P4-like	4929672	4941310	11.6	
		ecoi:ECOPMV1_04751	*Escherichia coli*	*Escherichia coli PMV-1*	777	0.991	776	P4-like	4850307	4861972	11.6	
		seeh:SEEH1578_04900	*Salmonella enterica*	*Salmonella enterica subsp. enterica serovar Heidelberg 41578*	777	0.990	776	P4-like	885261	896545	11.3	
		sbo:SBO_4165	*Shigella boydii*	*Shigella boydii Sb227*	777	0.988	776	P4-like	4217005	4228846	11.8	

P4	Function	Orthologs	Species	Full name	Length	Similarity	Overlap	Element	Start	End	Size	Comments
		ecoh:ECRM13516_4464	*Escherichia coli*	*Escherichia coli O145:H28 RM13516*	777	0.988	777	P4-like	4410536	4422590	12	
		sty:STY4832	*Salmonella enterica*	*Salmonella enterica subsp. enterica serovar Typhi CT18*	777	0.862	774	P4-like	4683670	4695086	11.4	
		ecln:ECNIH4_05540	*Enterobacter cloacae*	*Enterobacter cloacae ECNIH4*	777	0.846	774	P4-like	1150352	1161464	11.1	
		enl:A3UG_00545	*Enterobacter cloacae*	*Enterobacter cloacae subsp. dissolvens SDM*	777	0.845	774	P4-like	104980	116427	11.5	
		ebt:EBL_c09330	*Shimwellia blattae*	*Shimwellia blattae DSM 4481 = NBRC 105725*	777	0.848	774	P4-like	944228	954134	9.9	
		etd:ETAF_0750	*Edwardsiella tarda*	*Edwardsiella tarda FL6-60*	777	0.850	774	P4-like	864265	875965	11.7	
1261089		vg:1261089		*Enterobacteria phage P4*	111	1.000						
		ecoi:ECOPMV1_04752	*Escherichia coli*	*Escherichia coli PMV-1*	111	0.928	111	P4-like	4850307	4861972	11.6	
		eih:ECOK1_4800	*Escherichia coli*	*Escherichia coli IHE3034*	78	0.974	77	P4-like	4929672	4941310	11.6	
		efe:EFER_0439	*Escherichia fergusonii*	*Escherichia fergusonii ATCC 35469*	72	0.915	47	P4-like	448071	459263	11.2	
		ecoo:ECRM13514_4682	*Escherichia coli*	*Escherichia coli O145:H28 RM13514*	48	0.915	47	P4-like	4558846	4569909	11	
		eal:EAKF1_ch3360c	*Escherichia albertii*	*Escherichia albertii KF1*	38	0.882	34	Defective P4-like				Several *int*
		stt:t4530	*Salmonella enterica*	*Salmonella enterica subsp. enterica serovar Typhi Ty2*	46	0.605	38	P4-like	4666559	4677974	11.4	
		ete:ETEE_0923	*Edwardsiella anguillarum*	*Edwardsiella anguillarum ET080813*	70	0.526	38	P4-like	864925	874993	10	
		enc:ECL_00700	*Enterobacter cloacae*	*Enterobacter cloacae subsp. cloacae ATCC 13047*	47	0.526	38	Defective P4-like				No *int*
		pmo:Pmob_0545	*Petrotoga mobilis*	*Petrotoga mobilis SJ95*	450	0.305	95	No insert				
		pri:PRIO_5948	*Paenibacillus riograndensis*	*Paenibacillus riograndensis SBR5*	662	0.333	57	No insert				

Table S6. Oligonucleotides used in this study.

Strain	Oligonucleotides	Sequence (5'-3')
**CFT073 *lacZ*::tetA**	EcoCFT073-lacZ-1m	AATGGATTTCCTTACGCGAAATACGGGCAGACATAGCCTGCCCGGTTATTATTATTTTTGCGCTGTTAATCACTTTACTT
	EcoCFT073-lacZ-2c	GGCTCGTATGTTGTGTGAAATTGTGAGCGGATAACAATTTCACACAGGATACAGCTATGACTGGTTATCAAGAGGGTCATTA
**CFT073 c1501::tetA**	EcoCFT073-tet-1m	TACGATGGCAGAAACCAGGGCGATGACGGCGACATCGATCGCATACTGAAAACCATCTGACGCTGTTAATCACTTTACTT
	EcoCFT073-tet-2c	CCCCGTAACGGGTCCATATGCCGGAAAGGACCCATAAAAAAGCCGGATTTCTCCGGCCTTGTCGGTTATCAAGAGGGTCATTA
**CFT073 *Δint***	EcoCFT073-int-2m	CAACGATCGGTGTAGTTAATGGTGTAGTTAATTGTGATGACCGGAATCAAAATTATGAGCGTGTAGGCTGGAGCTGCTTCG
	EcoCFT073-int-3c	TTGTGTCTCGTTGTTTCAGAGTTGTCCGCATCCTGAAAAATCGCAAAAAAAATTATATTTCCATATGAATATCCTCCTTA
**CFT073 *ΔalpA***	EcoCFT073-rep-6m	ACCCAACCCAATAATCCACCCAATGACTCTTTAAGAAACAACAAAGGGGGAATTGTGTTAGTGTAGGCTGGAGCTGCTTCG
	EcoCFT073-rep-8c	GTTAGATCTCTGTATTAGTGGATGGGTGGCGGCTGTGTGCCGCCAGCCTGATTAGTGAACCATATGAATATCCTCCTTA
**CFT073 *Δpri***	EcoCFT073-pri-3m	GGCACGGGCAAACATCCAGAAGCTGAAAACCATGATTAACGGATTCAGGGGTAAAAAATGGTGTAGGCTGGAGCTGCTTCG
	EcoCFT073-pri-4c	GTATTTTTCTTTACTCAGGTAGACAGGGTAGACAGCCATTGCAACAAATTATAAAAACGCCATATGAATATCCTCCTTA
**CFT073 Δc1499**	EcoCFT073-cap-1m	ACCTGAATCGCGGGGATATTGCGGAATTTAAATTCAGTCCGCTGTTTACCACGCTGTTTTGTGTAGGCTGGAGCTGCTTCG
	EcoCFT073-cap-2c	AATATCTGCCGGAACCGGCTGCGGTGCGCTGTGCGTCTGCACGTACTCAATCGCCGGATCCATATGAATATCCTCCTTA
**CFT073 Δc1500**	EcoCFT073-Ac1500-1m	GGGGGATTCTATGGACACTCCATACATTGAGTTATTTGCAGGCAGTCAGCAGGTCGCCACTGTGTAGGCTGGAGCTGCTTCG
	EcoCFT073-Ac1500-2c	GTTCCCCCTGCTTATCGGGCCAGCGGCTGAACGGATACGCCAGAACCCGCAAAGGCGGCGCATATGAATATCCTCCTTA
**CFT073 c1499::*cat***	EcoCFT073-adjustchlor-5m	GCGGCGCAGGGTATGAATCAGGTTCACGGGGGTGAATAATGACAGTAAAAGCAATGGCACGGCGCGCCTACCTGTGACGG
	EcoCFT073-adjustchlor-6c	TCAATCGCCGGATCTCCGTCCTCAATCCAGTTTTTCGGGTAAAACATGTTTTGTGTTGCGGGAATAGGAACTTCATTTAAATGGC
**CFT073 ∆cos.1**	CFT073-AcosN-9m	ACAAGGCCGGAGAAATCCGGCTTTTTTTATGGGTCCTTTCCGGCATATGGACCCGTTACGGTCCATATGAATATCCTCCTTAG
	CFT073-AcosN-10c	TAATCAAGTGGTGGTGGTGTCACCTTCACGGAAAAACGTCATAAATAGCGAAAACCCGCGTGTGTAGGCTGGAGCTGCTTCG
**CFT073 ∆cos.2**	CFT073-AcosN-11m	AACTTGCAGAGAAACCGGACATGGATCCCGAAAAATTTTCATAAATAGTGAAAACGCGCGGTCCATATGAATATCCTCCTTAG
	CFT073-AcosN-12c	CCCCCATCGTTCTGATAGTCTGGTGTCGTCGGGTCCTTCCTGGAATTATGGCCCGTTACGTGTGTAGGCTGGAGCTGCTTCG
**CFT073 ∆phi1**	EcoCFT073-phi1-1m	GTAAACGGGTTAATGAAAACAATAAGTTAGAGTAAAAACAATAAGTTGGGTTGGTGGTCATGTGTAGGCTGGAGCTGCTTC
	EcoCFT073-phi1-2c	GCCGTTTAAAATCATGATGTTAGAAGCACTGTTTTTTAACGATGGCGACAAATTGGCGGCCATATGAATATCCTCCTTA
**CFT073 ∆phi2**	EcoCFT073-phi2-1m	GCGGGACACTTTCAGGATTTTGAATTTATTCAATTTTTCCCTTTCTCCAAAATCTCTCCATGTGTAGGCTGGAGCTGCTTC
	EcoCFT073-phi2-2c	TTAGATGATTAATCCTTTATATTCAATGCATTAAGTTTGTTTTTGAGTGGTTTTGCCGTTCATATGAATATCCTCCTTA
**CFT073 ∆phi4**	EcoCFT073-phi4-1m	TGATGTAAAATCTTCCCCAAAACCCTTCCCCAAAACTGGCTATTTTCTATGCTGTTTTGATGTGTAGGCTGGAGCTGCTTC
	EcoCFT073-phi4-2c	ATAAAAATTATGTATTCAACTCGCTGATATTTAATCATAAATATTATTTTCTTGTCTTTGCATATGAATATCCTCCTTA
**CFT073 ∆phi5**	EcoCFT-phi5-7m	GTGAGATGCTTTACGTCTTCCAAGCCCCCTTCCTTGCCGTAAATGTGTGTAGGCTGGAGCTGCTTC

	EcoCFT-phi5-8c	GGCTTTTTTTGTGATATTGCTGGTCGTATTACCGTCATATTCC TTCATATGAATATCCTCCTTA
**phi80 *ΔterS***	Ecophi80-AterS-1m	TAAAAACGGCTAAATAGCCAGAGGGCGTTTCCTTTCTCTGTT TTTGTGTATGGAGTGAGCTGTGTAGGCTGGAGCTGCTTCG
	Ecophi80-AterS-2c	CGATATATTCACTCAGCAACCCCGGTATTATTTCATCCAGCG CAGCTGCTTTGTTCATGGCATATGAATATCCTCCTTA
**IHE3034 RS04635-RS04640::*cat***	IHE3034-adjustchlor-1m	TTATTGAACAAAACTGAGAAACGACATGAAAGCGTCATAAATC GCCATTTTAGATGATTAGGCGCGCCTACCTGTGACGG
	IHE3034-adjustchlor-2c	CACGAACAATATCGAACAACCGCGAACAAAGATCAACACGCT TTAATCATTATCATTTTCGGAATAGGAACTTCATTTAAATGGC
**O42 1749::*cat***	O42-adjustchlor-1m	CATGGAGCAATCAGCCGATACAGAACAGTAAAGCCGATAACT TGTTAATCACTCGCAGCAGGCGCGCCTACCTGTGACGG
	O42-adjustchlor-2c	TAATTAAATGATTTGCACTACAAAAAGTGCCATAAATCGCAAA ATGACTACACCAGTGACTACACCGTTCGGAATAGGAA
**18098 ORF17-ORF18::*cat***	18098-chlor-1m	GCGCGAAAAATACTTACCAGTGGAAGAGGCCGGAGCGGTTA CGGCGGTAACTGGTTGATAGGCGCGCCTACCTGTGACGG
	18098-chlor-2c	CATTAACTACACCGTTCCACGAACAATATCGAACAACCACGA ACAAAGATGGAATAGGAACTTCATTTAAATGGC

Plasmid	Oligonucleotides	Sequence (5'-3')
**pJP1285**	EcoCFT073-3mHindIII	CCCAAGCTTTGGATGCTGATAATTACCACG
	EcoCFT073int-1cSphI	ACATGCATGCCAAAAAAAATTATATTTCTCTGGC
**pJP1286**	EcoCFT073-3mHindIII	CCCAAGCTTTGGATGCTGATAATTACCACG
	EcoCFT073-2cSphI	ACATGCATGCATCGCTCAGTTTGTTAAGTGC
**pJP1287**	EcoCFT073-1mHindIII	CCCAAGCTTGTACCCTGAATGATAAACACG
	EcoCFT073int-1cSphI	ACATGCATGCCAAAAAAAATTATATTTCTCTGGC
**pJP2027**	EcClCFT073-rep-1mPstI	AACTGCAGAACCAATGCATTGGATCCAGAAGCTGAA AACCATG
	EcClCFT073-rep-2cNotI	ATAAGAATGCGGCCGCTAAACTATTTAAGTTTTCCTG ATGTGCGC
**pJP2028**	EcClCFT073-rep-1mPstI	AACTGCAGAACCAATGCATTGGATCCAGAAGCTGAA AACCATG
	EcClCFT073-rep-3cNotI	ATAAGAATGCGGCCGCTAAACTATCAAATTATAAAAA CGCGTCGC
**pJP2029**	EcClCFT073-rep-1mPstI	AACTGCAGAACCAATGCATTGGATCCAGAAGCTGAA AACCATG
	EcClCFT073-rep-4c	CTCACTGTCCGCCATCTGCG
	EcClCFT073-rep-5m	CGCAGATGGCGGACAGTGAGGTGACGGTGATGAAG CACTGG
	EcClCFT073-rep-2cNotI	ATAAGAATGCGGCCGCTAAACTATTTAAGTTTTCCTG ATGTGCGC
**pJP2030**	Eco-cos-lambda-3mE	CCGGAATTCGTTGTTGTTCTGCGGGTTCTG
	Eco-cos-lambda-4cH	CCCAAGCTTCTCAATAGTTCCTGGCTGGAG
**pJP2031**	Eco-cos-phi80-1mE	CCGGAATTCACCGTCAGTGCCGATAAGTTC
	Eco-cos-phi80-2cH	CCCAAGCTTTCGCGTGCGGAATACCATTCG
**pJP2032**	Eco-cos-phi4CFT-3mE	CCGGAATTCTGATTCGTTGGTCCTGGTAGC
	Eco-cos-phi4CFT-4cH	CCCAAGCTTGATATCAGTTTCCCTCTGCGC
**pJP2033**	Eco-cos-islaCFT-3mE	CCGGAATTCCGTGCCTTGTATCGATGTTGG
	Eco-cos-islaCFT-4cS	ACCGGTCGACTCACTGGAGTACTCGTCACGC
**pJP2034**	Eco-cos-islaCFT-5mS	ACGCGTCGACAGAACATCGATTTGATGAAGG

	Eco-cos-islaCFT-6cE	CCGGAATTCGCGATTGTAGGCCAGCATTTC
**pJP1288**	EcoCFT073rep-1mS	ACGCGTCGACGTAACCACATTGCCAATAGCC
	EcoCFT073rep-3cB	CGCGGATCCTTAGTGAACTGCCTCGC
**pJP1289**	EcoCFT073rep-1mS	ACGCGTCGACGTAACCACATTGCCAATAGCC
	EcoCFT073rep-2cB	CGCGGATCCATCTGTGCTTAACACA
**pJP1290**	EcoCFT073rep-1mS	ACGCGTCGACGTAACCACATTGCCAATAGCC
	EcoCFT073rep-5cB	CGCGGATCCCTCATGCGTTGGGGATAACG
**pJP1291**	EcoCFT073rep-1mS	ACGCGTCGACGTAACCACATTGCCAATAGCC
	EcoCFT073rep-2c	ATCTGTGCTTAACACA
	EcoCFT073rep-4m	TGTGTTAAGCACAGATGCAGTTCACTAATCAGG
	EcoCFT073rep-5cB	CGCGGATCCCTCATGCGTTGGGGATAACG
**pJP2037**	EcoCFT073-alpA-1mE	CCGGAATTCCACAACCCAACCCAATAATCC
	EcoCFT073-alpA-3cK	GGGGTACCGCTGTGTGCCGCCAGCCTG
**pJP2038**	EcoCFT073-c1483-1mE	CCGGAATTCGTAACCACATTGCCAATAGCC
	EcoCFT073-alpA-11cH	CCCAAGCTTTCAGGCCTGTAAGTTGCTGAC
**pJP2039**	EcoCFT073-2mE	CCGGAATTCGCGTTTCTGCCAGTGACGTTC
	EcoCFT073-3cH	CCCAAGCTTTGTTTGCACGTCTGTAGCAAG

**Southern Blot**	**Oligonucleotides**	**Sequence (5'-3')**
**PmCI172 probe**	Pm-172-1m	TATACCCGTTCAACATAACCC
	Pm-172-2c	CTGCGACGTATGGAACACAGC
**Phage Pm172 probe**	Pm-172-3m	CAGAATGGTCACGTCATACCG
	Pm-172-4c	GCCTTGGGGGATACGAATTTG
**EcCICFT073 probe**	EcoCFT073-c1501-1m	TTTGCGGGTTCTGGCGTATCC
	EcoCFT073-c1501-2c	AGCCGGATTTCTCCGGCCTTG

**Excision, circularisation and integration**	**Oligonucleotides**	**Sequence (5'-3')**
**Excision**	EcoCFT073-1m	GTACCCTGAATGATAAACACG
	EcoCFT073-4c	TCAACGGGTTGATACTTTACG
**Circularisation**	EcoCFT073-3m	TGGATGCTGATAATTACCACG
	EcoCFT073-2c	ATCGCTCAGTTTGTTAAGTGC
**Integration**	EcoCFT073-1m	GTACCCTGAATGATAAACACG
	EcoCFT073-2c	ATCGCTCAGTTTGTTAAGTGC

Table S7. Strains used in this study.

Strain	Description	Reference
Pasteurella multocida		
JP13738	Pm86. Natural isolate	This work
JP13739	Pm172. Natural isolate	This work
Escherichia coli		
CFT073	Natural isolate	
C600	Laboratory strain	ATCC 23738
594	Laboratory strain	
WG5	Laboratory strain	
DH5α	Laboratory strain	
JP9293	CFT073 c1501::*tet*A	This work
JP9296	JP9293 Δø1::*cat*	This work
JP9297	JP9293 Δø2::*cat*	This work
JP9298	JP9293 Δø4::*cat*	This work
JP12731	JP9293 Δø5::*cat*	This work
JP9794	JP9293 Δ*int*	This work
JP9795	JP9293 Δ*alpA*	This work
JP9796	JP9293 Δ*pri-rep*	This work
JP9797	JP9293 Δc1499	This work
JP15258	JP9293 Δc1500	This work
JP9802	CFT073 *lacZ*::*tet*A	This work
JP13343	CFT073 c1499::*cat*	This work
JP13408	IHE3034 RS04635-RS04640::*cat*	This work
JP13409	O42 1749::*cat*	This work
JP15585	18098 ORF17-ORF18::*cat*	This work
JP13694	CFT073 c1499::cat Δø4	This work
JP14200	DH5alpha pJP1285	This work
JP14201	DH5alpha pJP1287	This work
JP14202	DH5alpha pJP1286	This work
JP13734	DH5alpha pJP2027	This work
JP13735	DH5alpha pJP2028	This work
JP13736	DH5alpha pJP2029	This work
JP10400	C600 lambda lysogen	This work
JP13503	JP10400 EcCICFT073-c1499::*cat*	This work
JP15111	JP10400 EcCICFT073-c1499::cat Δ*cos*.1	This work
JP15112	JP10400 EcCICFT073-c1499::cat Δ*cos*.2	This work
JP12507	594 phi80 lysogen	This work
JP13888	JP12507 EcCICFT073-c1499::*cat*	This work
JP15113	JP12507 EcCICFT073-c1499::cat Δ*cos*.1	This work
JP15114	JP12507 EcCICFT073-c1499::cat Δ*cos*.2	This work
JP15116	JP12507 EcCICFT073-c1499::cat Δ*cos*.1 Δcos.2	This work

Strain	Description	Reference
JP15175	JP12507 Δ*terS* EcCICFT073-c1499::*cat*	This work
JP12712	JP10400 pET28a	This work
JP12713	JP10400 pJP2030	This work
JP12714	JP10400 pJP2032	This work
JP12715	JP10400 pJP2033	This work
JP15178	JP10400 pJP2034	This work
JP14701	JP12507 pET28a	This work
JP14702	JP12507 pJP2031	This work
JP15179	JP12507 pJP2032	This work
JP14703	JP12507 pJP2033	This work
JP15180	JP12507 pJP2034	This work
JP12677	C600 EcCICFT073-c1501::*tet*A	This work
JP15181	C600 EcCICFT073-c1501::*tet*A Δc1499	This work
JP15182	C600 EcCICFT073-c1501::*tet*A Δc1500	This work
JP16616	C600 EcCICFT073-c1499::*cat* pBAD18	This work
JP16617	C600 EcCICFT073-c1499::*cat* pJP2037	This work
JP16618	C600 EcCICFT073-c1499::*cat* Δ*int* pJP2037	This work
JP16619	C600 EcCICFT073-c1499::*cat* Δ*pri* pJP2037	This work
JP9542	DH5α pJP1288	This work
JP9543	DH5α pJP1289	This work
JP9544	DH5α pJP1290	This work
JP9730	DH5α pJP1291	This work
JP15799	DH5α pBAD18 pJP2038	This work
JP15800	DH5α pJP2037 pJP2038	This work
JP15801	DH5α pBAD18 pJP2039	This work
JP15802	DH5α pJP2037 pJP2039	This work
JP15218	C600 pJP1290	This work
JP13040	JP10400 pJP1290	This work
JP16582	594 pJP1290	This work
JP13043	JP12507 pJP1290	This work
JP17362	JP12507 Δ*terS* pJP1290	This work

Table S8. Plasmid used in this study.

Plasmid	Description	Reference
**pMAK700**	Cm^R. Thermosensitive plasmid in *E. coli*	(Hamilton *et al.*, 1989)
**pK03 Blue**	Cm^R. Thermosensitive plasmid in *E. coli*	(Solano *et al.*, 2009)
**pGE593**	Amp^R. Lac reporter plasmid	(Eraso and Weinstock, 1992)
**pRW224**	Tet^R. Lac reporter plasmid	(Lodge *et al.*, 1992)
**pBAD18**	Amp^R. Expression vector	(Guzman *et al.*, 1995)
**pET28a**	Km^R. Expression vector	(Novagen)
**pKD46**	Amp^R. Thermosensitive plasmid with Red system of lambda phage	(Datsenko and Wanner, 2000)
**pCP20**	Amp^R. Thermosensitive plasmid in *E. coli* with FLP recombinase	(Datsenko and Wanner, 2000)
**pJP1285**	pMAK700 *int-att$_P$* EcCICFT073	This work
**pJP1287**	pMAK700 *int-att$_L$* EcCICFT073	This work
**pJP1286**	pMAK700 *Δint-att$_P$* EcCICFT073	This work
**pJP2027**	pK03 Blue *pri-ori* EcCICFT073	This work
**pJP2028**	pK03 Blue *pri-Δori* EcCICFT073	This work
**pJP2029**	pK03 Blue *Δpri-ori* EcCICFT073	This work
**pJP2030**	pET28a cos lambda	This work
**pJP2031**	pET28a cos phi80	This work
**pJP2032**	pET28a cos phi4 CFT073	This work
**pJP2033**	pET28a cos.1 EcCICFT073	This work
**pJP2034**	pET28a cos.2 EcCICFT073	This work
**pJP1288**	pGE593 *alpA-alpA-lacZ* EcCICFT073	This work
**pJP1289**	pGE593 *ΔalpA-alpA-lacZ* EcCICFT073	This work
**pJP1290**	pGE593 *alpA-lcd_like-lacZ* EcCICFT073	This work
**pJP1291**	pGE593 *ΔalpA-lcd_like-lacZ* EcCICFT073	This work
**pJP2037**	pBAD18 *alpA* EcCICFT073	This work
**pJP2038**	pRW224 promoter *alpA-lacZ* EcCICFT073	This work
**pJP2039**	pRW224 promoter *int-lacZ* EcCICFT073	This work

References

Datsenko KA, Wanner BL. (2000). One-step inactivation of chromosomal genes in *Escherichia coli* K-12 using PCR products. *Proc Natl Acad Sci USA* **97**: 6640–6645.

Guzman LM, Belin D, Carson MJ, Beckwith J. (1995). Tight regulation, modulation, and high-level expression by vectors containing the arabinose PBAD promoter. *J Bacteriol* **177**: 4121–4130.

Hamilton CM, Aldea M, Washburn BK, Babitzke P, Kushner SR. (1989). New method for generating deletions and gene replacements in *Escherichia coli*. *J Bacteriol* **171**: 4617–4622.

Lodge J, Fear J, Busby S, Gunasekaran P, Kamini NR. (1992). Broad host range plasmids carrying the *Escherichia coli* lactose and galactose operons. *FEMS Microbiol Lett* **74**: 271–276.

Solano C, García B, Latasa C, Toledo-Arana A, Zorraquino V, Valle J, *et al.* (2009). Genetic reductionist approach for dissecting individual roles of GGDEF proteins within the c-di-GMP signaling network in Salmonella. *Proc Natl Acad Sci USA* **106**: 7997–8002.
