# Supplementary material for: Phage-inducible chromosomal islands are ubiquitous within the bacterial universe
Source: ISME J. 2018 Jun 6;12(9):2114–28. doi: 10.1038/s41396-018-0156-3 (PMC6092414; doi:10.1038/s41396-018-0156-3)
Supplement: Supplementary file 2 — Supplemtary figures [file 41396_2018_156_MOESM2_ESM.pdf]

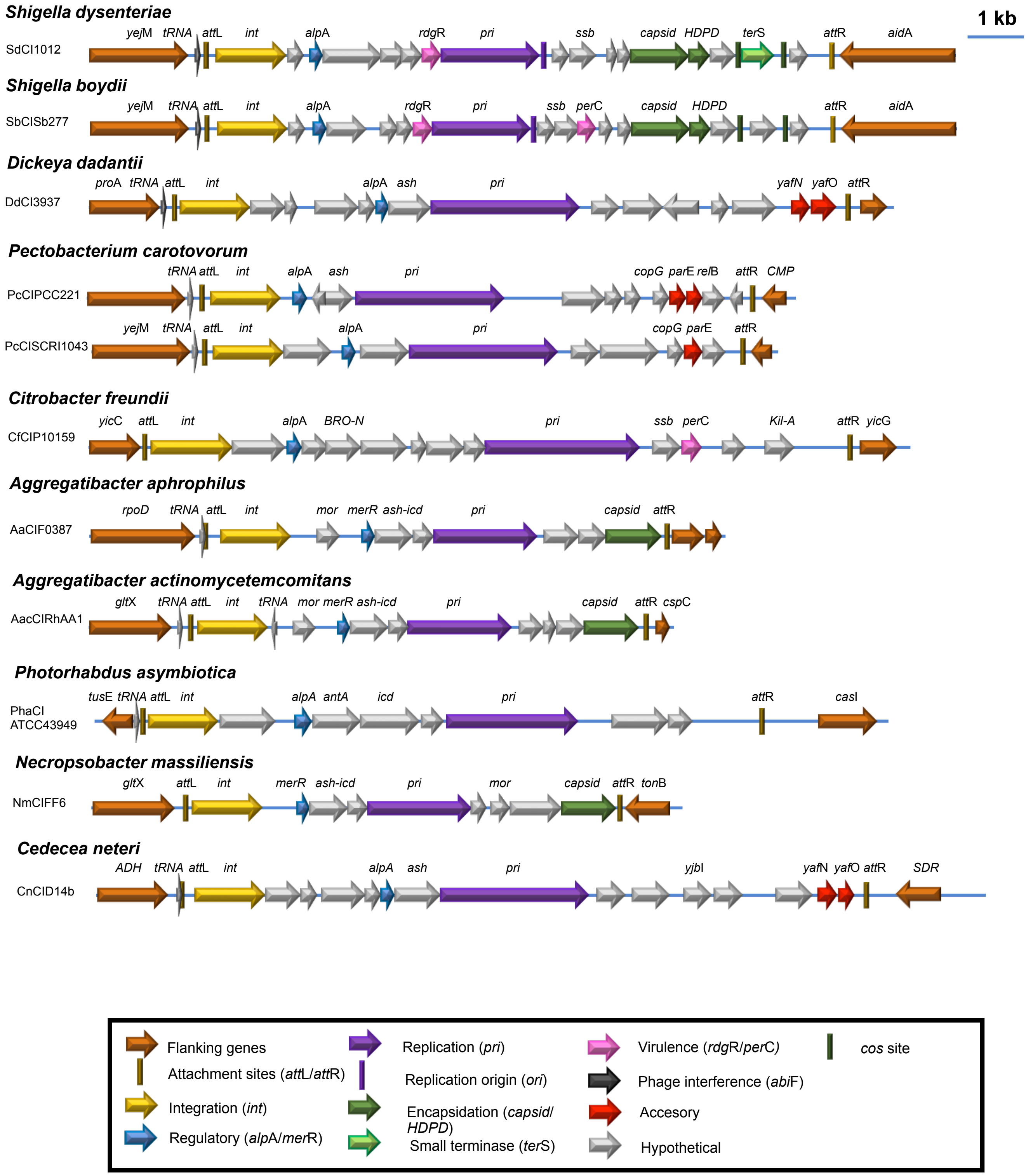

Figure S1. Phage-inducible chromosomal island genomes. Genomes are aligned according to the prophage convention, with the integrase gene (*int*) at the left end. Genes are coloured according to their sequence and function: *int* is yellow; transcription regulator (*alp*A or *mer*R) is dark blue; replication genes are purple; encapsidation genes are green, with the terminase small subunit gene (*ter*S) in light green; superantigen and other virulence genes are pink; genes encoding putative phage resistance proteins are black; other accessory genes are red; genes encoding hypothetical proteins are white.

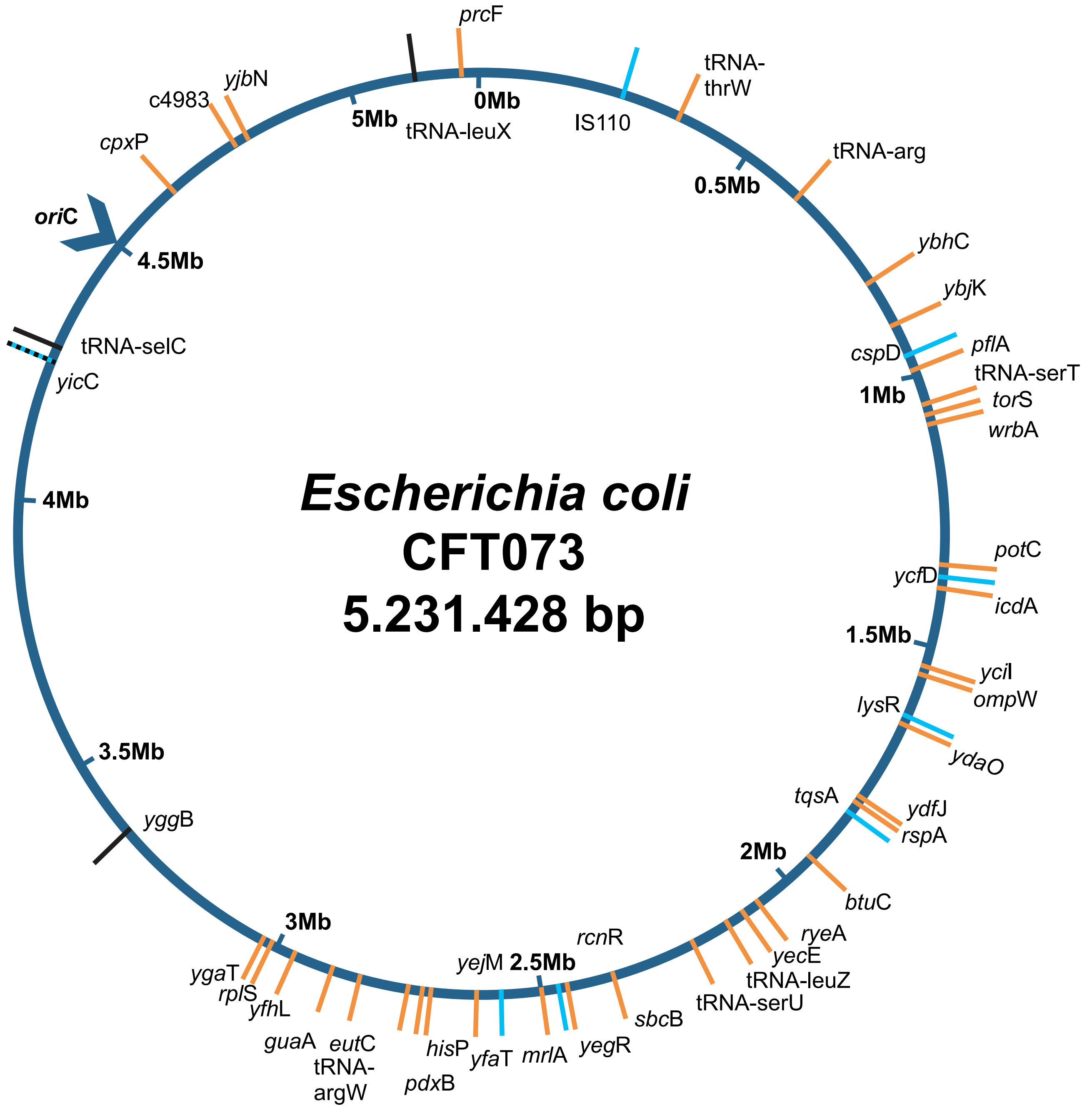

Figure S2. Locations of the *att* sites in *Escherichia coli*. Phage *att* sites are indicated in orange, PICI *att* sites are in blue and P4 *att* sites are in black. The gene next to the *int* encoded by the different elements is indicated; in the outer are the genes flanking the phage *att* sites while in the inner are those genes flanking either the EcCI or the P4 *att* sites.

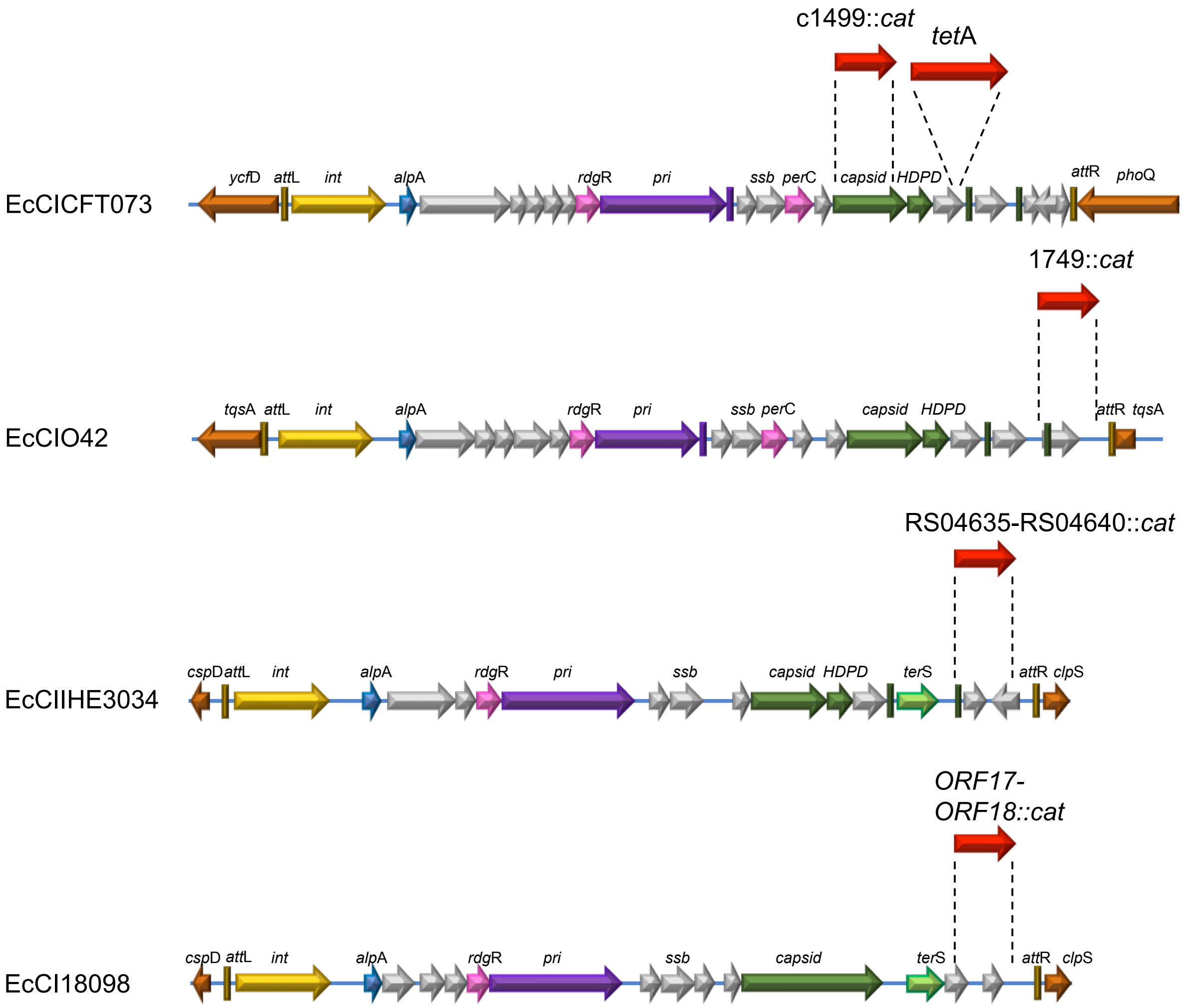

Figure S3. Location of the *tet*A and *cat* markers in the EcCI genomes.

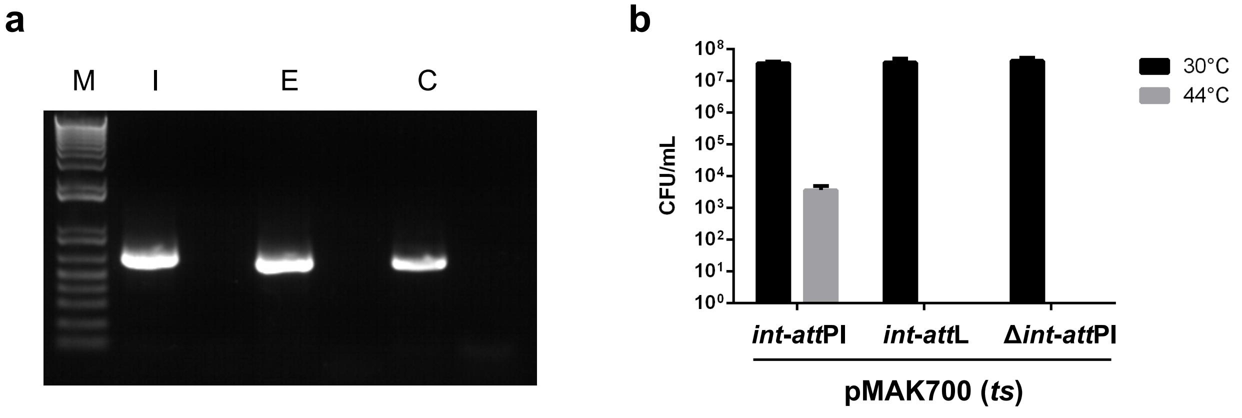

Figure S4. Testing EcClCFT073 *int* function. (a) Detection of excision and circularization. DNA from *E. coli* CFT073 was extracted and PCR-amplified using specific primers recognizing the external and internal sequence of the element (integration: I), primers recognizing the chromosomal flanking sequences (excision: E) or PCR amplified using a pair of primers that hybridize divergently at both termini of the island (circularization: C). M: molecular weight marker. **(b)** pMAK700 derivative plasmids *int-att*$_{PI}$; *int-att*$_{L}$ and Δ*int-att*$_{PI}$. Strains carrying the different plasmids were grown and tested for integration in DH5α (*rec*A-mutant) on selective agar at the restrictive (44ºC) temperature.

a

***cos* phage Lambda**

***cos*Q** = TTTACGGGTCCTTTCC

***cos*N** = GGGCGGCGACCT

***cos*B** =

R3 = AAGGCGTTTCCGTTCT

R2 = AGAAAGGAAACGACAG

R1 = CTGTCGTTTCCTTTCT

Lambda

TTTACGGGTCCTTTCCGGTGATCCGACAGGTTACGGGGCGGCGACCTCGCGGGTTTTCGCTATTTATGAAAATTTTCCGGTTTAAGGCGTTTCCGTTCTTCTTCGTCATA
ACTTAATGTTTTTATTTAAAATACCCTCTGAAAAGAAAGGAAACGACAGGTGCTGAAAGCGAGGCTTTTTGGCCTCTGTCGTTTCCTTTCTCTGTTTTTGTCCGTGGAAT
GAACAATGGAAGTCAACAAAAAGCAGCTGGCTGACATTTTCGGTGCGAGTATCCGTACCATTCAGAACTGGCAGGAACAGG

phi80

TTTGCGGGTCCTTTCCGGCGATCCGCCTTGTTACGGGGCGGCGACCTCGCAGATTCTCGCTATTTATGAAAATTTTCAGGCATTTGCCGTTTCCGTTCTTCTTCTCGCTA
ATTCATTGTTTTAACTGTAAACACCCCCTGAAAAGAAAGGAAATGATAAGCCTTAAAAACGGCTAAATAGCCAGAGGGCGTTTCCTTTCTCTGTTTTTGTGTATGGAGTG
AGCTATGGAGGTCAACAAAAAGCGTCTTTCTGAAATATTTGGGGTCAGCGTGCGAACCATTCAGAACTGGCAGGATCAGGG

CFT073 cos.1

TTTATGGGTCCTTTCCGGCATATGGACCCGTTACGGGGCGGCGACCTCGCGGGTTTTCGCTATTTATGACGTTTTTCCGTGAAGGTGACACCACCACCACTTGATTAATA
TTTAACCATGCAGTTAAGGTAACATTATGATTGATAAAGCTTGTTTTGTAAGTCAGCAGGAAATAGCTGAACATTTCAAGGTTAACAGAACCACTATTCGCGCATGGACC
AAACAGGGGATGCCGTATCTTAATGCGGATCGCGGAAAGTCTGGCGGTTATCACATCGGGCATACATTGCTTTGGTCTTCA

CFT073 cos.2

TCGTCGGGTCCTTCCTGGAATTATGGCCCGTTACGGGGCGGCGACCTCGCGCGTTTTCACTATTTATGAAAATTTTTCGGGATCCATGTCCGGTTTCTCTGCAAGTTAAC
CATATGAAAAATATAAAAACATGCTTTCCATGAACCGGACATGCGCAAAAAACAGACACTAAAACCGGACATCGAACCAGTTAACCGAAAGTGTGCACAAATCACATGCA
TTGCTGCGAGTGATTAACAAGTTATCGGCTTTACTGTTCTGTATCGGCTGATTGCTCCATGCTGGCACGGCGAACGCTTAC

phi4 CFT073

TTTTCGGGTCCTTTCCGTCGATCCAACAGGTTACGGGGCGGCGACCTCGCGGTTTTTCACTATTTATGAAAATTTTTCAGGGAAAATCGTGTCGGTACTTCTCGAATATA
ACTTTTTGTTTTTTTTAATATTGCATCCGTAAAGGTCCGACATGAAAGTGTCCGAAAATGCCTTTTTCTGGCGTTTTCATGTCGGGCCTTGTATTTGATAATGGGTTGTT
TTCATGAAGGTTAATAAAAAGAGGCTTGCCGAAATTTTCAACGTGGACCCGCGGACGATTGAACGCTGGCAGTCTCAGGGAC

b

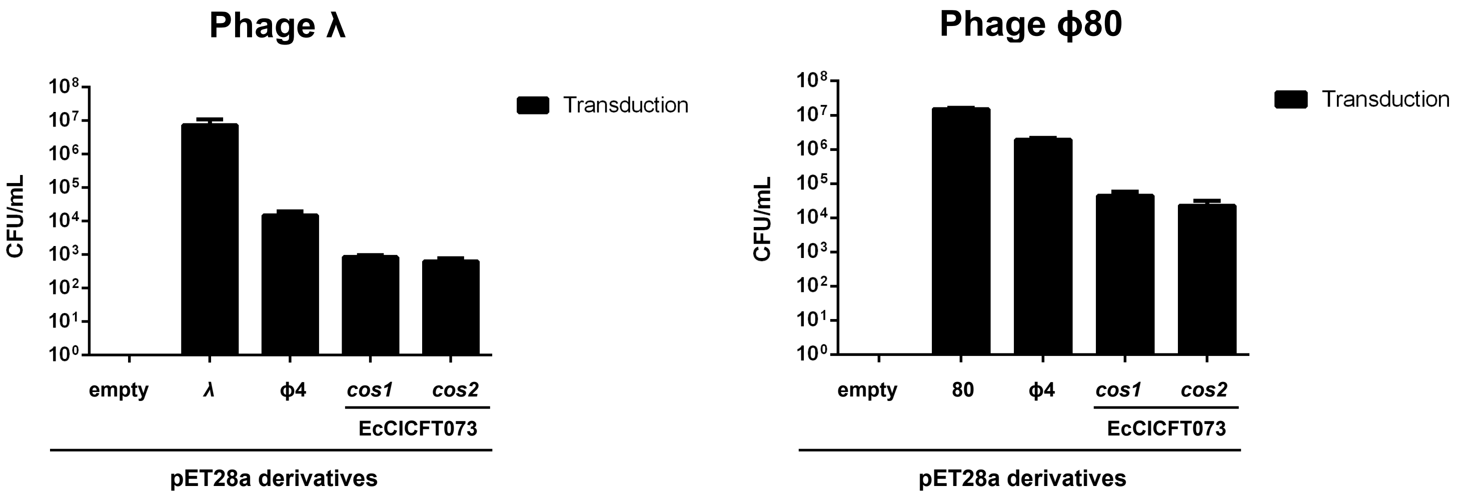

Figure S5. Functionality of the EcCICFT073 *cos* sites. (a) Sequences of the different putative *cos* sites. While all the elements carry similar *cosQ* and *cosN* sequences, the PICI *cosB* sequences are completely divergent. **(b)** pET28a derivative plasmids, containing the different *cos* sequences diagrammed in panel (a), including the λ, ϕ80, phi4 and EcCICFT073 *cos1* or *cos2* sequences were introduced in the lysogenic strains for phages λ or ϕ80. The generated strains were MC induced (2 µg/ml) and the transfer (transduction) of the plasmid analysed.

a

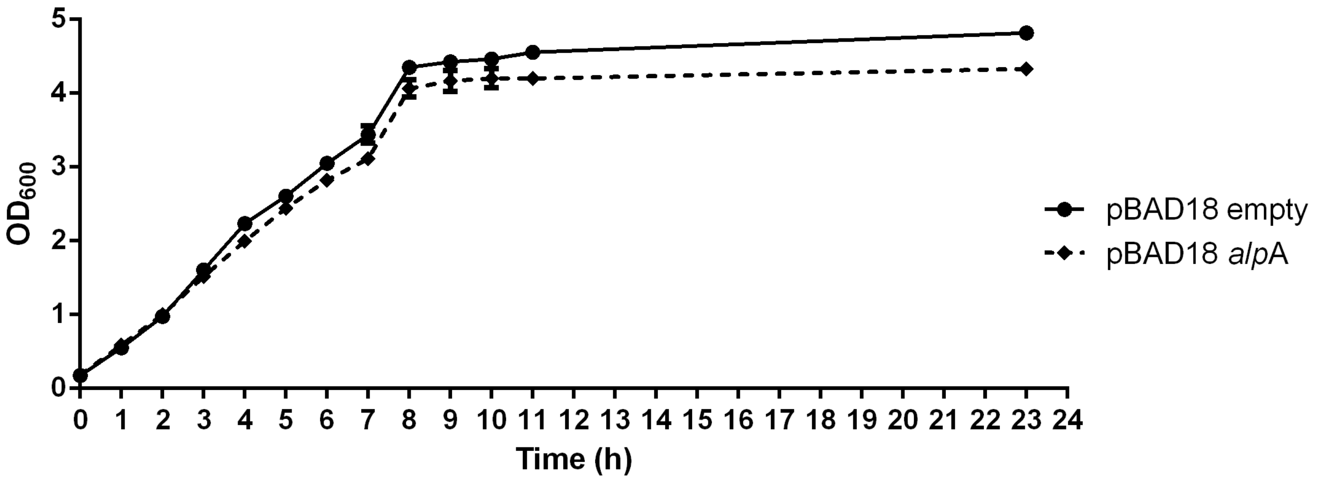

b

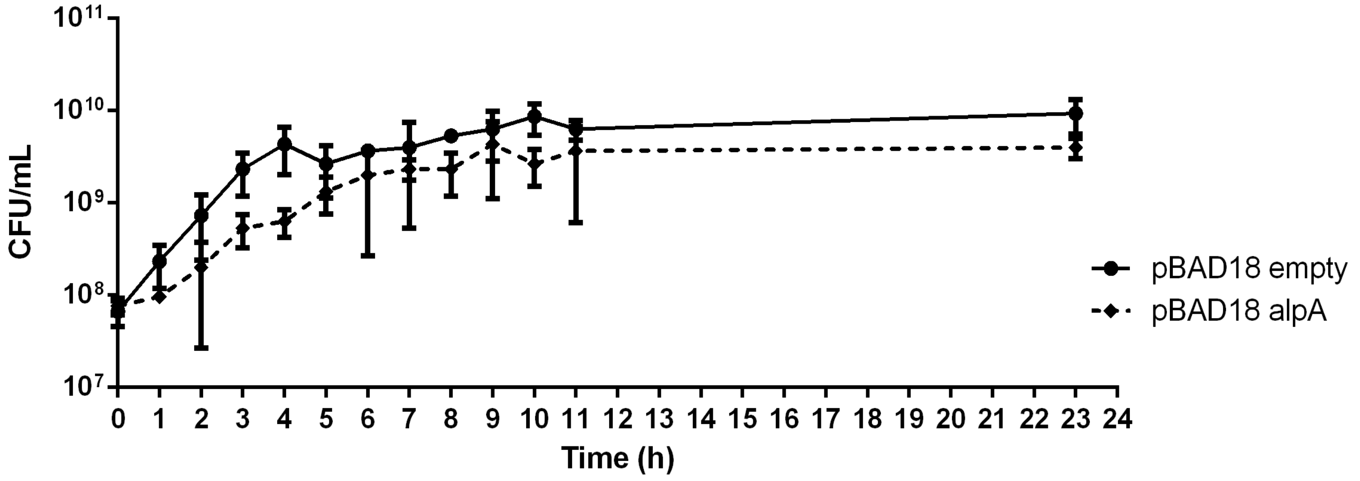

Figure S6. Growth curve of the *E. coli* C600 derivative carrying EcCICFT073 and plasmid pBAD18 or pBAD-*alp*A. The growth curve was done over the course of 24 hours at 30 ºC using OD_{600} measurements **(a)** or CFU/mL counting **(b)**. 0.02% arabinose was added at time 0 to express *alp*A. Each point represents the means of results from three independent experiments.

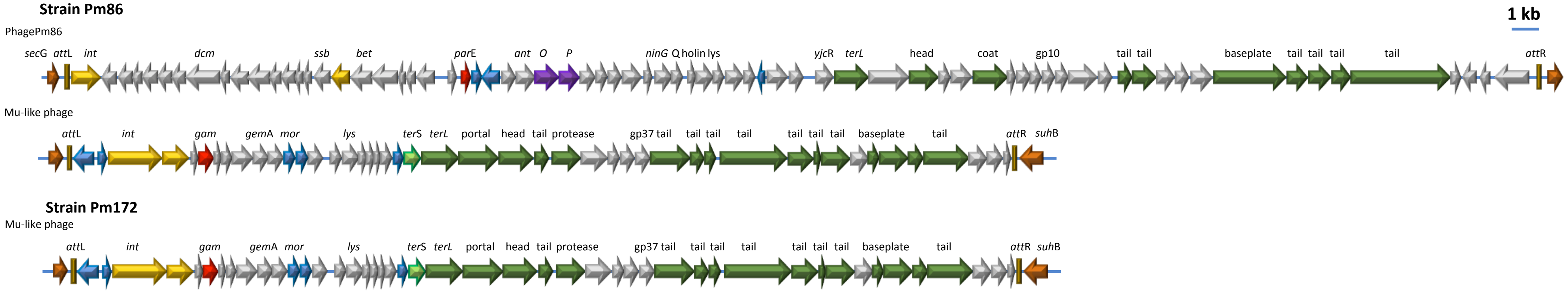

Figure S7. Map of the *P. multocida* phages present in strains Pm86 (MH238467) and Pm172 (MH238466). Genomes are aligned according to the prophage convention, with the integrase gene (*int*) at the left end. Genes are coloured according to their sequence and function: *int* is yellow; transcription regulator is dark blue; replication genes are purple; encapsidation genes are green, with the terminase small subunit gene (*ter*S) in light green; superantigen and other virulence genes are pink; other accessory genes are red; genes encoding hypothetical proteins are white.

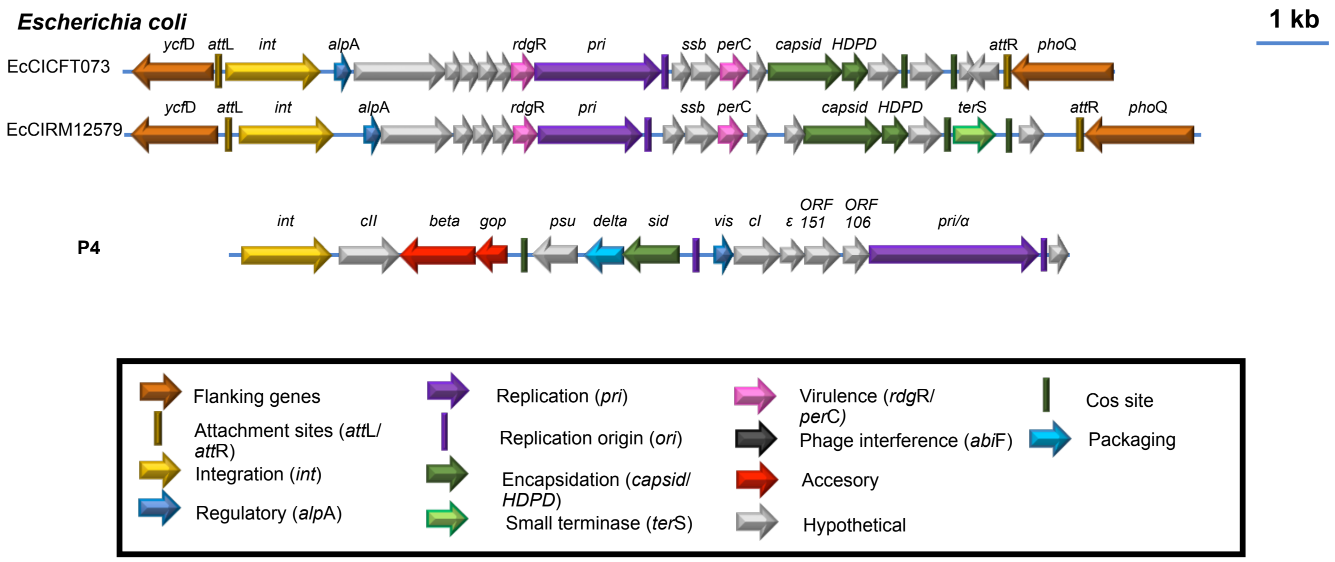

Figure S8. Comparison of PICI and P4 genomes. Genomes are aligned according to the prophage convention, with the integrase gene (*int*) at the left end. Genes are coloured according to their sequence and function: *int* is yellow; transcription regulator (*alp*A or *vis*) is dark blue; replication genes are purple; encapsidation genes are green, with the terminase small subunit gene (*ter*S) in light green; superantigen and other virulence genes are pink; genes encoding putative phage resistance proteins are black; other accessory genes are red; genes encoding hypothetical proteins are white.

a

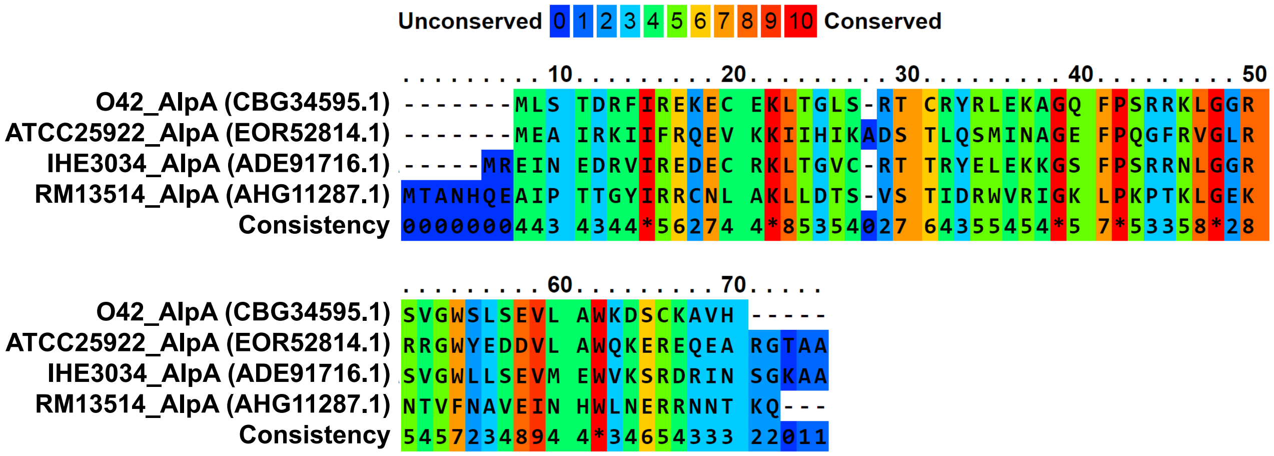

b

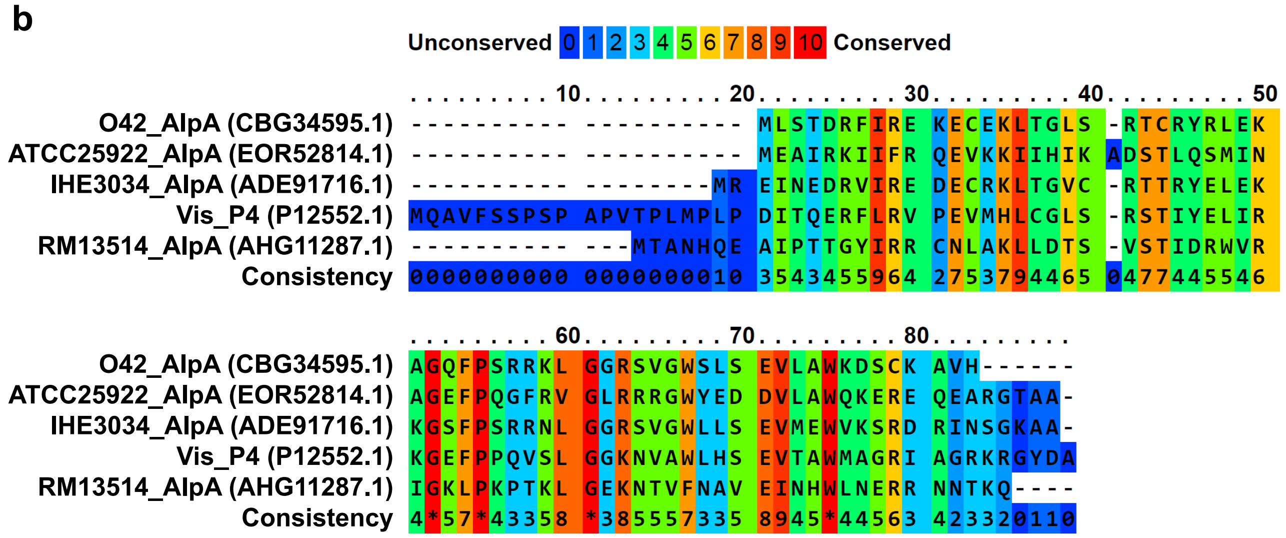

Figure S9. Comparison of different EcCI AlpA and Vis P4 proteins. (a) Alignment generated by PRALINE (matrix BLOSUM62) of AlpA protein sequences from EcCIO42 (CBG34595), EcCIATCC25922 (EOR52814), EcCIIHE3034 (ADE91716), and EcCIRM13514 (AHG11287). **(b)** Alignment generated by PRALINE (matrix BLOSUM62) of AlpA protein sequences from EcCIO42, EcCIATCC25922, EcCIIHE3034, EcCIRM13514 and Vis protein from P4 (P12552). **(c)** Radial phylogenetic tree resulting from alignment of AlpA and Vis sequences using CLC Genomics Workbench. The accession number for the protein sequences are as follow: EcCICFT073 (Identical to EIL17504.1), EcCIRM12579 (AEZ39958), EcCIO42 (CBG34595), EcCIK1516 (EZB64731), EcCIATCC25922 (EOR52814), EcCIED1a (CAR08823.2), EcCIIHE3034 (ADE91716), EcCI11128 (BAI36743), EcCI11368 (BAI26316), EcCIDi14 (identical to EGB43710.1), EcCI224 (Identical to ACB19277), EcCIEC2733.1 (Identical to ERB13914.1), EcCI18098 (Identical to EFJ60051), EcCIRM13514 (AHG11287) and Vis protein from P4 (P12552). The tree was generated using the following parameters; algorithm = UPGMA, distance measure = Jukes-Cantor, bootstrap = 1000 Replicates.

c

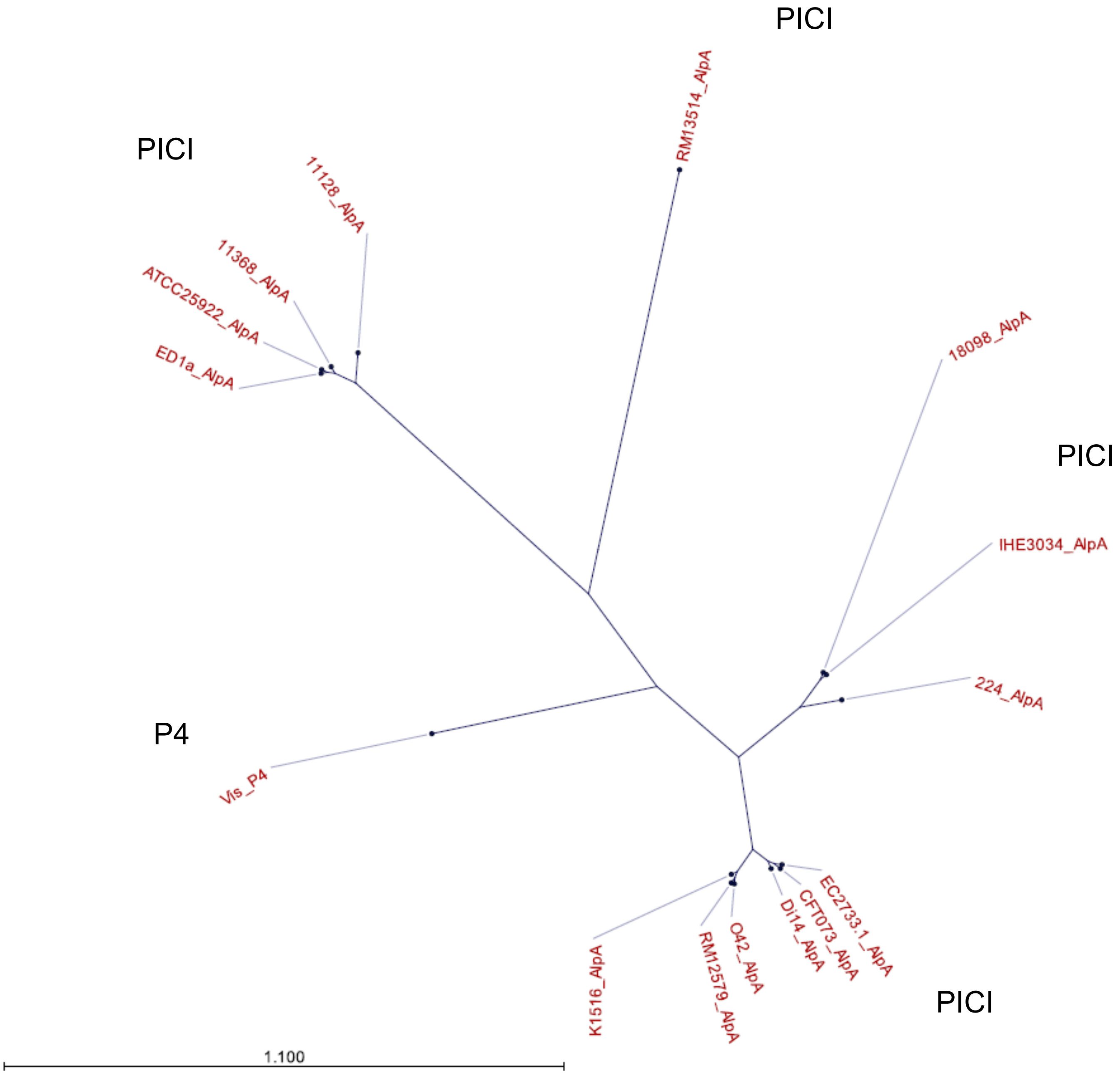
PICI
PICI
PICI
PICI
P4
RM13514_AlpA
11128_AlpA
11368_AlpA
ATCC25922_AlpA
ED1a_AlpA
18098_AlpA
IHE3034_AlpA
224_AlpA
Vis_P4
K1516_AlpA
RM12579_AlpA
O42_AlpA
Di14_AlpA
CFT073_AlpA
EC2733.1_AlpA
1.100

a

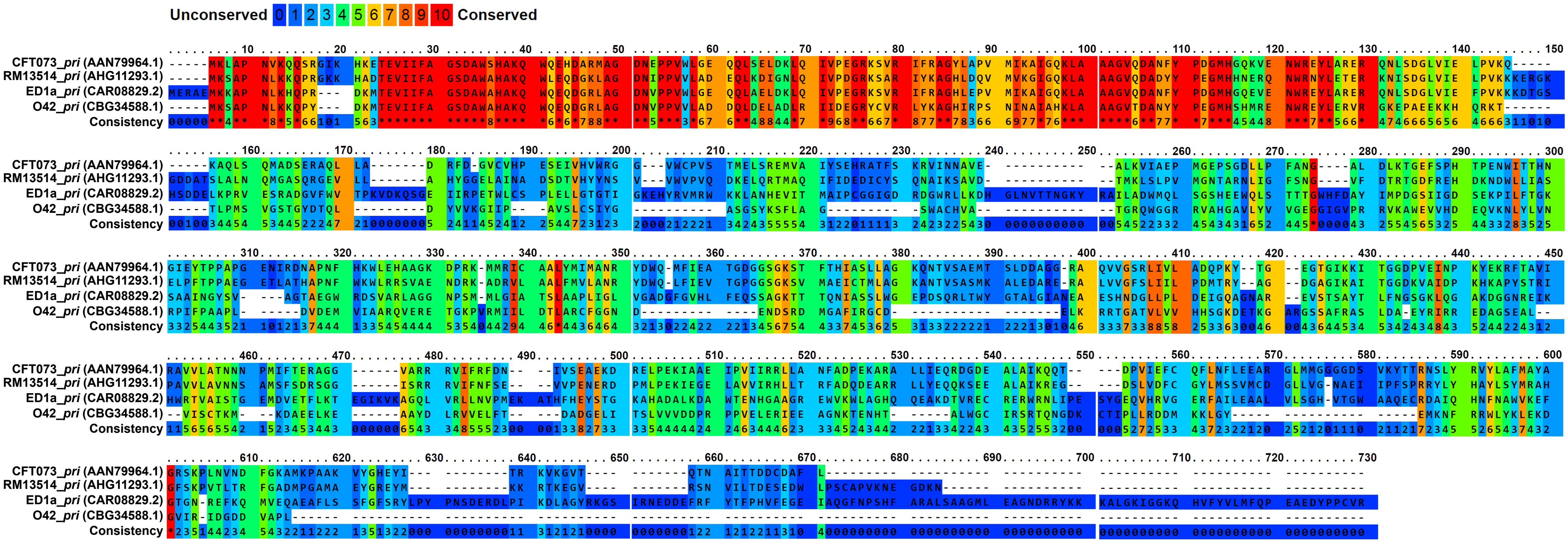

Figure S10. Comparison of different EcCI Pri-Rep and α P4 proteins. (a) Alignment generated by PRALINE (matrix BLOSUM62) of Pri-Rep protein sequences from EcCICFT073 (AAN79964), EcCIRM13514 (AHG11293), EcCIO42 (CBG34588) and EcCIED1a (CAR08829). **(b)** Alignment generated by PRALINE (matrix BLOSUM62) of Pri-Rep protein sequences from EcCICFT073, EcCIRM13514, EcCIO42, EcCIED1a and α protein from P4 (P10277). **(c)** Radial phylogenetic tree resulting from alignment of Pri-Rep and α sequences using CLC Genomics Workbench. The accession number for the protein sequences are as follow: EcCICFT073 (AAN79964), EcCIRM12579 (AEZ39963), EcCIO42 (CBG34588), EcCIK1516 (EZB64739), EcCIATCC25922 (EOR52820), EcCIED1a (CAR08829), EcCIIHE3034 (ADE92165), EcCI11128 (BAI36753), EcCI11368 (BAI26324), EcCIDi14 (AER87766), EcCI224 (Identical to KRR57888), EcCIEC2733.1 (Identical to WP_053285629.1), EcCI18098 (Identical to EQQ34345), EcCIRM13514 (AHG11293) and α protein from P4 (P10277). The tree was generated using the following parameters; algorithm = UPGMA, distance measure = Jukes-Cantor, bootstrap = 1000 Replicates.

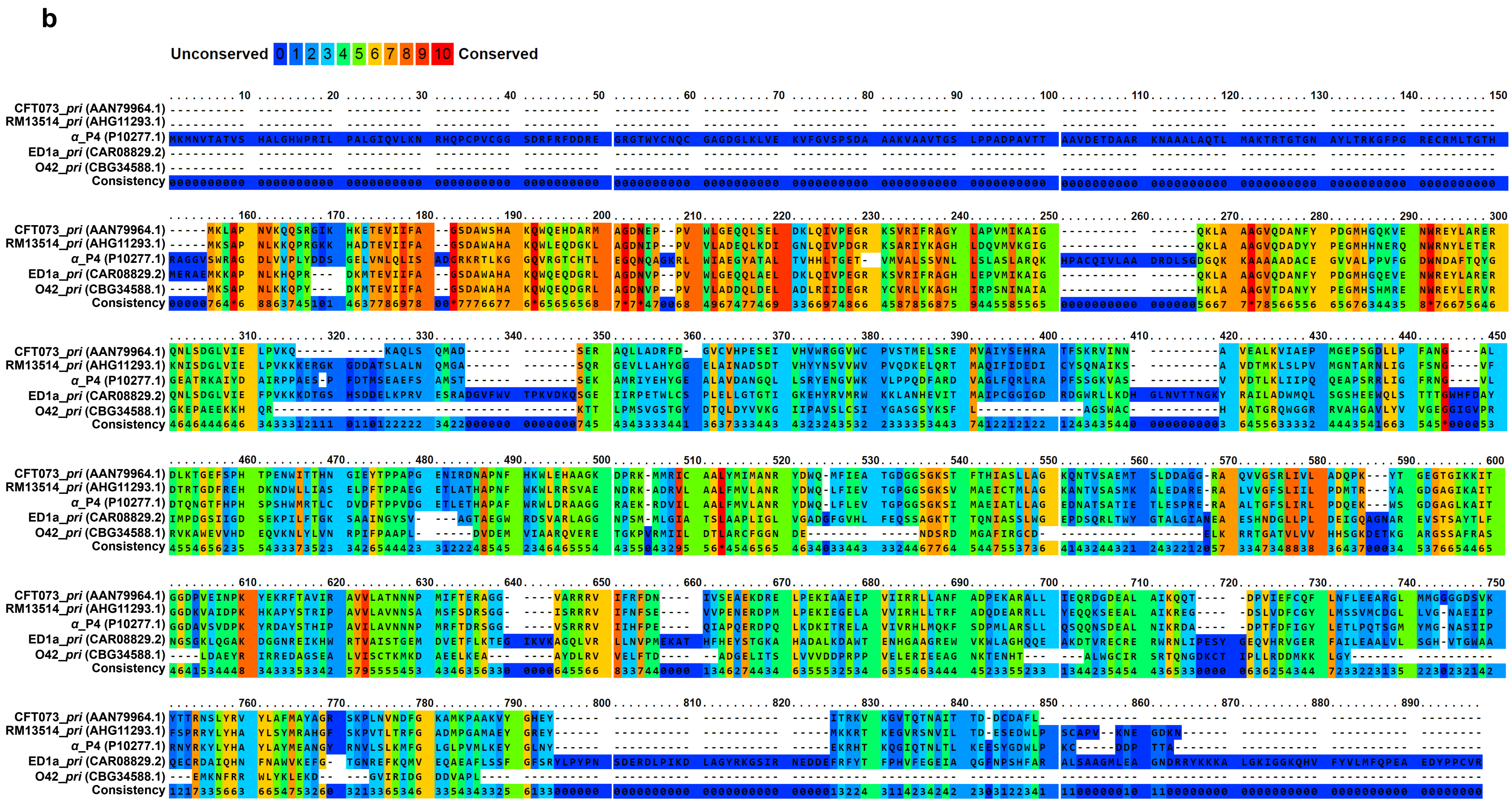
b
Unconserved 0 1 2 3 4 5 6 7 8 9 10 Conserved
CFT073_pri (AAN79964.1)
RM13514_pri (AHG11293.1)
α_P4 (P10277.1)
ED1a_pri (CAR08829.2)
O42_pri (CBG34588.1)
Consistency

c

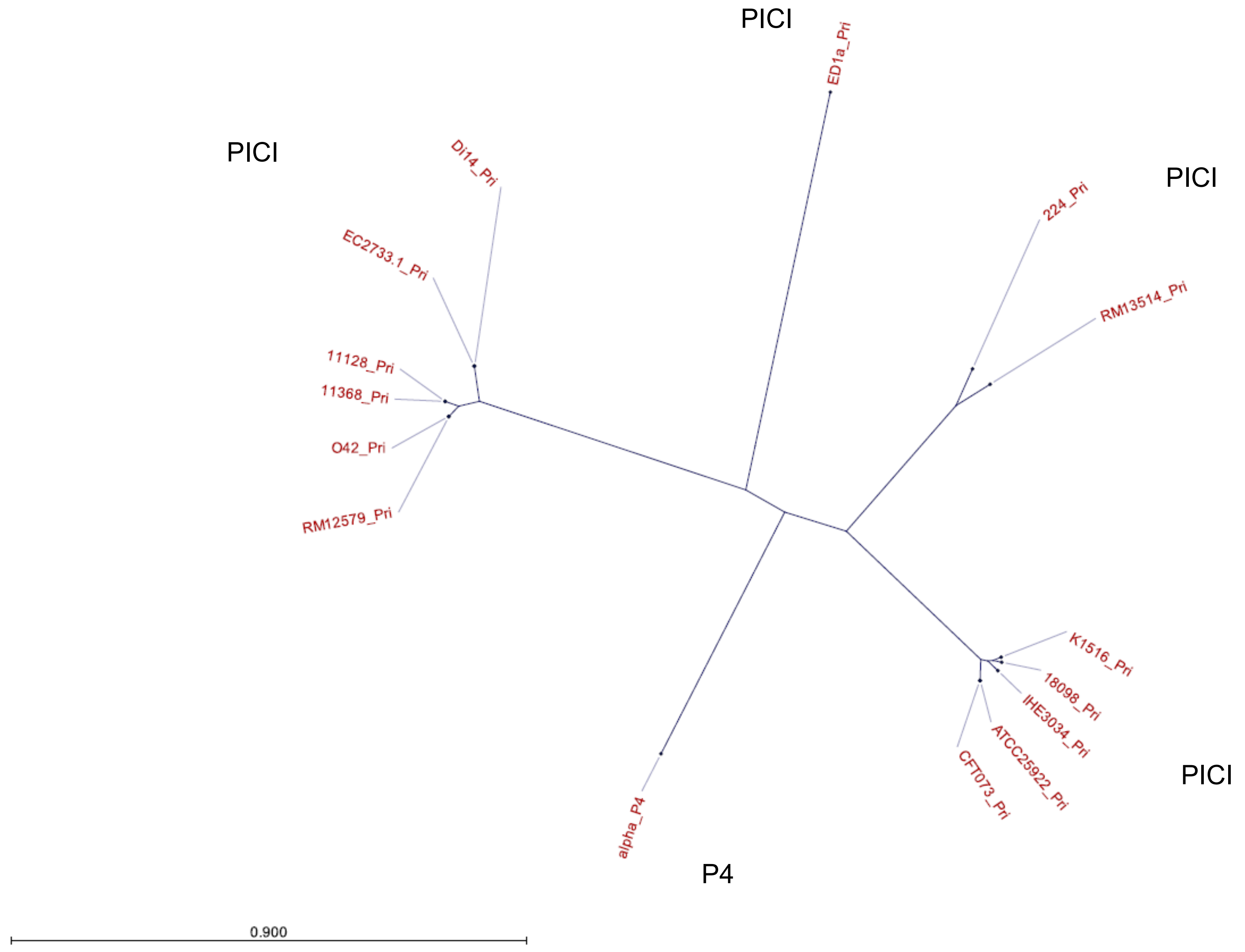
PICI
ED1a_Pri
PICI
Di14_Pri
EC2733.1_Pri
11128_Pri
11368_Pri
O42_Pri
RM12579_Pri
PICI
224_Pri
RM13514_Pri
K1516_Pri
18098_Pri
IHE3034_Pri
ATCC25922_Pri
CFT073_Pri
PICI
alpha_P4
P4
0.900

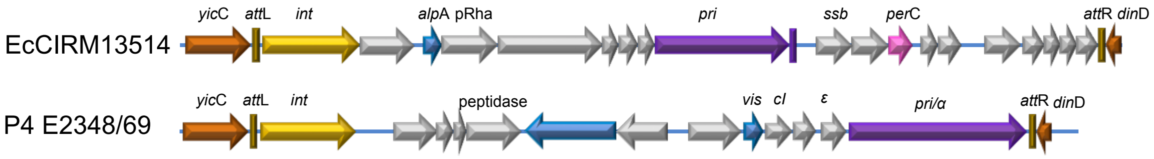

Figure S11. Genome comparison of the EcCI and P4 elements integrated at the same *attB* site. Genomes are aligned according to the prophage convention, with the integrase gene (*int*) at the left end. Genes are coloured according to their sequence and function: *int* is yellow; transcription regulator (*alp*A) is dark blue; replication genes are purple; encapsidation genes are green, with the terminase small subunit gene (*ter*S) in light green; superantigen and other virulence genes are pink; genes encoding putative phage resistance proteins are black; other accessory genes are red; genes encoding hypothetical proteins are white.
